# Single-cell transcriptomics reveals specific RNA editing signatures in the human brain

**DOI:** 10.1261/rna.058271.116

**Published:** 2017-06

**Authors:** Ernesto Picardi, David S. Horner, Graziano Pesole

**Affiliations:** 1 Department of Biosciences, Biotechnology and Biopharmaceutics, University of Bari, 70126 Bari, Italy; 2 Institute of Biomembranes and Bioenergetics, National Research Council, 70126 Bari, Italy; 3 Department of Biosciences, University of Milan, 20133 Milan, Italy

**Keywords:** RNA editing, single cell, RNA-seq, transcriptome

## Abstract

While RNA editing by A-to-I deamination is a requisite for neuronal function in humans, it is under-investigated in single cells. Here we fill this gap by analyzing RNA editing profiles of single cells from the brain cortex of living human subjects. We show that RNA editing levels per cell are bimodally distributed and distinguish between major brain cell types, thus providing new insights into neuronal dynamics.

## INTRODUCTION

Deep-sequencing technologies have allowed the characterization of patterns of transcriptome variation across individuals and tissues at unprecedented resolution—facilitating substantial advances in our understanding of transcriptional regulation and post-transcriptional processing (Mele et al. 2015). Recent advances in whole-transcriptome amplification have permitted quantitative sequencing of the minute amounts of RNA residing in single cells, and offer a unique opportunity to investigate transcriptional heterogeneity within tissues or even cell types by circumventing the need to study bulk tissue populations that usually comprise thousands or millions of input cells (Shapiro et al. 2013). Among human tissues, the adult brain shows extremely high complexity, being composed of a variety of cell classes and subtypes. Microfluidic systems coupled with massive mRNA sequencing on dissociated human brain cortex enable the identification of all major neuronal, glial, and vascular cell types, and reveal distinctive transcriptome profiles undetectable in the ensemble tissue (Darmanis et al. 2015). Despite these advances, cell level variation in post-transcriptional processing steps such as RNA modification by adenosine to inosine (A-to-I) editing, which actively contributes to transcriptome and proteome complexity, remains under-investigated.

A-to-I RNA editing is widespread in humans, affecting coding and noncoding transcripts at thousands of sites, and has a plethora of biological effects depending on the RNA context modified. RNA editing alters codon identity, creates or eliminates splice sites, and interferes with base-pairing interactions within higher-order RNA structures (Nishikura 2016). A-to-I deamination is catalyzed by members of the adenosine deaminase (ADAR) family of enzymes that act on dsRNA and occurs mainly in the primate-specific Alu repetitive elements (Nishikura 2016). ADARs are extremely important for the maintenance of cell homeostasis as mouse null mutants develop epileptic seizures and die several weeks after birth (Higuchi et al. 2000; Horsch et al. 2011). In addition, dysregulated RNA editing levels at specific recoding sites have been linked with a variety of diseases including neurological or psychiatric disorders and cancer (Chen et al. 2013; Behm and Öhman 2016; Khermesh et al. 2016).

We recently profiled A-to-I editing at the genome-wide level in various bulk human tissues and confirmed its pervasive nature, detecting more than 3 million events (Picardi et al. 2015). Our comprehensive catalog indicates strong tissue specificity of RNA editing and reveals the brain to be the human tissue with the most specific modifications. Indeed, A-to-I editing is required for neuronal function as many targets are key mediators of synaptic signaling (Mattick and Mehler 2008; Behm and Öhman 2016).

To capture and characterize the complexity of RNA editing at single-cell resolution, we exploited existing single cell RNA sequencing (scRNA-seq) data from adult human cortex cells obtained from living subjects (Darmanis et al. 2015). Our study provides novel and exciting insights into neuronal plasticity and opens up the possibility to decipher yet unknown molecular aspects of RNA editing in physiological as well as diverse neurological or neurodegenerative disorders.

## RESULTS AND DISCUSSION

We explored cell inosinome profiles using a comprehensive and nonredundant collection of known RNA editing events, including A-to-I changes from our catalog (Picardi et al. 2015) as well as sites annotated in the RADAR database (Ramaswami and Li 2014). The data allowed the interrogation of more than 4.5 million positions per cell.

Initially we processed raw scRNAseq data from 331 cells, performing quality checks, adaptor trimming, and in silico rRNA depletion. Cleaned reads were aligned onto the reference genome using the STAR mapper. Next, we excluded cells with fewer than 1 million uniquely aligned reads and a mapping rate <70%, reducing the starting data set to 268 cells. On average, we analyzed 2.3 million reads per cell (Supplemental Table 1), calling RNA editing events using REDItools (Picardi and Pesole 2013) and retaining only sites supported by at least 10 independent reads.

**FIGURE 1.**
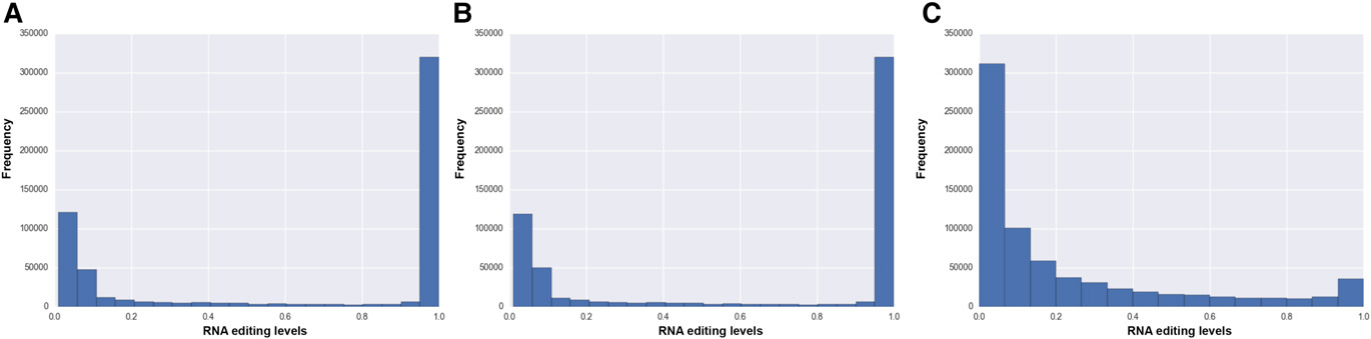
Distribution of RNA editing levels from all cells calculated with potential PCR duplicates (*A*), without potential PCR duplicates (*B*), and merging reads from all cells mimicking an ensemble tissue (*C*).

The number of detected A-to-I events varied greatly among cell types and within each cell population, principally as a consequence of variation in sequencing depth (Supplemental Table 1). Indeed, the number of A-to-I changes per cell was strongly correlated with the number of uniquely aligned reads (*r* = 0.83, *P* = 0.0) (Supplemental Fig. 1). Strikingly, RNA editing levels (proportions of reads supporting an editing event at each known editing site) for individual cells showed a bimodal distribution with peaks close to extreme values (0 and 1) (Fig. 1A). This observation was not an artifact resulting from the presence of PCR duplicate reads, as PCR duplication was globally low (affecting on average 10% of aligned reads) (Supplemental Fig. 2). Furthermore, raw and de-duplicated data sets shared, on average, 95% of candidate editing sites and by position, comparison of A-to-I levels showed a remarkable positive correlation (*r* = 0.9998, *P* = 0.0) (Supplemental Fig. 3). After removal of PCR duplicates, A-to-I editing levels of single cells continued to exhibit an extreme bimodal distribution (Fig. 1B). However, when scRNA-seq reads were merged, mimicking an ensemble tissue, RNA editing levels displayed a classical unimodal distribution in which the majority of A-to-I editing levels was lower than 0.2, as previously observed in six human tissues (including brain cortex) (Fig. 1C; Picardi et al. 2015). These observations suggest that the penetrance of editing at single sites in single brain cortex cells shows an “all or nothing” distribution and that the affected sites vary between single cells, an effect that is masked by the study of bulk tissues. The bimodal distribution of RNA editing levels was also recently shown for C-to-U editing levels in homogeneous populations of mice macrophages (Harjanto et al. 2016).

**FIGURE 2.**
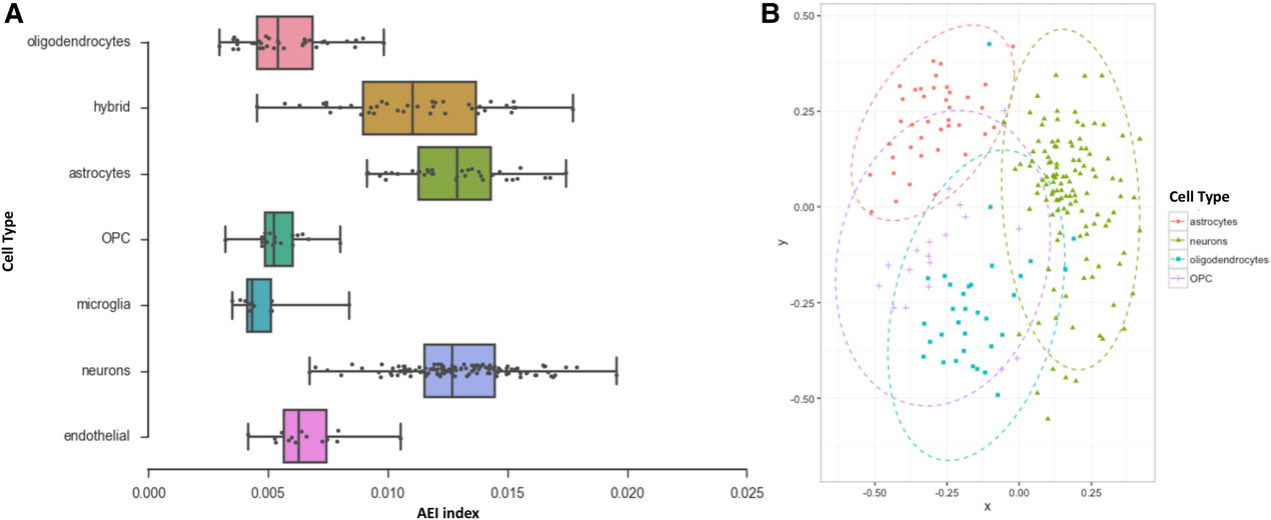
Distribution of AEI index across brain cell populations (*A*) and MDS analysis of RNA editing profiles in major brain cell types (*B*).

The vast majority of RNA editing sites detected here and in previous studies (Bazak et al. 2014a; Picardi et al. 2015) reside in Alu repetitive elements. To provide a more realistic estimate of global editing activity per cell, we calculated the Alu editing index (AEI) per cell because it represents the weighted average editing level across all expressed Alu sequences (Paz-Yaacov et al. 2015). We grouped AEI values per cell type according to transcriptome profiling previously characterized (Darmanis et al. 2015) and found that each cell type population exhibited a peculiar AEI distribution in which astrocytes and neurons appeared as the most edited cell types (Fig. 2A). To confirm cell specificity of RNA editing, we performed a multidimensional scaling (MDS) analysis using Spearman correlation coefficients calculated by pairwise comparisons of RNA editing levels in major brain cell types. Strikingly, four clusters emerge, corresponding to astrocytes, neurons, oligodendrocytes, and OPCs (oligodendrocyte precursor cells); the overlap between the latter cell types indicating similar RNA editing profiles (Fig. 2B; Supplemental Fig. 4 including all cell types).

We also checked the expressed levels of ADAR enzymes in all cell types and found a very uneven distribution for *ADAR* gene (also known as *ADAR1*), while *ADARB1* (known as *ADAR2*) was expressed at very low levels but a bit more in neurons (Supplemental Fig. 5). Interestingly, the *ADARB2* gene, with questionable editing activity (Chen et al. 2000), was expressed at detectable levels only in neurons, oligodendrocytes, and hybrid cells (Supplemental Fig. 5). However, the expression levels of *ADAR*s did not correlate with RNA editing levels and AEI values as well. In contrast, the AEI index showed strong correlation with the mean editing levels per cell (Spearman correlation value of 0.76, *P*-value = 8.2 × 10^−53^) or the normalized sum of editing levels per cell (Spearman correlation value of 0.80, *P*-value = 1.7 × 10^−61^). The absence of significant correlation between the expression levels of *ADAR*s and RNA editing levels or AEI values in single cells may be due to specific regulation mechanisms acting on ADAR enzymes or to a biased detection of gene expression levels. Indeed, many factors, such as cell cycle or cell size, may affect the correct quantification of gene expression levels, especially in cases of low expressed genes (Wu et al. 2014; Kowalczyk et al. 2015; Padovan-Merhar et al. 2015). Additional experimental evidence will be required to clarify this aspect.

**FIGURE 3.**
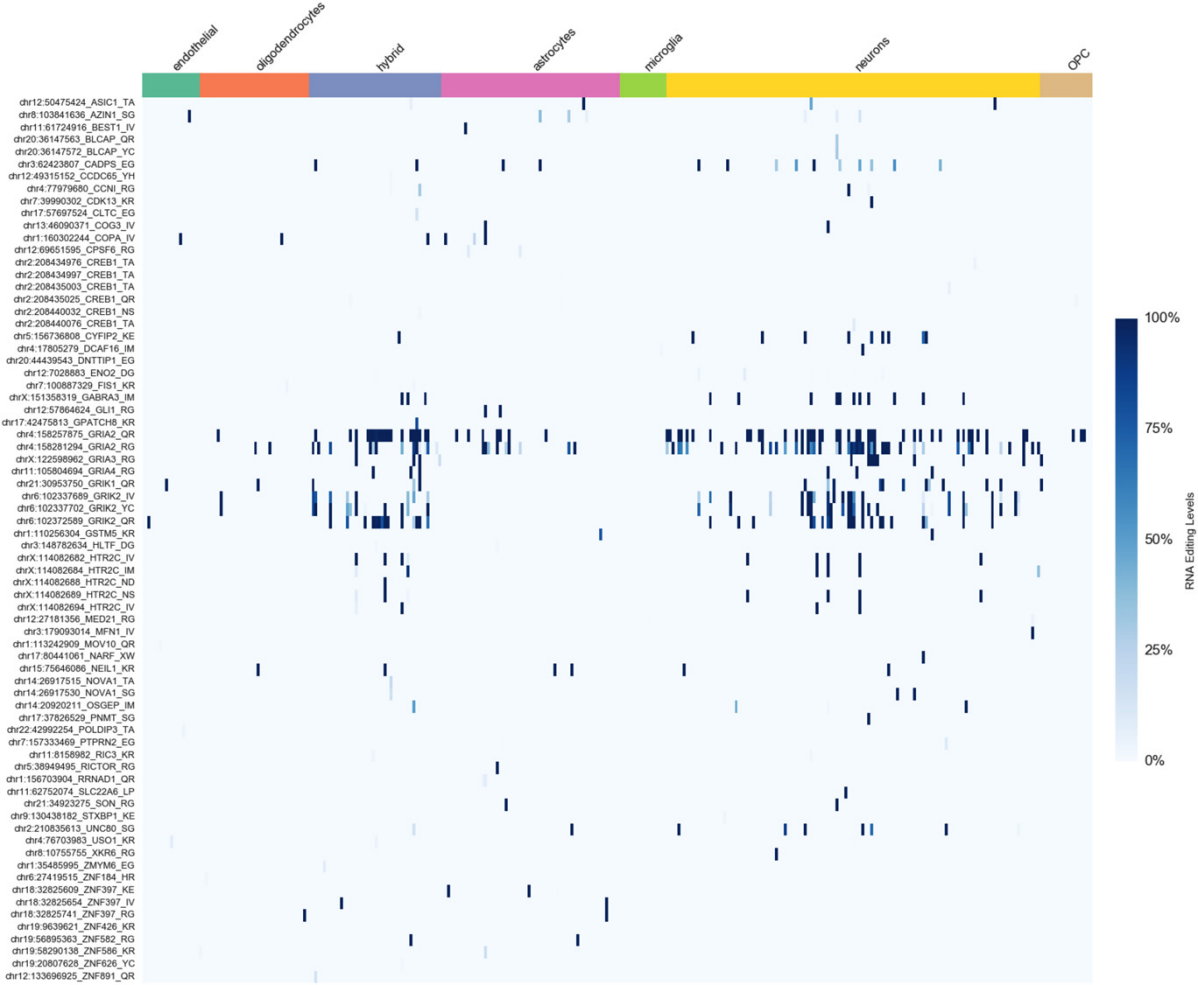
Heatmap of RNA editing levels at recoding sites in brain cells (see Supplemental Table 3 for details).

Historically, most interest has been directed toward recoding sites, where RNA editing results in amino acid substitutions. Many of the best-characterized recoding sites are in brain-specific transcripts coding for membrane receptors and ion channels (Behm and Öhman 2016). For 183 such positions selected from our editing collection and the RADAR database, RNA editing activity was higher in neurons than other cell types (Fig. 3). However, only a few sites, located in genes for glutamate receptors, were edited in almost all neurons, suggesting a universally critical role for editing at these sites (Supplemental Table 3). The data set used in this study also included a new hypothetical cell type indicated as “hybrid” that displayed characteristics of both neurons and astrocytes (Darmanis et al. 2015). The analysis of recoding sites indicated that hybrid cells were more similar to neurons than astrocytes (Fig. 3). “Hybrid” cells exhibited a recoding editing pattern of glutamate receptors that overlaps substantially with that observed in neurons (Fig. 3). In addition, MDS analysis of RNA editing profiles from “hybrid” cells, neurons, and astrocytes showed large overlap between “hybrid” cells and neurons but not astrocytes (Supplemental Figs. 6, 7). Thus, our results support the view of “hybrid” cells as a potential new cell type related to neurons.

To further investigate the possibility that RNA editing profiles represent powerful signatures of cell type specificity, we analyzed single fetal brain cells, since they are considerably different from any cell type in the adult brain and because RNA editing efficiency increases during brain development and, consequently, different editing patterns are expected between fetal and adult cells (Wahlstedt et al. 2009). We interrogated 4.5 million known RNA editing positions in 135 scRNA-seqs from previously characterized fetal brain cells (Darmanis et al. 2015). They include 110 quiescent fetal neurons and 25 actively dividing fetal neuronal progenitors. AEI distributions clearly showed elevated editing activity in adult neurons compared to fetal neurons and higher editing in quiescent fetal neurons compared to fetal neuronal progenitors (Supplemental Fig. 8A). MDS analysis of RNA editing profiles confirmed three main groups, distinguishing adult from fetal neurons (Supplemental Fig. 8B). Notably, RNA editing activity at recoding sites was higher in adult than fetal neurons (Supplemental Fig. 9). In particular, the Q/R site in Gria2, linked to neurological disorders, was edited to high levels in fetal quiescent neurons but not in neuronal progenitors as previously assessed in vitro (Pachernegg et al. 2015).

Taken together, our results demonstrate that RNA editing is detectable in single cells and demonstrates that A-to-I patterns reveal specific editing signatures distinguishing major cell types in the human brain. Profiling RNA editing in single cells may shed light on novel physiological roles of RNA editing in neuronal plasticity and in a variety of neurological/neurodegenerative disorders. In addition, A-to-I changes in single cells may contribute to the identification of novel therapeutic targets or prognostic markers for innovative approaches of precision medicine.

## MATERIALS AND METHODS

### Data sets

RNA-seq data of human brain single cells were downloaded from the Gene Expression Omnibus (GEO) database (www.ncbi.nlm.nih.gov/geo) using the accession number GSE67835. They comprise 332 cells from dissociated adult brain cortex and 134 cells from fetal brain tissue. Single cell capturing has been performed using Fluidigm C1 technology and RNA sequencing has been carried out on an Illumina NextSeq platform by Darmanis et al. (2015).

### Alignment of RNA-seq data

RNA-seq reads in FASTQ format were inspected using the FASTQC program. Adaptors and low quality regions (phred cutoff of 25) were trimmed using TrimGalore (http://www.bioinformatics.babraham.ac.uk/projects/trim_galore/), excluding reads with final length less than 50 bases. In addition, we removed read pairs displaying positive alignments with known human rRNA sequences. In brief, human rRNA annotations were downloaded from UCSC (hg19 human assembly) and indexed for STAR. Next, RNA reads were aligned onto rRNAs by STAR and only unmapped reads were retained for downstream analyses.

For each cell, cleaned reads were aligned onto the complete UCSC hg19 human genome by means of STAR (using as main parameters: --outSAMstrandField intronMotif --outFilterType BySJout --outFilterMultimapNmax 1 --alignSJoverhangMin 8 --alignSJDBoverhangMin 1 --outFilterMismatchNmax 999 --outFilterMismatchNoverLmax 0.04 --alignIntronMin 20 --alignIntronMax 1000000 --alignMatesGapMax 1000000).

Unique and concordant alignments in BAM format were processed by the CollectRnaSeqMetrics.jar tool from the Picard package to obtain basic statistics (Supplemental Table 1). Cells with fewer than 1 million uniquely aligned reads and a mapping rate <70% were excluded from further analyses.

Duplicated reads were removed using the MarkDuplicates.jar tool from the Picard package. Since duplication was very low and did not affect RNA editing levels, we performed all RNA editing analyses on RNA-seq data with duplicates.

### RNA editing calling and data analysis

In the absence of a whole-genome data set, we explored RNA editing profiles per cell comparing RNA-seq data with a comprehensive and nonredundant collection of known events, including A-to-I changes from our catalog (Picardi et al. 2015) as well as sites annotated in the RADAR database (Ramaswami and Li 2014). In all, we interrogated more than 4.5 million positions per cell using our REDItools suite. The complete collection is freely available through the REDIportal database (Picardi et al. 2016).

For each cell, REDItoolDnaRna.py script was called using the following parameters: -c 0,0 -T ALLediting.sorted.gtf.gz –p –u -v0 -n0 -G ALLediting.sorted.gtf.gz -e -m20,20 -q30,30. REDItool tables were finally parsed to retain only edited positions supported by at least 10 reads.

To measure the global editing activity per cell, we calculated the Alu editing index, the weighted average editing level across all expressed Alu sequences, using custom scripts and according to the methodology described in Bazak et al. (2014b).

RNA editing in recoding sites was assessed using REDItools and provided a list of 183 known positions in which RNA editing causes amino acid change. Such positions, listed in Supplemental Table 3, have been selected from our RNA editing Atlas as well as the RADAR database, picking only sites residing in nonrepetitive genomic regions and following criteria already described in Khermesh et al. (2016). As an exception, we included a NARF recoding site residing in an Alu region, since that site has already been validated in the literature (Moller-Krull et al. 2008).

Main statistical analyses and plots were performed using pandas, scipy, and matplotlib modules in python. Multidimensional scaling (MDS) was carried out in R using the metaMDS function of the *vegan* package, providing as input a Spearman correlation matrix calculated from editing levels for each cell type. Two-dimensional images depicting MDS clusters were generated by *ggplot2*.

## SUPPLEMENTAL MATERIAL

Supplemental material is available for this article.

## Supplementary Material

Supplemental Material
