# Supplementary material for: Single-cell transcriptomics reveals specific RNA editing signatures in the human brain
Source: RNA. 2017 Jun;23(6):860–5. doi: 10.1261/rna.058271.116 (PMC5435858; doi:10.1261/rna.058271.116)
Supplement: Supplemental Material [file supp_23_6_860__index.html]

Single-cell transcriptomics reveals specific RNA editing signatures in the human brain — Supplemental Material 

# Single-cell transcriptomics reveals specific RNA editing signatures in the human brain

## Supplemental Material

- Supplemental\_Figures.pdf
- Supplemental\_Table\_1.pdf
- Supplemental\_Table\_2.pdf
- Supplemental\_Table\_3.pdf
