# Supplementary material for: Single-cell transcriptomics reveals specific RNA editing signatures in the human brain
Source: RNA. 2017 Jun;23(6):860–5. doi: 10.1261/rna.058271.116 (PMC5435858; doi:10.1261/rna.058271.116)
Supplement: Supplemental Material [file supp_058271.116_Supplemental_Figures.pdf]

Single cell transcriptomics reveals specific RNA editing signatures in the human brain.

Ernesto Picardi[1,2], David S. Horner[3] and Graziano Pesole[1,2,*]

[1] Department of Biosciences, Biotechnology and Biopharmaceutics, University of Bari, Via Orabona 4, 70126 Bari, Italy
[2] Institute of Biomembranes and Bioenergetics, National Research Council, Via Amendola 165/A, 70126 Bari, Italy
[3] Department of Biosciences, University of Milan, Via Celoria 26, 20133 Milan, Italy

Supplementary Figures

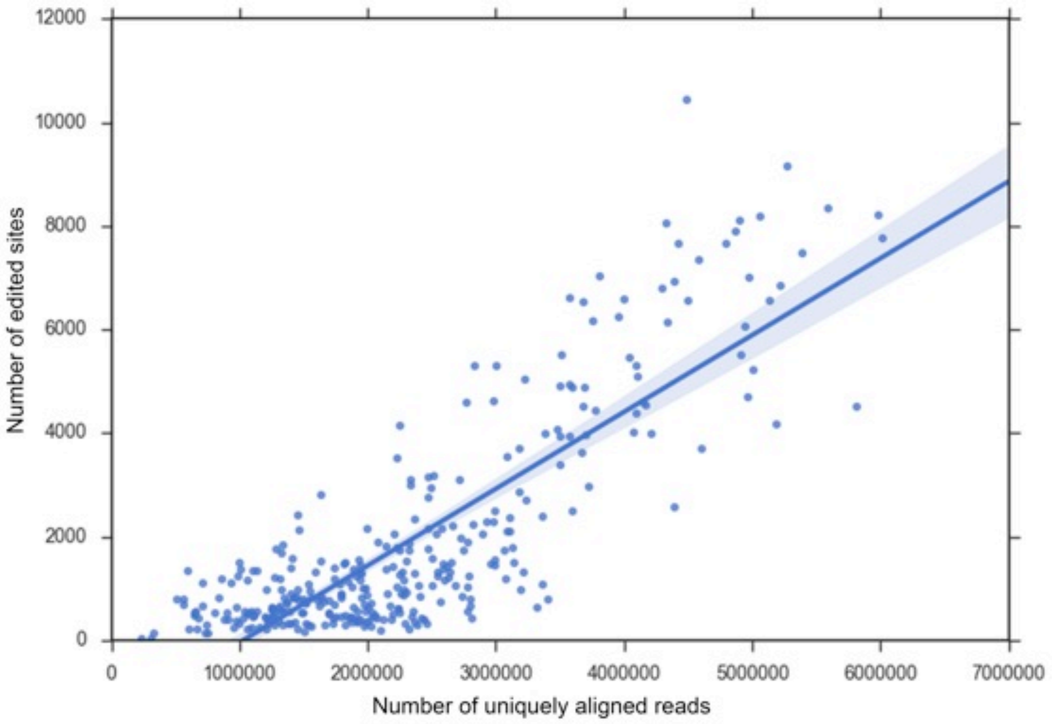

Supplementary Figure 1. Scatter plot showing the relationship between uniquely aligned reads and the number of detected RNA editing events per cell (r=0.83, P=0.0).

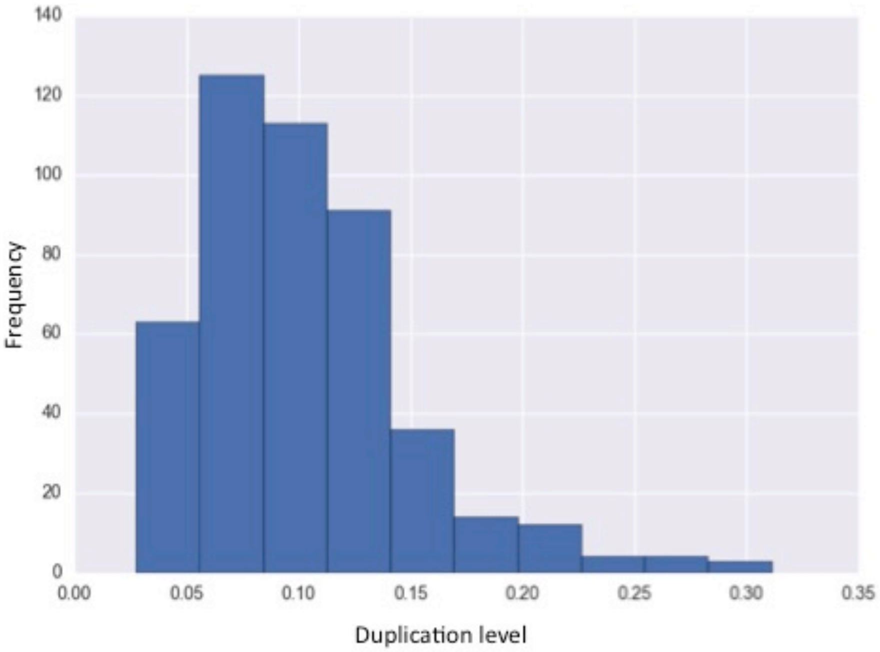

Supplementary Figure 2. Distribution of PCR duplication rate per each cell.

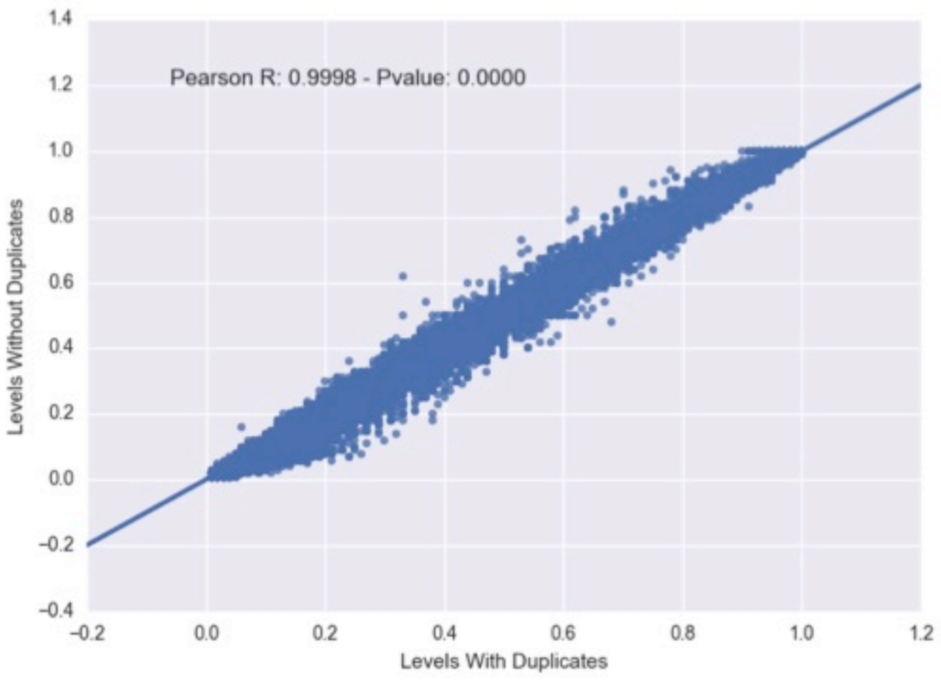

Supplementary Figure 3. Comparison between RNA editing levels calculated with and without potential PCR duplicates.

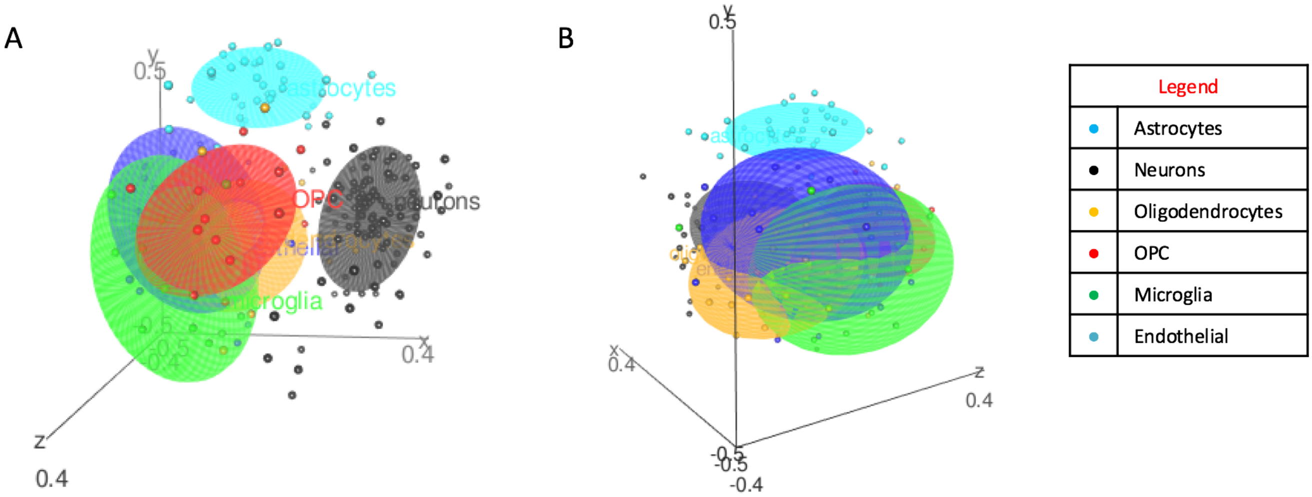

Supplementary Figure 4. 3D images of MDS analysis on RNA editing profiles in all brain cell types. In A, astrocytes, neurons and OPC cells show different groupings. In B, endothelial and microglia cells exhibit overlapping clusters meaning similar RNA editing profiles. Images were generated in R using *rgl* and *car* packages.

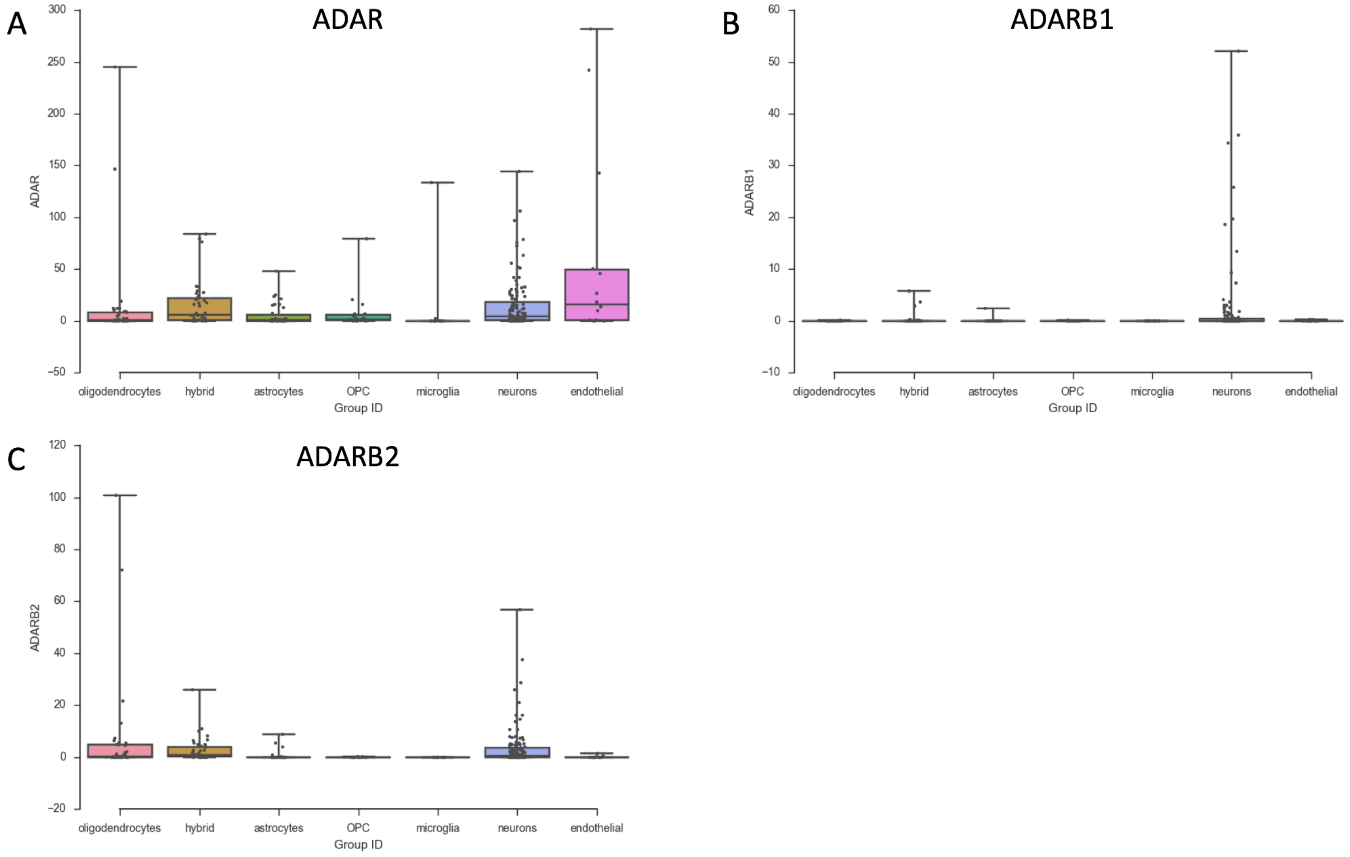

Supplementary Figure 5. Expression levels of ADAR genes. In A, ADAR gene expression shows a very uneven distribution while ADARB1 (in B) is expressed at very low levels but a bit more in neurons. In C, ADARB2 gene expression is detectable only in neurons, oligodendrocytes and hybrid cells.

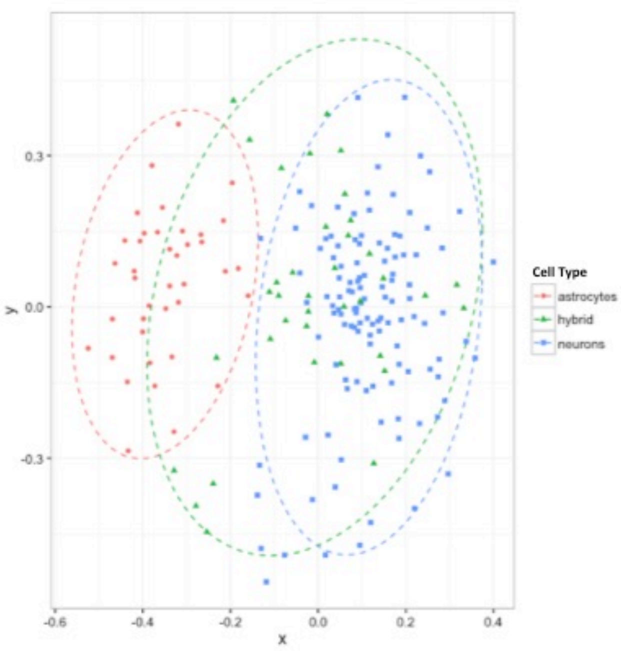

Supplementary Figure 6. Multidimensional scaling of RNA editing profiles in astrocytes, neurons and 'hybrid' cells.

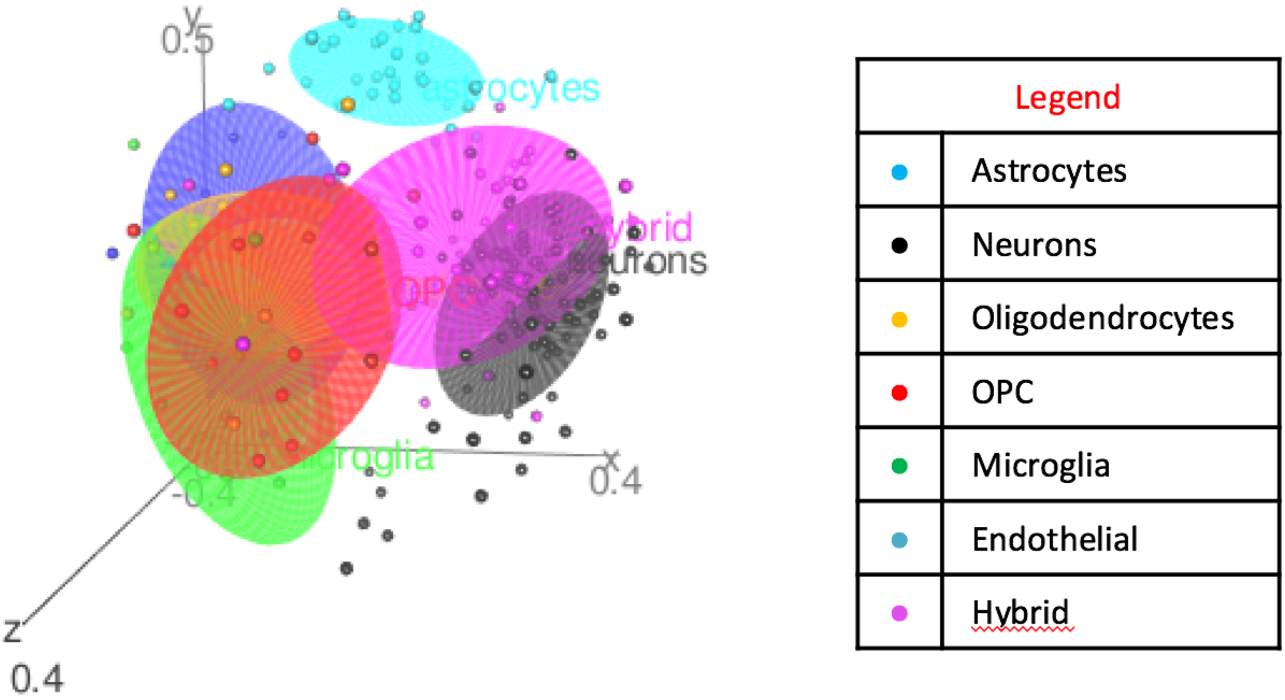

Supplementary Figure 7. 3D image of MDS analysis on RNA editing profiles in all brain cell types, generated in R using *rgl* and *car* packages. In contrast with Supplementary Fig. 4, here we included hybrid cells. As shown, such cell type is in overlap with neurons meaning that hybrid cells are more similar to neurons than astrocytes.

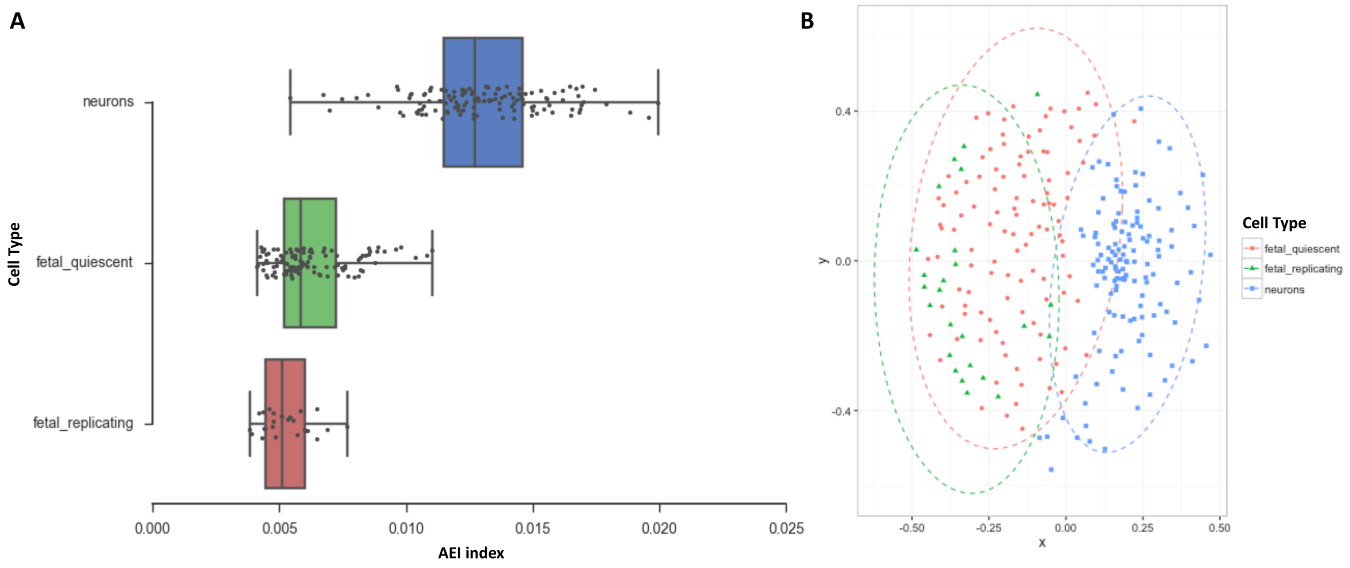

Supplementary Figure 8. Distribution of AEI index in fetal and adult neurons (A) and MDS analysis of RNA editing profiles in fetal brain cells (B).

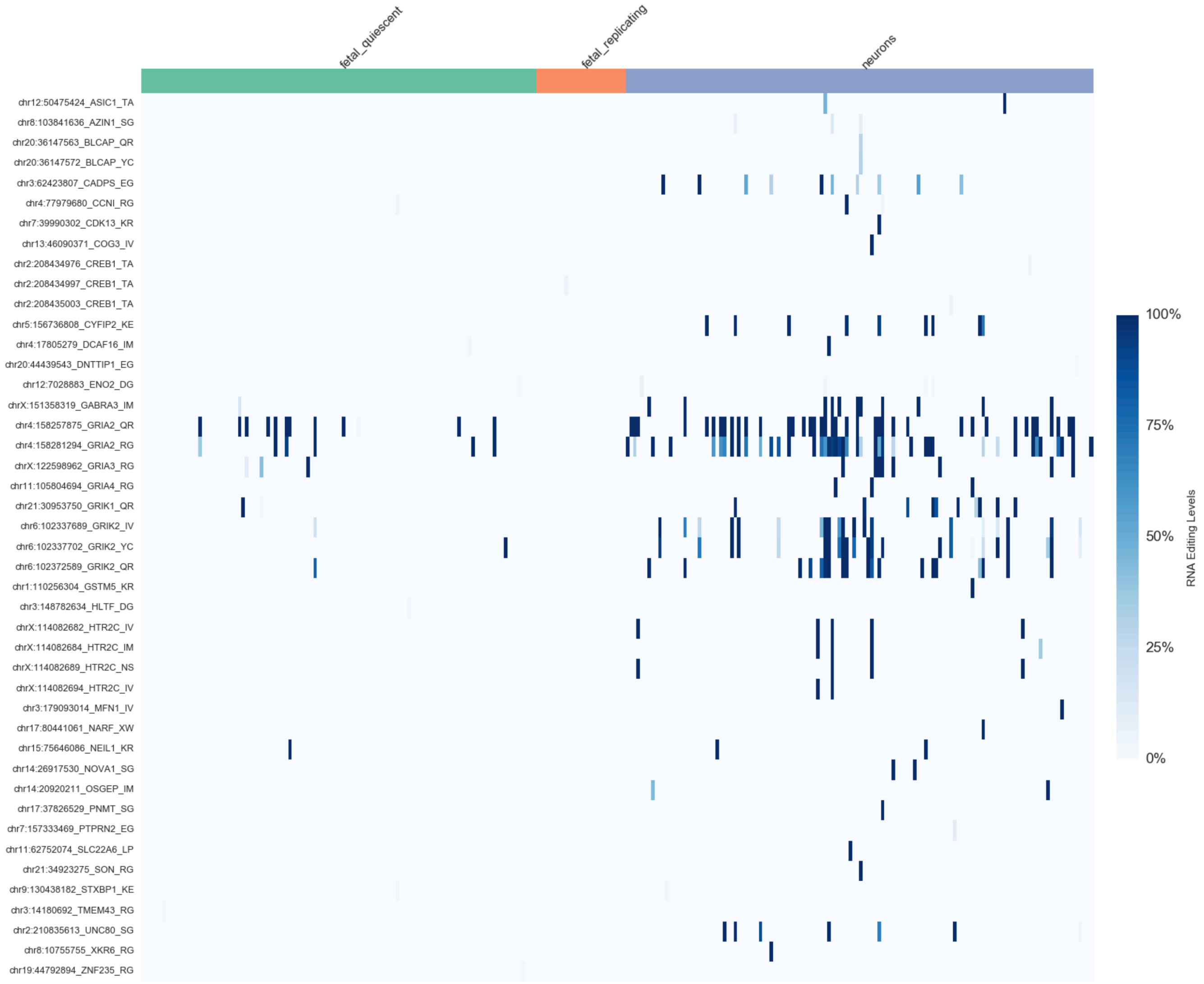

Supplementary Figure 9. Heatmap of RNA editing levels at recoding sites in fetal brain cells.

Supplementary Tables

Supplementary Table 1. Main statistics calculated per single cell RNAseq experiment including the total number of reads obtained along each step of our computational analysis. Statistics reported in the last 22 columns were calculated using Picard tools. Their explanation is available at https://broadinstitute.github.io/picard/picard-metric-definitions.html#RnaSeqMetrics. The explanation of first 8 columns is as follow:

SRArun: SRA accession number;
Group: cell type;
Reads: number of raw reads;
trimG: number of reads after the trimming procedure by TrimGalore;
rRNA: number of reads without rRNA contamination;
STARin: number of reads provided as input to STAR program for mapping;
MapAsUnique: number of uniquely mapped reads;
%Mapped: fraction of uniquely mapped reads.

Supplementary Table 2. Per each cell we report the number of editing sites detected with and without PCR duplicates. In addition, we include the fraction of common RNA editing sites as well as the Pearson correlation coefficient (and Pvalue) of editing levels. Column explanation is as follow:

SRR: SRA accession number;
EditingDup: number of RNA editing events detected in presence of read duplicates;
EditingNoDup: number of RNA editing events detected in absence of read duplicates;
EditingCommon: number of common RNA editing events;
DiffNumSiti: number of different RNA editing events;
FractionOfCommon: fraction of common RNA editing events;
PearsonR: Pearson Correlation coefficient between sites in presence and absence of read duplicates;
Pvalue: P. value of the Pearson Correlation coefficient;
Reads: number of raw reads;
Unique: number of uniquely mapped reads;
Mapped: fraction of uniquely mapped reads.

Supplementary Table 3. List of known recoding RNA editing sites. The amino acid change and the corresponding annotation are also reported. We also provide additional info (extended gene name, cellular location and type) about target genes.
