# Supplementary material for: Single-cell transcriptomics reveals specific RNA editing signatures in the human brain
Source: RNA. 2017 Jun;23(6):860–5. doi: 10.1261/rna.058271.116 (PMC5435858; doi:10.1261/rna.058271.116)
Supplement: Supplemental Material [file supp_058271.116_Supplemental_Table_1.pdf]

SRArun	Group	Reads	trimG	rRNA	STARin	MapAsUnique	%Mapped	READ_PAIR_OPTICAL_DUPLICATES	PERCENT_DUPLICATION	ESTIMATED_LIBRARY_SIZE	PCT_RIBOSOMAL_BASES	PCT_CODING_BASES	PCT_UTR_BASES	PCT_INTRONIC_BASES	PCT_INTERGENIC_BASES	PCT_MRNA_BASES	PCT_USABLE_BASES	MEDIAN_CV_COVERAGE
SRR1974557	astrocytes	2506473	2407820	2232039	2232039	1923413	86,17291185	0	0,191328	4362512	0	0,061615	0,165533	0,577088	0,195764	0,227148	0,225473	2,2764
SRR1974604	astrocytes	2601321	2463078	2313431	2313431	1940442	83,87723688	0	0,103938	8675910	0,000002	0,196686	0,27291	0,421706	0,108696	0,469596	0,465168	0,985004
SRR1974610	astrocytes	2830982	2678538	2498536	2498536	2143535	85,79163958	0	0,097785	10233672	0	0,087364	0,138157	0,601855	0,172624	0,225521	0,223463	1,300878
SRR1974637	astrocytes	4248628	4029324	3791034	3791034	3147101	83,01431747	0	0,130806	10956017	0,000001	0,191779	0,201683	0,47559	0,130946	0,393462	0,38978	1,230047
SRR1974641	astrocytes	4987406	4699241	4394381	4394381	3362601	76,52047012	0	0,187349	7814306	0	0,16068	0,279346	0,429201	0,130773	0,440026	0,43575	1,771162
SRR1974647	astrocytes	4267586	4024644	3764739	3764739	3135038	83,27371433	0	0,131709	10831652	0,000001	0,137062	0,220945	0,502493	0,139498	0,358008	0,354647	1,333619
SRR1974651	astrocytes	3750049	3521254	3274232	3274232	2691176	82,19258745	0	0,132023	9273724	0	0,185427	0,285459	0,4008	0,128314	0,470887	0,465675	1,255549
SRR1974653	astrocytes	3712300	3480502	3240812	3240812	2783231	85,88066818	0	0,108558	11873518	0,000002	0,159137	0,243314	0,476613	0,120935	0,402451	0,39822	0,874479
SRR1974664	astrocytes	1987160	1620191	1502602	1502602	867746	57,7495571	0	0,095809	4234393	0,000001	0,062948	0,118696	0,679846	0,138508	0,181644	0,179696	2,796832
SRR1974670	astrocytes	1955412	1631102	1499653	1499653	1017013	67,8165549	0	0,110979	4236334	0	0,078677	0,177356	0,576177	0,167791	0,256032	0,254285	2,67336
SRR1974672	astrocytes	1771521	1490268	1369387	1369387	934614	68,25053838	0	0,111701	3865853	0	0,055833	0,19536	0,589031	0,159776	0,251193	0,249446	2,749757
SRR1974678	astrocytes	6047654	5701966	5506624	5506624	4004410	72,71987337	0	0,121064	15182086	0,000002	0,171041	0,208511	0,507608	0,112839	0,379552	0,375095	0,87735
SRR1974679	astrocytes	5492700	5180462	4998711	4998711	3576358	71,54560446	0	0,100457	16598595	0,000001	0,132515	0,139143	0,604401	0,12394	0,271658	0,26852	1,061284
SRR1974682	astrocytes	5628943	5265809	5034550	5034550	3685746	73,2090455	0	0,106353	16086501	0,000019	0,124035	0,149676	0,565638	0,160631	0,273711	0,27045	0,99941
SRR1974688	astrocytes	5329477	5001129	4819598	4819598	3479052	72,18552253	0	0,131373	12060302	0,000004	0,150484	0,205489	0,512486	0,131536	0,355973	0,352053	1,059289
SRR1974689	astrocytes	3659877	3397928	3238324	3238324	2335731	72,1277735	0	0,129127	8251663	0	0,132763	0,200121	0,534924	0,132192	0,332883	0,329104	1,18782
SRR1974692	astrocytes	7813695	7396987	7152032	7152032	5135631	71,80659986	0	0,139173	16703469	0	0,123324	0,151814	0,566582	0,15828	0,275138	0,272148	1,197053
SRR1974693	astrocytes	4961238	4682993	4517788	4517788	3185009	70,49930187	0	0,13068	11105476	0,000009	0,127073	0,158187	0,574235	0,140496	0,28526	0,281728	1,085358
SRR1974698	astrocytes	5855636	5507794	5290649	5290649	3699685	69,92875543	0	0,129626	13014763	0,000022	0,12843	0,159678	0,582373	0,129497	0,288108	0,284724	1,217026
SRR1974699	astrocytes	5608933	5259140	5024174	5024174	3510691	69,87598359	0	0,12675	12659754	0,000005	0,099344	0,134276	0,65767	0,108706	0,23362	0,230784	1,578215
SRR1974701	astrocytes	6609556	6256255	6032216	6032216	4347178	72,06602018	0	0,123086	16186565	0,000017	0,166032	0,191753	0,533894	0,108304	0,357785	0,353541	0,931542
SRR1974703	astrocytes	5952629	5591965	5393199	5393199	3703660	68,67278585	0	0,134001	12563199	0	0,141762	0,158604	0,553104	0,14653	0,300366	0,296791	1,420396
SRR1974715	astrocytes	7799671	7374799	7086011	7086011	4914925	69,3609564	0	0,142416	15581830	0,000006	0,180094	0,226777	0,465175	0,127949	0,406871	0,402061	1,020043
SRR1974717	astrocytes	6083848	5713985	5458426	5458426	3573399	65,46574049	0	0,147399	10904512	0,000001	0,09994	0,137833	0,608389	0,153838	0,237773	0,234928	1,677169
SRR1974720	astrocytes	4366007	4069231	3903615	3903615	2776386	71,12345864	0	0,104322	12372726	0	0,076862	0,133478	0,655202	0,134458	0,21034	0,207982	1,401961
SRR1974721	astrocytes	5093737	4777844	4560389	4560389	2717041	59,57914994	0	0,142348	8620775	0	0,088508	0,130208	0,614768	0,166516	0,218716	0,216118	2,072192
SRR1974722	astrocytes	6770615	6405817	6145558	6145558	4145772	67,45965134	0	0,139866	13410926	0,000006	0,152062	0,188622	0,5243	0,135009	0,340684	0,336732	1,119786
SRR1974723	astrocytes	6679883	6252187	5996266	5996266	4303111	71,76317728	0	0,117585	16844014	0,00003	0,104831	0,13652	0,620038	0,138582	0,24135	0,238455	1,106368
SRR1974725	astrocytes	5743694	5331514	5091842	5091842	3499458	68,72675939	0	0,126529	12642220	0,000006	0,096672	0,166751	0,554668	0,181904	0,263423	0,260162	1,398091
SRR1974726	astrocytes	4587919	4274311	4093120	4093120	2981628	72,84487139	0	0,117587	11670984	0	0,126839	0,159772	0,594148	0,119241	0,286611	0,283174	1,14928
SRR1974727	astrocytes	5251312	4877377	4457791	4457791	2330606	52,28163456	0	0,31139	2916438	0	0,11121	0,140158	0,374469	0,374163	0,251368	0,248075	2,09666
SRR1974728	astrocytes	6745907	6371677	6117607	6117607	4099346	67,00897916	0	0,158941	11493218	0,000001	0,086373	0,15544	0,606573	0,151614	0,241813	0,239109	1,650429
SRR1974731	astrocytes	8084208	7599730	7325598	7325598	4971404	67,86345633	0	0,155704	14266266	0	0,130749	0,164403	0,595687	0,10916	0,295153	0,291788	1,21244
SRR1974732	astrocytes	7873178	7423009	7147946	7147946	5278402	73,84501786	0	0,114863	21191929	0,000001	0,103808	0,11935	0,639303	0,137538	0,223158	0,220706	1,008854
SRR1974733	astrocytes	5634272	5358419	5196183	5196183	3685440	70,9259085	0	0,139919	11916000	0	0,117815	0,14998	0,61884	0,113365	0,267795	0,264628	1,151127
SRR1974734	astrocytes	4133301	3906104	3744213	3744213	2338743	62,46287271	0	0,134989	7868195	0,000003	0,09161	0,161128	0,565111	0,182148	0,252738	0,249562	1,89267
SRR1974736	astrocytes	3803216	3553102	3417205	3417205	2470730	72,3026567	0	0,109993	10396747	0,000001	0,129479	0,195481	0,549385	0,125655	0,32496	0,320922	1,017808
SRR1974737	astrocytes	6454831	6070296	5838587	5838587	3727584	63,84394032	0	0,154895	10760298	0	0,210717	0,208177	0,478998	0,102108	0,418894	0,41374	1,290734
SRR1974738	astrocytes	4290579	4055311	3840904	3840904	2477624	64,50627248	0	0,13591	8273380	0	0,117779	0,166495	0,527367	0,188359	0,284274	0,280873	1,675113
SRR1974739	astrocytes	6471254	6097682	5898608	5898608	4390064	74,42542376	0	0,122766	16390632	0	0,093274	0,120006	0,643523	0,143197	0,21328	0,210835	1,112366
SRR1974740	astrocytes	5666331	5235212	4998398	4998398	3387758	67,77687571	0	0,134767	11418494	0	0,090523	0,133094	0,629266	0,147116	0,223618	0,220793	1,644696
SRR1974741	astrocytes	6104836	5763311	5545294	5545294	3677408	66,31583465	0	0,13805	12070391	0	0,155963	0,173566	0,551852	0,118619	0,329529	0,325528	1,313732
SRR1974743	astrocytes	8409889	7980097	7714629	7714629	5190908	67,2865539	0	0,166519	13808760	0	0,179623	0,204566	0,515583	0,100229	0,384188	0,379926	1,220201
SRR1974744	astrocytes	5553856	5250963	5095037	5095037	3183969	62,49157759	0	0,162194	8726001	0	0,187914	0,178378	0,520453	0,113255	0,366292	0,36186	1,529301
SRR1974745	astrocytes	7889393	7448214	7189219	7189219	5220341	72,61346469	0	0,144017	16344881	0	0,168307	0,181209	0,545426	0,105058	0,349516	0,345517	1,023528
SRR1974750	astrocytes	3756993	3526754	3365167	3365167	2499184	74,2662697	0	0,098019	11912183	0	0,198782	0,260365	0,42149	0,119363	0,459147	0,45359	0,883131
SRR1974751	astrocytes	7390496	6900350	6632792	6632792	4906260	73,96975512	0	0,119653	18840496	0	0,136715	0,174518	0,5686	0,120167	0,311233	0,307561	0,907338
SRR1974753	astrocytes	2709438	2561771	2448000	2448000	1339268	54,70866013	0	0,133125	4574438	0	0,394853	0,1713	0,336204	0,097642	0,566154	0,557787	1,399413
SRR1974754	astrocytes	7155864	6774432	6504017	6504017	4215012	64,80628818	0	0,148435	12761610	0	0,18437	0,22463	0,452226	0,138774	0,409	0,404316	1,227029
SRR1974814	astrocytes	1307265	1230282	1138430	1138430	962123	84,5131453	0	0,044595	10464160	0,000051	0,112821	0,229464	0,5369	0,120764	0,342285	0,339029	1,158665
SRR1974840	astrocytes	3441343	3290147	3064859	3064859	2547971	83,13501535	0	0,116035	10112425	0,000006	0,145013	0,259398	0,477067	0,118516	0,404411	0,401291	1,149169
SRR1974846	astrocytes	3511047	3358045	3131352	3131352	2660296	84,95678544	0	0,102182	12114658	0,000002	0,08831	0,166713	0,622761	0,122213	0,255023	0,253094	1,329976
SRR1974856	astrocytes	2189194	1818309	1665871	1665871	1286675	77,23737312	0	0,067438	9105771	0	0,164455	0,248822	0,451621	0,135102	0,413277	0,410439	1,505265
SRR1974857	astrocytes	1067477	873014	806533	806533	566375	70,22341305	0	0,048304	5672295	0	0,068297	0,145548	0,585733	0,200422	0,213845	0,212371	2,201151
SRR1974858	astrocytes	1838883	1518471	1396758	1396758	987222	70,67953074	0	0,060615	7810932	0	0,064611	0,091208	0,603011	0,24117	0,155819	0,15474	2,526564
SRR1974859	astrocytes	811230	699378	647699	647699	518959	80,12348328	0	0,035881	7057537	0	0,189313	0,287325	0,406881	0,116481	0,476638	0,473497	1,261348
SRR1974862	astrocytes	1846010	1532145	1416434	1416434	1063715	75,09809846	0	0,068835	7367741	0	0,180549	0,248523	0,416304	0,154624	0,429072	0,427126	1,622004
SRR1974863	astrocytes	1249134	1033893	954943	954943	717524	75,13788781	0	0,046031	7552947	0	0,06027	0,104643	0,607214	0,227872	0,164914	0,163773	2,202697
SRR1974865	astrocytes	2007129	1667537	1541482	1541482	1134484	73,59696707	0	0,076819	7000918	0,000003	0,132148	0,188743	0,53122	0,147886	0,320891	0,319048	1,919236
SRR1974871	astrocytes	2184245	1858513	1729089	1729089	1472965	85,18734432	0	0,05321	13345666	0,000001	0,089406	0,135021	0,657563	0,118008	0,224427	0,223275	1,813042
SRR1974873	astrocytes	2138540	1814680	1684437	1684437	1406365	83,49169485	0	0,059557	11333270	0	0,23193	0,251553	0,410473	0,106044	0,483483	0,481015	1,005716
SRR1974874	astrocytes	1783351	1503796	1389707	1389707	1107395	79,68550205	0	0,056099	9497276	0	0,100089	0,175778	0,590494	0,133639	0,275867	0,274274	1,938049
SRR1974644	endothelial	4027711	3747566	3477176	3477176	2785414	80,10563745	0	0,147462	8491201	0,000001	0,194719	0,359552	0,354663	0,091066	0,554271	0,548445	1,293915
SRR1974665	endothelial	2428798	2062216	1918412	1918412	1465715	76,40251416	0	0,074166	9386471	0,000003	0,206584	0,251264	0,397369	0,144781	0,457848	0,455798	1,202119
SRR1974667	endothelial	2096406	1719649	1571751	1571751	1276656	81,22507955	0	0,053408	11522402	0,000002	0,1493	0,18453	0,53767	0,128497	0,333831	0,331999	1,463296
SRR1974676	endothelial	2358499	1977180	1813078	1813078	1461602	80,61440269	0	0,062592	11183244	0	0,131906	0,183081	0,553545	0,131468	0,314987	0,313606	1,599111
SRR1974755	endothelial	1802286	1675658	1534957	1534957	1312289	85,493535	0	0,085976	7187738	0	0,206342	0,190629	0,429398	0,173631	0,396971	0,393194	1,323879
SRR1974757	endothelial	1432649	1336165	1232702	1232702	1035339	83,9893989	0	0,094463	5129303	0	0,257509	0,376154	0,286888	0,079448	0,633663	0,628072	1,28633
SRR1974758	endothelial	1291799	1175909	1083270	1083270	906872	83,71615571	0	0,069732	6196611	0	0,157281	0,215393	0,511663	0,115663	0,372674	0,369007	1,502265
SRR1974761	endothelial	1554376	1443347	1316835	1316835	1114684	84,64872213	0	0,10015	5186934	0	0,170997	0,238087	0,421464	0,169452	0,409084	0,405421	1,592107
SRR1974764	endothelial	2075551	1952090	1822578	1822578	1567186	85,98732126	0	0,076906	9659590	0,000004	0,195296	0,262653	0,452754	0,089292	0,457949	0,453997	1,125008
SRR1974766	endothelial	1999522	1879775	1724174	1724174	1505086	87,29316183	0	0,07268	9846143	0,000002	0,222885	0,252742	0,434669	0,089702	0,475626	0,471674	1,117939
SRR1974768	endothelial	1555527	1450231	1334148	1334148	1110064	83,20396238	0	0,087297	5982319	0	0,327841	0,338383	0,206857	0,126919	0,666224	0,659691	1,0456
SRR1974770	endothelial	1491551	1400360	1264550	1264550	1000594	79,12648768	0	0,081953	5766360	0,00011	0,312455	0,340784	0,24675	0,099901	0,653239	0,647631	1,164329
SRR1974771	endothelial	1178365	1101047	1010335	1010335	881406	87,23898509	0	0,046801	9120304	0	0,149704	0,208183	0,523267	0,118846	0,357887	0,354552	1,107417
SRR1974772	endothelial	1686865	1601088	1496437	1496437	1340479	89,57804438	0	0,042594	15285289	0,000011	0,171389	0,219793	0,475787	0,133019	0,391183	0,387873	0,796638
SRR1974774	endothelial	1744644	1622193	1504381	1504381	1259306	83,70924653	0	0,098581	5960091	0	0,210315	0,284236	0,384471	0,120977	0,494551	0,49025	1,504602

SRR1974781	endothelial	1286323	1198909	1123299	1123299	978607	87,11901284	0	0,075813	6123596	0,000009	0,211096	0,329295	0,333779	0,125821	0,540391	0,535482	1,347518
SRR1974784	endothelial	1420241	1327961	1221002	1221002	1079544	88,41459719	0	0,064441	8012355	0,000001	0,18416	0,283899	0,423703	0,108237	0,468059	0,463734	1,196209
SRR1974789	endothelial	1543335	1447582	1350221	1350221	915075	67,77223877	0	0,075198	5775410	0	0,142163	0,186299	0,463116	0,208422	0,328462	0,32568	1,782876
SRR1974794	endothelial	1259609	1173084	1082271	1082271	656630	60,6714954	0	0,059426	5303639	0,000006	0,126681	0,135688	0,596264	0,141361	0,262369	0,260174	2,002586
SRR1974798	endothelial	1879806	1772664	1672168	1672168	1046837	62,60357811	0	0,0684	7299204	0,000026	0,156873	0,194926	0,498509	0,149666	0,351799	0,348913	1,731965
SRR1974675	fetal_quiescent	2153999	1895101	1779749	1779749	1448026	81,36124813	0	0,060572	11465196	0	0,084532	0,126472	0,614495	0,174501	0,211004	0,210147	1,739087
SRR1974893	fetal_quiescent	2920387	2508253	2336964	2336964	2040726	87,32380987	0	0,096762	9853300	0	0,095036	0,169016	0,640518	0,095431	0,264051	0,263038	1,566341
SRR1974895	fetal_quiescent	2997868	2623222	2433794	2433794	1497079	61,51214934	0	0,21584	2948625	0	0,095814	0,240098	0,553473	0,110615	0,335912	0,334634	2,395314
SRR1974897	fetal_quiescent	2962015	2551671	2377042	2377042	1962617	82,5655163	0	0,095055	9658430	0,000001	0,074044	0,126592	0,706718	0,092645	0,200637	0,199902	2,052896
SRR1974898	fetal_quiescent	2566001	2226240	2069174	2069174	1676012	80,99908466	0	0,137858	5506206	0	0,09072	0,219202	0,603867	0,086211	0,309922	0,30886	1,850442
SRR1974899	fetal_quiescent	2774219	2366981	2214661	2214661	1505236	67,96688071	0	0,172917	3834764	0	0,204905	0,391802	0,313388	0,089905	0,596707	0,594285	1,832359
SRR1974901	fetal_quiescent	2013009	1744472	1649674	1649674	1359817	82,42943757	0	0,056122	11657289	0	0,223714	0,299636	0,3538	0,12285	0,523349	0,521386	0,973056
SRR1974902	fetal_quiescent	1659842	1471238	1401512	1401512	1181177	84,27876465	0	0,039483	14561483	0	0,1253	0,176284	0,597787	0,10063	0,301583	0,30068	1,152132
SRR1974903	fetal_quiescent	2391240	2112188	2004850	2004850	1644051	82,00369105	0	0,109092	6976543	0	0,237648	0,384586	0,269198	0,108568	0,622233	0,620122	0,944165
SRR1974904	fetal_quiescent	1190122	1034938	976871	976871	688042	70,43325065	0	0,058286	5670676	0	0,246172	0,340575	0,330263	0,082989	0,586747	0,584427	1,175544
SRR1974905	fetal_quiescent	1701586	1495602	1415078	1415078	1259560	89,00993443	0	0,036635	16768189	0,000001	0,12476	0,185459	0,574057	0,115722	0,310219	0,309136	0,972149
SRR1974906	fetal_quiescent	2259657	1992262	1888523	1888523	1523018	80,64598631	0	0,099581	7130586	0,000001	0,135486	0,22731	0,533495	0,103708	0,362796	0,361462	1,285651
SRR1974907	fetal_quiescent	2124248	1855627	1756839	1756839	1416888	80,64984896	0	0,068394	9880459	0	0,254983	0,322542	0,304661	0,117815	0,577524	0,575363	1,013129
SRR1974908	fetal_quiescent	2026729	1804528	1708835	1708835	1252666	73,30526353	0	0,128211	4458014	0,000083	0,147741	0,253702	0,511095	0,08738	0,401442	0,399959	1,728357
SRR1974909	fetal_quiescent	2105714	1851231	1754605	1754605	1476876	84,1714232	0	0,061889	11434025	0	0,228914	0,289695	0,383872	0,09752	0,518609	0,51663	0,843339
SRR1974910	fetal_quiescent	1680639	1477816	1401042	1401042	1216272	86,81195853	0	0,041982	14077461	0	0,18651	0,29479	0,411629	0,107071	0,4813	0,479529	0,920967
SRR1974911	fetal_quiescent	1947298	1714262	1628877	1628877	1460691	89,67472682	0	0,03315	21541860	0,000001	0,104006	0,147468	0,652582	0,095943	0,251475	0,250599	1,038702
SRR1974912	fetal_quiescent	1707664	1539230	1462926	1462926	1255287	85,80659582	0	0,059138	10190535	0,000001	0,234094	0,297677	0,375932	0,092297	0,531771	0,529866	0,953171
SRR1974913	fetal_quiescent	2333153	2092158	1987772	1987772	1736696	87,36897391	0	0,056541	14773247	0,000002	0,116774	0,153795	0,632588	0,096841	0,270569	0,269721	1,022342
SRR1974915	fetal_quiescent	1402067	1213636	1144532	1144532	914665	79,9160705	0	0,052734	8364784	0,000002	0,223365	0,337385	0,345763	0,093485	0,56075	0,558659	0,987969
SRR1974916	fetal_quiescent	2077658	1816188	1723230	1723230	1548918	89,88457722	0	0,037418	20177697	0	0,131386	0,168275	0,613056	0,087284	0,299661	0,298729	0,904517
SRR1974917	fetal_quiescent	1672990	1491111	1410788	1410788	1041712	73,83901763	0	0,059468	8407733	0,000001	0,065867	0,151979	0,600576	0,181578	0,217845	0,216944	2,31093
SRR1974918	fetal_quiescent	2128267	1876398	1780745	1780745	1534646	86,1799977	0	0,05393	13711752	0,000001	0,170251	0,213695	0,519112	0,096941	0,383947	0,382492	0,986139
SRR1974919	fetal_quiescent	1837619	1618036	1535813	1535813	1314129	85,56569061	0	0,04795	13261587	0,000003	0,166497	0,255773	0,466209	0,111518	0,42227	0,420864	0,988063
SRR1974920	fetal_quiescent	2206970	1979680	1864102	1864102	1546124	82,94202785	0	0,131691	5342731	0,000003	0,144881	0,306225	0,43974	0,109151	0,451105	0,449382	1,058071
SRR1974921	fetal_quiescent	2633138	2350196	2229784	2229784	1968830	88,29689333	0	0,080599	11548223	0	0,122416	0,182784	0,587622	0,107177	0,3052	0,304182	0,982502
SRR1974922	fetal_quiescent	1666930	1456795	1381671	1381671	1195459	86,52269607	0	0,047333	12226389	0	0,133003	0,214991	0,543179	0,108827	0,347994	0,346834	1,012355
SRR1974923	fetal_quiescent	1963600	1745042	1654915	1654915	1468196	88,71730572	0	0,052528	13481630	0	0,103251	0,178387	0,598961	0,119401	0,281638	0,280657	1,099624
SRR1974924	fetal_quiescent	2264283	2008029	1908329	1908329	1719361	90,09772424	0	0,056037	14762635	0,000002	0,114451	0,203166	0,592502	0,089878	0,317617	0,316619	0,907113
SRR1974925	fetal_quiescent	1845084	1649849	1567416	1567416	1384725	88,34444717	0	0,070533	9348915	0,000024	0,189771	0,31848	0,407596	0,084129	0,508251	0,506389	0,827152
SRR1974926	fetal_quiescent	2341295	2082531	1975359	1975359	1810659	91,66227506	0	0,059304	14656240	0,000008	0,139378	0,227628	0,543822	0,089164	0,367005	0,365703	0,786871
SRR1974927	fetal_quiescent	3101561	2786583	2645130	2645130	2306552	87,19994858	0	0,101818	10544156	0	0,133228	0,223175	0,546457	0,09714	0,356403	0,355233	1,089266
SRR1974928	fetal_quiescent	1822318	1571131	1484576	1484576	1021481	68,80624502	0	0,064531	7570388	0	0,20008	0,264848	0,408135	0,126936	0,464929	0,462856	1,571258
SRR1974929	fetal_quiescent	2622119	2358889	2246599	2246599	2012012	89,55812764	0	0,090121	10481548	0,000003	0,110792	0,199834	0,586413	0,102958	0,310626	0,309685	1,000193
SRR1974930	fetal_quiescent	2265682	2026279	1925615	1925615	1727922	89,73351371	0	0,040537	20733183	0	0,119413	0,172114	0,610255	0,098218	0,291527	0,290604	0,905713
SRR1974931	fetal_quiescent	2477946	2243947	2137334	2137334	1905794	89,16687799	0	0,07286	12435091	0	0,101386	0,163218	0,636428	0,098969	0,264603	0,263736	1,095827
SRR1974932	fetal_quiescent	1843018	1654885	1564207	1564207	1254393	80,19354216	0	0,138385	4103702	0,000002	0,147888	0,297725	0,467352	0,087032	0,445613	0,44391	1,353582
SRR1974933	fetal_quiescent	1997141	1767903	1676263	1676263	1359710	81,11555287	0	0,103127	6130909	0	0,15668	0,279238	0,459389	0,104694	0,435918	0,434489	1,246637
SRR1974934	fetal_quiescent	1009804	874124	824506	824506	680960	82,59005999	0	0,033326	9988230	0	0,088876	0,14234	0,654218	0,114567	0,231215	0,230428	1,695292
SRR1974935	fetal_quiescent	2241490	1980374	1878660	1878660	1674595	89,13773647	0	0,044396	18297402	0,000001	0,125818	0,182333	0,595334	0,096514	0,308151	0,307002	0,956437
SRR1974936	fetal_quiescent	1947320	1728422	1626760	1626760	1409505	86,64492611	0	0,055226	12286841	0,000002	0,126295	0,200078	0,575681	0,097943	0,326374	0,325242	0,948744
SRR1974937	fetal_quiescent	2358703	2084164	1977870	1977870	1748388	88,39751854	0	0,044094	19238615	0,000014	0,121823	0,168835	0,6113	0,098027	0,290659	0,28963	1,03089
SRR1974938	fetal_quiescent	1812973	1632259	1544875	1544875	1211032	78,39029048	0	0,137086	4003396	0	0,190071	0,364918	0,337972	0,107039	0,554989	0,552911	1,239729
SRR1974939	fetal_quiescent	1741301	1586847	1510618	1510618	1319115	87,32287051	0	0,040182	15971438	0,000003	0,089026	0,142855	0,633705	0,134411	0,231881	0,23111	1,33533
SRR1974940	fetal_quiescent	2025248	1793263	1704404	1704404	1517360	89,02584129	0	0,038646	19122378	0,000001	0,124308	0,163694	0,615992	0,096005	0,288002	0,287009	0,993497
SRR1974941	fetal_quiescent	2136650	1923448	1814878	1814878	1580948	87,11042836	0	0,143262	4977070	0,000001	0,136365	0,277535	0,494754	0,091346	0,4139	0,412492	1,06645
SRR1974942	fetal_quiescent	2558291	2318689	2213244	2213244	1991097	89,96283284	0	0,05678	16863179	0	0,109173	0,165056	0,626548	0,099222	0,27423	0,273379	0,975105
SRR1974943	fetal_quiescent	1389648	1232890	1165663	1165663	898561	77,08583012	0	0,070403	6078419	0,000003	0,17375	0,262249	0,441176	0,122821	0,436	0,434263	1,184165
SRR1974944	fetal_quiescent	2253384	1998410	1896787	1896787	1729577	91,18456632	0	0,070244	11727708	0,000001	0,169136	0,271049	0,449525	0,110289	0,440185	0,438755	0,784893
SRR1974945	fetal_quiescent	2589261	2314791	2199880	2199880	1942377	88,29467971	0	0,065229	14234071	0	0,111967	0,17537	0,613501	0,099162	0,287337	0,286463	1,04329
SRR1974947	fetal_quiescent	2024105	1788421	1698966	1698966	1269050	74,6954324	0	0,12884	4492105	0	0,175872	0,334178	0,387325	0,102626	0,510049	0,508144	1,336256
SRR1974948	fetal_quiescent	2373120	2132876	2023955	2023955	1440107	71,15311358	0	0,131481	4985110	0	0,100922	0,18805	0,582506	0,128522	0,288972	0,287677	1,953893
SRR1974949	fetal_quiescent	2288672	2073762	1977914	1977914	1516381	76,66566898	0	0,162481	4145854	0	0,208124	0,379944	0,307159	0,104773	0,588068	0,585862	1,285184
SRR1974950	fetal_quiescent	2064135	1799916	1709059	1709059	1248854	73,07260896	0	0,12431	4597611	0,000004	0,201022	0,354146	0,363325	0,081504	0,555167	0,552873	1,385331
SRR1974951	fetal_quiescent	2222080	1985194	1887902	1887902	1508715	79,91490024	0	0,108508	6439508	0	0,118062	0,194068	0,605353	0,082517	0,31213	0,311166	1,363281
SRR1974952	fetal_quiescent	2626592	2401082	2280617	2280617	1794241	78,67349055	0	0,109422	7589023	0	0,151046	0,216304	0,474886	0,157764	0,36735	0,366068	1,227393
SRR1974953	fetal_quiescent	1660401	1452624	1375175	1375175	1055644	76,76433908	0	0,055859	9093960	0	0,120979	0,187011	0,572141	0,119869	0,30799	0,306555	1,655871
SRR1974954	fetal_quiescent	1905241	1719054	1641145	1641145	1396060	85,06621901	0	0,054548	12326954	0	0,109347	0,1763	0,573449	0,140904	0,285647	0,284703	1,185843
SRR1974955	fetal_quiescent	2098591	1892069	1801275	1801275	1594689	88,53112379	0	0,11553	6359156	0	0,1117	0,221642	0,568587	0,098072	0,333341	0,331951	1,034919
SRR1974956	fetal_quiescent	2544829	2288298	2172127	2172127	1840198	84,7187112	0	0,131793	6353460	0	0,089632	0,169918	0,597511	0,142939	0,25955	0,258607	1,384102
SRR1974957	fetal_quiescent	2146029	1907340	1804529	1804529	1337154	74,09988978	0	0,074739	8494036	0	0,160151	0,213701	0,531349	0,094799	0,373851	0,372203	1,365399
SRR1974958	fetal_quiescent	1429868	1258219	1189014	1189014	852419	71,69125006	0	0,087734	4569481	0	0,101095	0,217683	0,54282	0,138402	0,318777	0,317537	1,735188
SRR1974959	fetal_quiescent	2099936	1866385	1771156	1771156	1403053	79,216794	0	0,10443	6241352	0,000001	0,252649	0,335919	0,309923	0,101508	0,588568	0,586156	1,075831
SRR1974960	fetal_quiescent	1252637	1091170	1025284	1025284	851132	83,01426727	0	0,037119	11179469	0,000001	0,136364	0,204587	0,53966	0,119386	0,340952	0,33949	1,160749
SRR1974961	fetal_quiescent	2277496	2040278	1937394	1937394	1500528	77,45084376	0	0,114164	6061499	0	0,118872	0,208616	0,560168	0,112344	0,327488	0,326435	1,323258
SRR1974962	fetal_quiescent	2437317	2205603	2102197	2102197	1749309	83,21337153	0	0,170715	4522064	0	0,06484	0,140635	0,674295	0,120231	0,205474	0,204779	1,664206
SRR1974963	fetal_quiescent	1790829	1590533	1511343	1511343	1315624	87,04999461	0	0,067398	9316505	0	0,098905	0,174022	0,62994	0,097133	0,272927	0,27203	1,201818
SRR1974964	fetal_quiescent	1825954	1612735	1526879	1526879	1191502	78,03512918	0	0,055395	10353693	0,000001	0,159897	0,220205	0,487034	0,132864	0,380102	0,378536	1,157621
SRR1974965	fetal_quiescent	1963866	1725315	1632247	1632247	1401876	85,88626599	0	0,061595	10907474	0,000001	0,211139	0,335952	0,348403	0,104505	0,547091	0,544936	0,905182
SRR1974966	fetal_quiescent	2377029	2152243	2047739	2047739	1751435	85,5301872	0	0,109285	7418063	0,000002	0,073482	0,140401	0,648764	0,137351	0,213883	0,213218	1,562676
SRR1974967	fetal_quiescent	1119394	993199	942341	942341	807921	85,73552461	0	0,027661	14333342	0	0,118651	0,14679	0,579399	0,15516	0,265441	0,264438	1,179296
SRR1974968	fetal_quiescent	2152157	1924423	1829281	1829281	1360366	74,36615807	0	0,09821	6464536	0	0,1331	0,251388	0,516787	0,098725	0,384488	0,383032	1,587828
SRR1974969	fetal_quiescent	2112415	1863659	1756446	1756446	1343080	76,46577236	0	0,071639	8920682	0,000005	0,174615	0,261778	0,385999	0,177603	0,436393	0,434271	1,357303

SRR1974970	fetal_quiescent	2086716	1853093	1762079	1762079	1420302	80,60376408	0	0,085927	7783993	0	0,186482	0,29662	0,406603	0,110295	0,483101	0,481189	1,193353
SRR1974971	fetal_quiescent	1752345	1559127	1482748	1482748	1160761	78,28444213	0	0,088818	6141521	0	0,118204	0,180258	0,507993	0,193545	0,298462	0,297292	1,675564
SRR1974972	fetal_quiescent	1916912	1702363	1612862	1612862	1330946	82,52076123	0	0,060693	10516302	0	0,139041	0,213119	0,505856	0,141984	0,35216	0,350901	1,241444
SRR1974973	fetal_quiescent	1839044	1642472	1557217	1557217	1243465	79,85174834	0	0,060616	9838070	0	0,086442	0,119237	0,662681	0,13164	0,205679	0,204898	1,680384
SRR1974974	fetal_quiescent	2364699	2122784	2017287	2017287	1486076	73,66705878	0	0,144203	4644458	0	0,108413	0,225399	0,575275	0,090913	0,333812	0,332626	1,606735
SRR1974975	fetal_quiescent	1366754	1192814	1131654	1131654	880595	77,81486214	0	0,049197	8653602	0,000001	0,138209	0,222602	0,47065	0,168537	0,360812	0,359289	1,435568
SRR1974976	fetal_quiescent	1492757	1336689	1263436	1263436	882736	69,86788409	0	0,113987	3571903	0	0,104926	0,225469	0,558508	0,111097	0,330395	0,329171	1,794819
SRR1974977	fetal_quiescent	1855213	1635196	1553242	1553242	1383020	89,04085777	0	0,052053	12819670	0	0,097214	0,161276	0,640758	0,100752	0,25849	0,25759	1,105795
SRR1974978	fetal_quiescent	1746381	1547938	1468971	1468971	1253156	85,30842338	0	0,078036	7605978	0	0,210399	0,330917	0,360222	0,098462	0,541316	0,539171	0,944029
SRR1974979	fetal_quiescent	2163371	1913589	1819620	1819620	1266175	69,58458359	0	0,139917	4092009	0	0,22827	0,395506	0,268615	0,107609	0,623777	0,621449	1,267185
SRR1974980	fetal_quiescent	957772	885063	821926	821926	715009	86,99189465	0	0,046243	7490814	0,000001	0,103959	0,19075	0,599277	0,106012	0,29471	0,292332	1,232879
SRR1974981	fetal_quiescent	2412694	2267744	2127565	2127565	1892124	88,93378111	0	0,05415	16834482	0,000004	0,142655	0,196238	0,5662	0,094902	0,338893	0,336234	0,834432
SRR1974982	fetal_quiescent	1613127	1499816	1405423	1405423	1238681	88,13581392	0	0,076401	7688022	0,000001	0,167658	0,28194	0,437999	0,112402	0,449598	0,445998	1,007752
SRR1974983	fetal_quiescent	1656961	1509694	1405614	1405614	1185083	84,31069981	0	0,082728	6761764	0	0,114041	0,191521	0,570949	0,12349	0,305562	0,303333	1,772615
SRR1974984	fetal_quiescent	1718082	1597979	1490651	1490651	1226257	82,26318568	0	0,091037	6319647	0,000002	0,176132	0,263201	0,468297	0,092369	0,439333	0,435652	1,537322
SRR1974985	fetal_quiescent	1436390	1363423	1279273	1279273	1139943	89,10865781	0	0,059857	9138261	0,000028	0,16479	0,252133	0,470973	0,112077	0,416922	0,413976	1,091206
SRR1974986	fetal_quiescent	1110796	1051040	1006446	1006446	849502	84,40611816	0	0,057892	7051022	0	0,235296	0,186361	0,485062	0,093281	0,421657	0,418152	1,092971
SRR1974987	fetal_quiescent	1574514	1469135	1374902	1374902	1085006	78,91515177	0	0,094813	5354110	0,000001	0,1805	0,270508	0,44295	0,106042	0,451007	0,447305	1,61488
SRR1974988	fetal_quiescent	1302901	1241432	1164744	1164744	1011414	86,83573386	0	0,063418	7633341	0	0,226962	0,249072	0,412947	0,111019	0,476034	0,472369	1,152569
SRR1974990	fetal_quiescent	1558646	1477958	1386354	1386354	1249098	90,0994984	0	0,055588	10814955	0,000009	0,145577	0,225377	0,52882	0,100217	0,370954	0,368164	0,877599
SRR1974991	fetal_quiescent	1811917	1712982	1610258	1610258	1408536	87,47269071	0	0,067407	9973030	0	0,15993	0,246719	0,505607	0,087744	0,406649	0,403582	1,249721
SRR1974992	fetal_quiescent	1654538	1561585	1465963	1465963	1281699	87,43051496	0	0,061382	10008628	0	0,116633	0,181682	0,632832	0,068854	0,298315	0,296181	1,301183
SRR1974993	fetal_quiescent	1824216	1672467	1569328	1569328	1325333	84,45226237	0	0,085937	7262669	0	0,21615	0,269444	0,423236	0,09117	0,485594	0,481743	1,306953
SRR1974994	fetal_quiescent	1747686	1658960	1563386	1563386	1391260	88,99017901	0	0,066489	9993190	0	0,2117	0,29862	0,376227	0,113453	0,51032	0,506232	0,711142
SRR1974995	fetal_quiescent	1750524	1653131	1552331	1552331	1353262	87,17612416	0	0,086088	7401859	0	0,134055	0,231933	0,519072	0,11494	0,365988	0,363171	1,395767
SRR1974996	fetal_quiescent	1954519	1814233	1696976	1696976	1489421	87,76912579	0	0,061004	11705827	0,000001	0,113006	0,170274	0,606894	0,109824	0,28328	0,281096	1,107112
SRR1974998	fetal_quiescent	1880853	1785414	1682065	1682065	1491510	88,67136526	0	0,057762	12408654	0	0,106444	0,175745	0,613048	0,104763	0,282189	0,280084	1,237569
SRR1974999	fetal_quiescent	2054215	1949592	1836313	1836313	1634674	89,01935563	0	0,070403	11057864	0,000005	0,100463	0,145408	0,673178	0,080946	0,245871	0,244157	1,562221
SRR1975000	fetal_quiescent	1720115	1618470	1515606	1515606	1348581	88,97965566	0	0,06084	10628863	0	0,126552	0,206465	0,570208	0,096775	0,333017	0,33053	1,121473
SRR1975001	fetal_quiescent	1313460	1209015	1131419	1131419	878394	77,63649011	0	0,095799	4286854	0	0,196968	0,296599	0,377271	0,129162	0,493566	0,489729	1,864598
SRR1975002	fetal_quiescent	1766481	1668008	1575212	1575212	1363204	86,54098623	0	0,074406	8700357	0	0,114745	0,154864	0,610824	0,119568	0,269609	0,267619	1,789877
SRR1975003	fetal_quiescent	1341504	1268295	1196251	1196251	1023376	85,54860142	0	0,080841	5983609	0	0,145087	0,231611	0,510523	0,112779	0,376698	0,373896	1,527368
SRR1975004	fetal_quiescent	1349065	1279378	1207744	1207744	1055675	87,4088383	0	0,066538	7576877	0	0,200631	0,299741	0,402034	0,097594	0,500372	0,496757	1,152021
SRR1975005	fetal_quiescent	1769628	1604423	1503837	1503837	1279895	85,10862547	0	0,0702	8684305	0,000006	0,147287	0,231718	0,524511	0,096478	0,379005	0,376082	1,245493
SRR1975006	fetal_quiescent	1667634	1562679	1464404	1464404	1133592	77,40978582	0	0,119866	4342647	0	0,145028	0,249314	0,462129	0,143528	0,394342	0,391281	2,154618
SRR1975007	fetal_quiescent	2200461	2101091	1991849	1991849	1773147	89,02015163	0	0,069495	12159194	0	0,169422	0,20344	0,537416	0,089723	0,372861	0,370185	0,974563
SRR1975008	fetal_quiescent	1663284	1578809	1486232	1486232	1275157	85,7979777	0	0,082471	7299809	0,000001	0,133612	0,19894	0,572933	0,094514	0,332553	0,330226	1,686636
SRR1974875	fetal_replicating	2681531	2310602	2165526	2165526	1953500	90,20903005	0	0,076853	12049488	0	0,16253	0,210439	0,544983	0,082048	0,372969	0,371635	0,826235
SRR1974876	fetal_replicating	2028142	1730782	1614984	1614984	1330135	82,36211628	0	0,109807	5604694	0	0,245627	0,386428	0,271964	0,095981	0,632055	0,629243	1,063749
SRR1974877	fetal_replicating	2679417	2290410	2128466	2128466	1656240	77,81378702	0	0,133824	5622758	0,000001	0,077885	0,14243	0,691872	0,087812	0,220315	0,219427	2,088715
SRR1974878	fetal_replicating	2105364	1784281	1665678	1665678	1457820	87,52111753	0	0,058859	11893114	0	0,187734	0,225401	0,511983	0,074882	0,413135	0,411472	1,013735
SRR1974879	fetal_replicating	3172954	2760855	2572313	2572313	2169027	84,3220479	0	0,157037	6162406	0	0,112525	0,244609	0,559266	0,0836	0,357134	0,355823	1,497425
SRR1974880	fetal_replicating	2972100	2604611	2428467	2428467	2068355	85,17122119	0	0,171203	5329487	0	0,094885	0,172842	0,666867	0,065406	0,267727	0,266728	1,643894
SRR1974881	fetal_replicating	2169683	1874361	1749428	1749428	1483066	84,77433767	0	0,125597	5398625	0,000005	0,118758	0,216369	0,560769	0,104099	0,335128	0,333921	1,417202
SRR1974882	fetal_replicating	1836518	1615864	1507021	1507021	1340544	88,95323954	0	0,054854	11768176	0,000003	0,130261	0,191346	0,561118	0,117272	0,321607	0,320488	1,242917
SRR1974883	fetal_replicating	2385711	2072462	1936106	1936106	1708377	88,23778244	0	0,084186	9568644	0,000001	0,117773	0,205242	0,556412	0,120573	0,323015	0,321834	1,226528
SRR1974884	fetal_replicating	3386354	2961395	2784213	2784213	2006709	72,07455033	0	0,21583	3952609	0	0,160927	0,365962	0,383515	0,089596	0,526889	0,524821	1,875185
SRR1974885	fetal_replicating	2598586	2265861	2126996	2126996	1895530	89,11770403	0	0,103945	8474498	0	0,140119	0,245411	0,511664	0,102806	0,38553	0,38406	0,975172
SRR1974886	fetal_replicating	2493768	2137415	1986472	1986472	1708858	86,02477155	0	0,097182	8212652	0,000001	0,14582	0,2385	0,513282	0,102397	0,38432	0,382872	1,288566
SRR1974887	fetal_replicating	3575583	3154429	2940657	2940657	2613148	88,86272693	0	0,162685	7134323	0,000031	0,283355	0,399501	0,240706	0,076407	0,682856	0,680205	0,810243
SRR1974888	fetal_replicating	2779367	2413112	2257851	2257851	1867995	82,73331588	0	0,126241	6761783	0	0,117131	0,21194	0,577167	0,093761	0,329071	0,327748	1,58831
SRR1974889	fetal_replicating	2654964	2265276	2119276	2119276	1417943	66,90695313	0	0,139894	4583336	0	0,135413	0,211971	0,578293	0,074323	0,347384	0,345929	1,891912
SRR1974890	fetal_replicating	2636438	2279903	2118938	2118938	1866873	88,10418238	0	0,099242	8772452	0	0,162395	0,244882	0,501594	0,091129	0,407277	0,40563	0,989507
SRR1974891	fetal_replicating	2667165	2318520	2159456	2159456	1901405	88,05018486	0	0,130167	6655121	0	0,103191	0,186606	0,636481	0,073722	0,289797	0,288784	1,378295
SRR1974892	fetal_replicating	1719377	1466482	1356970	1356970	1201153	88,51728483	0	0,049607	11702821	0	0,158837	0,233296	0,503385	0,104482	0,392133	0,390521	1,130091
SRR1974894	fetal_replicating	1870820	1611558	1495271	1495271	1314056	87,88079218	0	0,074622	8361008	0	0,126151	0,22254	0,568213	0,083095	0,348691	0,347328	1,280628
SRR1974896	fetal_replicating	2445809	2123821	1977682	1977682	1614127	81,61711539	0	0,179334	3944460	0	0,21469	0,388244	0,331155	0,065912	0,602933	0,600671	1,456951
SRR1974900	fetal_replicating	2698557	2327646	2167126	2167126	1841750	84,98582916	0	0,129756	6468747	0,000001	0,113138	0,193313	0,614616	0,078932	0,30645	0,305396	1,532018
SRR1974914	fetal_replicating	1453349	1251561	1187400	1187400	984399	82,90373926	0	0,042129	11352589	0	0,227049	0,254147	0,441999	0,076805	0,481196	0,479428	0,95679
SRR1974946	fetal_replicating	1247920	1072377	1010247	1010247	661935	65,52209509	0	0,053903	5917419	0	0,090667	0,163981	0,626107	0,119245	0,254647	0,253561	2,017786
SRR1974989	fetal_replicating	2008119	1864676	1751819	1751819	1437594	82,06293002	0	0,087915	7689480	0	0,279878	0,302842	0,319485	0,097796	0,58272	0,577905	1,150556
SRR1974997	fetal_replicating	1569817	1484690	1399231	1399231	1235949	88,33059016	0	0,055472	10724316	0,000001	0,164781	0,191062	0,547992	0,096164	0,355843	0,35322	1,028992
SRR1974544	hybrid	2752725	2639680	2447300	2447300	2191073	89,53021697	0	0,249571	3624065	0	0,125928	0,276236	0,419808	0,178028	0,402163	0,399461	1,275568
SRR1974546	hybrid	2502199	2405769	2213973	2213973	1984558	89,63785918	0	0,166916	5263002	0,000001	0,062775	0,14756	0,655346	0,134318	0,210336	0,208969	1,770095
SRR1974547	hybrid	2074547	1974775	1802163	1802163	1568604	87,04007351	0	0,229433	2872680	0	0,086821	0,168438	0,561453	0,183288	0,255259	0,253185	1,657686
SRR1974550	hybrid	2606101	2494837	2272309	2272309	2035235	89,56682388	0	0,202338	4325148	0	0,032626	0,088264	0,714532	0,164577	0,12089	0,119901	2,204837
SRR1974551	hybrid	1939720	1851364	1710100	1710100	1510766	88,34372259	0	0,191212	3428977	0,000013	0,102345	0,180158	0,590404	0,12708	0,282503	0,280571	1,39499
SRR1974552	hybrid	2989537	2881111	2715974	2715974	2331876	85,85781749	0	0,283573	3290673	0	0,159469	0,441898	0,331884	0,066749	0,601368	0,597281	1,498953
SRR1974554	hybrid	2828500	2702666	2499116	2499116	2246933	89,90911186	0	0,091219	11555129	0,000002	0,061615	0,145744	0,640403	0,152236	0,207359	0,205841	1,53573
SRR1974555	hybrid	3449667	3306565	3064054	3064054	2807378	91,62299359	0	0,147765	8538617	0,000001	0,094702	0,201019	0,579474	0,124804	0,295721	0,293782	0,987056
SRR1974556	hybrid	1612687	1499712	1363613	1363613	1219972	89,46614619	0	0,085762	6699808	0,000001	0,080577	0,184029	0,572895	0,162498	0,264606	0,262082	1,280921
SRR1974558	hybrid	4111920	3991197	3730747	3730747	3404060	91,24338906	0	0,206708	7055185	0	0,07139	0,149313	0,649471	0,129825	0,220703	0,219431	1,338114
SRR1974559	hybrid	4006011	3880537	3597477	3597477	3320865	92,31094459	0	0,205593	6926586	0,000001	0,057901	0,171117	0,637116	0,133866	0,229017	0,227604	1,33323
SRR1974560	hybrid	2748068	2632452	2446670	2446670	2202628	90,02554492	0	0,103647	9877967	0	0,068638	0,15831	0,624337	0,148715	0,226948	0,225336	1,474526
SRR1974567	hybrid	3374680	3268014	3078216	3078216	2784851	90,46964216	0	0,202594	5909420	0,000001	0,154147	0,371952	0,381125	0,092775	0,526099	0,522922	1,132749
SRR1974568	hybrid	2779937	2673802	2486451	2486451	2245999	90,32950981	0	0,121011	8515337	0,000001	0,052896	0,107201	0,733107	0,106795	0,160097	0,159035	1,498594
SRR1974569	hybrid	2962471	2859740	2680450	2680450	2422665	90,38277155	0	0,184655	5724789	0,000039	0,102181	0,197038	0,617332	0,083411	0,29922	0,297457	1,512637
SRR1974577	hybrid	1871360	1765679	1636662	1636662	1272139	77,72765544	0	0,138989	4141737	0,000001	0,144465	0,239743	0,450939	0,164852	0,384208	0,381333	1,572464

SRR1974584	hybrid	2291202	2212321	2084261	2084261	1881159	90,25544306	0	0,128927	6653876	0	0,17397	0,20418	0,483369	0,138481	0,37815	0,375813	1,086566
SRR1974601	hybrid	2459644	2315885	2122929	2122929	1746115	82,2502778	0	0,0986	8262393	0,000001	0,081851	0,133321	0,619383	0,165445	0,215172	0,212995	1,545666
SRR1974608	hybrid	3194361	3017306	2819228	2819228	2476932	87,85852013	0	0,081085	14436365	0,000002	0,065676	0,120588	0,674898	0,138836	0,186264	0,184549	1,160069
SRR1974619	hybrid	2653791	2507500	2304582	2304582	1930689	83,77610343	0	0,094197	9593886	0	0,109129	0,152851	0,598209	0,139811	0,26198	0,259462	1,374865
SRR1974680	hybrid	4000103	3718709	3557025	3557025	2522396	70,91308045	0	0,100825	11662344	0,000015	0,198359	0,252079	0,415081	0,134466	0,450438	0,444991	0,850234
SRR1974683	hybrid	6527969	6115482	5882396	5882396	4335068	73,69561655	0	0,105572	19070752	0,000009	0,151928	0,173414	0,554115	0,120535	0,325342	0,321296	0,778325
SRR1974685	hybrid	7618948	7179004	6935227	6935227	5064634	73,02766009	0	0,107287	21894621	0	0,126859	0,142563	0,594216	0,136362	0,269422	0,266346	0,8353
SRR1974687	hybrid	5781970	5421782	5217381	5217381	3964071	75,97817756	0	0,109971	16681168	0,00001	0,134362	0,185143	0,546884	0,133602	0,319505	0,315926	0,740417
SRR1974691	hybrid	8122463	7659787	7398401	7398401	5391788	72,8777475	0	0,123406	20017804	0	0,172427	0,204947	0,49584	0,126786	0,377374	0,373145	0,753785
SRR1974694	hybrid	3375367	3169726	3050337	3050337	2229870	73,10241459	0	0,085212	12336718	0	0,146977	0,184992	0,550946	0,117086	0,331968	0,327866	0,834169
SRR1974695	hybrid	4480658	4232368	4010590	4010590	2249527	56,08967758	0	0,112194	9265925	0	0,058312	0,077311	0,641794	0,222582	0,135623	0,133746	2,092951
SRR1974696	hybrid	5326782	5047883	4838151	4838151	3089976	63,86687807	0	0,152563	9073124	0	0,106577	0,144795	0,569838	0,178791	0,251372	0,248089	1,668272
SRR1974700	hybrid	6095305	5734243	5508806	5508806	4044747	73,42329717	0	0,109672	17072040	0	0,174331	0,221519	0,483252	0,120898	0,39585	0,391036	0,732324
SRR1974702	hybrid	5373115	5041602	4862266	4862266	2802031	57,62808945	0	0,174789	7054160	0	0,42361	0,416503	0,067551	0,092337	0,840113	0,829167	0,941944
SRR1974708	hybrid	6779970	6305840	6024455	6024455	3504450	58,17040712	0	0,155577	10067068	0,000001	0,052991	0,083006	0,638662	0,225341	0,135997	0,134233	2,534686
SRR1974716	hybrid	6067086	5687917	5437666	5437666	3507704	64,50752952	0	0,142699	11097836	0,000002	0,088789	0,153145	0,587804	0,170261	0,241933	0,239192	1,664752
SRR1974719	hybrid	5778651	5472177	5248184	5248184	3601339	68,62066955	0	0,117326	14130651	0	0,16994	0,159912	0,546263	0,123885	0,329852	0,326006	1,055839
SRR1974729	hybrid	6748865	6348745	6122532	6122532	4587611	74,92996362	0	0,106969	19893808	0	0,172935	0,217981	0,505368	0,103716	0,390916	0,386366	0,693978
SRR1974742	hybrid	6495005	6161829	5969738	5969738	4174066	69,92042197	0	0,127484	14955377	0,000007	0,244649	0,212214	0,428136	0,114993	0,456863	0,451329	0,851372
SRR1974746	hybrid	8620937	8088555	7775642	7775642	5593306	71,93368728	0	0,122586	20915169	0,000001	0,200126	0,232004	0,460081	0,107789	0,432129	0,426941	0,708837
SRR1974747	hybrid	7435686	7023210	6774821	6774821	4974565	73,42725365	0	0,111443	20636403	0,000003	0,150341	0,176525	0,539493	0,133637	0,326866	0,323217	0,79211
SRR1974748	hybrid	6376249	6003243	5784772	5784772	4106217	70,98321248	0	0,113643	16679527	0	0,125408	0,137014	0,608053	0,129525	0,262422	0,259367	1,041483
SRR1974749	hybrid	6984531	6589958	6298086	6298086	4429997	70,33878229	0	0,133188	15126244	0	0,09628	0,105272	0,664066	0,134383	0,201552	0,199181	1,30548
SRR1974804	hybrid	3811218	3676657	3437125	3437125	2966224	86,29956723	0	0,097893	14144592	0,000026	0,101822	0,175304	0,630045	0,092803	0,277125	0,275239	0,99331
SRR1974816	hybrid	487376	456414	427859	427859	333290	77,89715771	0	0,032104	5079056	0	0,259316	0,19142	0,427218	0,122045	0,450736	0,445699	1,254775
SRR1974826	hybrid	2553920	2453355	2294121	2294121	1795222	78,2531523	0	0,123395	6662673	0,000002	0,073703	0,179189	0,638301	0,108806	0,252891	0,250929	1,963689
SRR1974833	hybrid	2508730	2385787	2243431	2243431	1879132	83,7615242	0	0,063502	14162563	0,000002	0,185914	0,245039	0,483692	0,085354	0,430952	0,427348	0,836947
SRR1974837	hybrid	3590155	3429084	3171418	3171418	2736857	86,29758045	0	0,098128	13017315	0	0,093051	0,203171	0,591437	0,112341	0,296222	0,293927	0,960499
SRR1974850	hybrid	3402184	3231374	3006750	3006750	2544535	84,62742163	0	0,074895	16128222	0,000006	0,085953	0,148365	0,66288	0,102796	0,234319	0,232552	1,21946
SRR1974855	hybrid	2918196	2813247	2627146	2627146	2150126	81,84265359	0	0,101931	9817348	0,000009	0,156868	0,254711	0,467144	0,121269	0,411579	0,408683	1,032159
SRR1974582	microglia	2281856	2162965	2001564	2001564	1755866	87,72469929	0	0,106169	7672905	0	0,065324	0,115325	0,660378	0,158973	0,180649	0,179283	1,671423
SRR1974590	microglia	3273255	3140053	2928717	2928717	2386697	81,49291994	0	0,226199	4445829	0	0,112467	0,274701	0,523799	0,089034	0,387168	0,384674	1,763276
SRR1974591	microglia	1934969	1833564	1717992	1717992	1439961	83,8165137	0	0,121889	5416439	0	0,114695	0,18712	0,555444	0,142741	0,301815	0,299502	1,876523
SRR1974595	microglia	2402201	2274122	2118208	2118208	1878896	88,70214823	0	0,113897	7609239	0,000002	0,119959	0,242086	0,545084	0,092869	0,362045	0,359333	1,458646
SRR1974597	microglia	2695644	2592454	2398469	2398469	2126469	88,65943233	0	0,204862	4453916	0	0,065207	0,14705	0,686753	0,10099	0,212256	0,210889	1,939209
SRR1974598	microglia	2068392	1944906	1801814	1801814	1539580	85,44611153	0	0,135561	5152840	0,000042	0,149798	0,277167	0,463108	0,109886	0,426965	0,423691	1,610874
SRR1974599	microglia	1870663	1771047	1661224	1661224	1378692	82,99254044	0	0,149242	4146968	0,000001	0,203092	0,304433	0,392571	0,099904	0,507525	0,503708	1,685011
SRR1974606	microglia	3147676	2985096	2841064	2841064	2194602	77,24577834	0	0,125846	7971388	0,00001	0,171607	0,142378	0,578873	0,107132	0,313985	0,310579	1,45133
SRR1974666	microglia	2232870	1874450	1744457	1744457	1349355	77,35100378	0	0,080722	7901897	0	0,114468	0,158228	0,630528	0,096776	0,272696	0,271482	1,725335
SRR1974668	microglia	1694632	1409310	1303162	1303162	983270	75,45262983	0	0,084666	5474122	0	0,118614	0,192185	0,58286	0,106341	0,310799	0,309215	1,776182
SRR1974669	microglia	1076977	932045	860433	860433	655928	76,23231559	0	0,042099	7570067	0,000186	0,114537	0,172964	0,53857	0,173745	0,287501	0,285982	1,867033
SRR1974671	microglia	2243147	1922741	1788595	1788595	1258260	70,34907288	0	0,129666	4422742	0,000109	0,1349	0,201078	0,488467	0,175446	0,335979	0,334174	2,018131
SRR1974788	microglia	1700052	1562932	1476602	1476602	808371	54,74535454	0	0,077478	4943686	0,00001	0,200121	0,192129	0,499733	0,108007	0,392251	0,388651	1,831102
SRR1974860	microglia	1492728	1221535	1121279	1121279	681698	60,79646546	0	0,08605	3730414	0,000001	0,29316	0,262293	0,340452	0,104094	0,555453	0,55274	1,734899
SRR1974861	microglia	2030215	1669276	1535797	1535797	745807	48,5615612	0	0,119371	2870003	0	0,330025	0,296914	0,248162	0,124899	0,626939	0,622827	1,759477
SRR1974868	microglia	2979547	2553854	2367335	2367335	1912815	80,80035145	0	0,108337	8178227	0	0,116869	0,17452	0,607814	0,100797	0,291389	0,289998	1,467072
SRR1974602	neurons	2940987	2791936	2616933	2616933	2289470	87,48676409	0	0,080557	13436444	0,000005	0,187665	0,238624	0,443529	0,130176	0,426289	0,422115	0,647441
SRR1974603	neurons	2243421	2116527	1985709	1985709	1557857	78,45343905	0	0,100061	7256131	0	0,140447	0,19247	0,448606	0,218477	0,332916	0,329607	1,375681
SRR1974605	neurons	3344038	3174498	2975040	2975040	2578482	86,67049855	0	0,086314	14064249	0	0,074156	0,11129	0,654563	0,159992	0,185446	0,183698	1,148374
SRR1974607	neurons	3998626	3773857	3511947	3511947	2992006	85,1950784	0	0,106554	13023735	0	0,116686	0,195002	0,539542	0,148769	0,311688	0,308858	1,109974
SRR1974609	neurons	3292550	3138761	2926630	2926630	2591011	88,53223674	0	0,082355	14854573	0,000007	0,068363	0,105135	0,67554	0,150955	0,173497	0,171973	0,889892
SRR1974611	neurons	2340586	2213803	2090267	2090267	1636444	78,2887545	0	0,117192	6425039	0,00006	0,095107	0,093871	0,56623	0,244733	0,188979	0,186819	1,718953
SRR1974612	neurons	1671720	1571864	1478928	1478928	1215409	82,18175597	0	0,069319	8356776	0,000001	0,175415	0,11642	0,571749	0,136414	0,291835	0,288827	1,008936
SRR1974613	neurons	1923060	1799027	1657701	1657701	1429113	86,21054098	0	0,078151	8660399	0,000001	0,041642	0,074717	0,721436	0,162203	0,116359	0,115202	1,51555
SRR1974614	neurons	1903042	1781329	1658699	1658699	1329587	80,15842537	0	0,112076	5479613	0,000004	0,117136	0,14019	0,482798	0,259872	0,257326	0,254539	1,424662
SRR1974615	neurons	2615108	2474886	2292502	2292502	1984280	86,55521347	0	0,080566	11643951	0,000002	0,061797	0,111086	0,66882	0,158294	0,172883	0,171165	1,028661
SRR1974617	neurons	1885028	1768887	1630401	1630401	1395922	85,61832334	0	0,077279	8560096	0	0,093906	0,15381	0,603789	0,148495	0,247716	0,245223	1,046057
SRR1974618	neurons	2931623	2784777	2614415	2614415	2285162	87,40624576	0	0,082436	13087590	0,00001	0,179314	0,208017	0,467504	0,145155	0,387331	0,383604	0,630488
SRR1974620	neurons	2615234	2460729	2280004	2280004	1950456	85,54616571	0	0,080485	11457657	0	0,042267	0,076013	0,711099	0,17062	0,11828	0,117061	1,648476
SRR1974621	neurons	953314	913569	867278	867278	757841	87,3815547	0	0,064428	5625899	0,000001	0,183315	0,174526	0,523859	0,118299	0,357841	0,35444	0,742856
SRR1974622	neurons	2566129	2425544	2250484	2250484	1946960	86,51294566	0	0,088725	10312814	0,000008	0,053929	0,103409	0,657734	0,184919	0,157338	0,155785	1,45705
SRR1974623	neurons	2735524	2593077	2420736	2420736	2085178	86,13818277	0	0,088069	11132519	0	0,048682	0,073053	0,722055	0,156211	0,121735	0,120653	1,634535
SRR1974624	neurons	3627176	3443021	3221150	3221150	2830582	87,8748894	0	0,082099	16281719	0	0,099515	0,122697	0,658302	0,119485	0,222213	0,220271	0,756223
SRR1974625	neurons	1693987	1591178	1474573	1474573	314118	21,30230243	0	0,198699	681837	0	0,246973	0,268089	0,135329	0,34961	0,515062	0,509728	1,539781
SRR1974626	neurons	2534013	2391856	2224052	2224052	1895975	85,24868124	0	0,08254	10844081	0,000001	0,067253	0,102029	0,681825	0,148892	0,169282	0,167704	1,37473
SRR1974627	neurons	3286717	3114440	2900449	2900449	2539905	87,56937288	0	0,083122	14419259	0,000001	0,059885	0,082619	0,730128	0,127367	0,142504	0,14125	1,132791
SRR1974628	neurons	3213844	3052791	2862464	2862464	2508563	87,6364908	0	0,079613	14906969	0,000012	0,094179	0,121143	0,674999	0,109668	0,215321	0,213427	0,770527
SRR1974629	neurons	2846479	2706101	2540068	2540068	2233497	87,93059871	0	0,071356	14896615	0,000003	0,105097	0,141589	0,641039	0,112272	0,246686	0,24445	0,739819
SRR1974630	neurons	3339755	3168370	2968300	2968300	2625844	88,46289122	0	0,077506	16052592	0	0,120369	0,151692	0,618854	0,109084	0,272062	0,269645	0,700333
SRR1974631	neurons	2907710	2748474	2554220	2554220	2229754	87,29686558	0	0,081568	12914215	0,000009	0,049397	0,080132	0,724596	0,145866	0,129529	0,128297	1,373243
SRR1974632	neurons	3394835	3229569	3037554	3037554	2647495	87,15877973	0	0,086654	14380441	0,000001	0,214484	0,274009	0,40104	0,110466	0,488493	0,483901	0,650301
SRR1974633	neurons	3107307	2927900	2699073	2699073	2328539	86,27180517	0	0,08328	13192701	0,000004	0,054563	0,079692	0,709126	0,156615	0,134255	0,132893	1,220318
SRR1974634	neurons	2546919	2417084	2267385	2267385	1980716	87,356845	0	0,075425	12461522	0,000003	0,176516	0,230065	0,461487	0,131929	0,406581	0,402707	0,658739
SRR1974635	neurons	3031458	2841692	2646713	2646713	2254742	85,19027186	0	0,089357	11853077	0	0,186686	0,24806	0,469364	0,09589	0,434746	0,430074	0,748957
SRR1974636	neurons	4471593	4212517	3899856	3899856	3364679	86,27700613	0	0,115219	13456699	0	0,055711	0,108083	0,674041	0,162166	0,163794	0,162306	1,617718
SRR1974638	neurons	3628993	3438703	3236825	3236825	2754356	85,09437489	0	0,106524	11992963	0,000002	0,194254	0,220225	0,465547	0,119972	0,414479	0,410484	0,900145
SRR1974639	neurons	4028657	3804603	3537444	3537444	3072773	86,86421608	0	0,116857	12101986	0,000001	0,163264	0,273441	0,455634	0,10766	0,436705	0,43277	0,772312
SRR1974640	neurons	5348502	5039792	4683531	4683531	4077209	87,05416917	0	0,109152	17291339	0	0,051479	0,076643	0,734961	0,136916	0,128122	0,1269	1,488832

SRR1974642	neurons	4011048	3770781	3488840	3488840	2983150	85,50549753	0	0,101991	13612274	0	0,042715	0,070403	0,717714	0,169168	0,113117	0,111929	1,708577
SRR1974643	neurons	6053839	5704158	5321095	5321095	4609925	86,63489376	0	0,108191	19738424	0,000003	0,130771	0,210898	0,500534	0,157794	0,341669	0,337883	0,796001
SRR1974645	neurons	4278882	4026765	3769378	3769378	3236324	85,85830341	0	0,084493	18056721	0,000006	0,090133	0,103448	0,682845	0,123569	0,193581	0,19156	0,854116
SRR1974646	neurons	4962965	4648648	4189246	4189246	3222914	76,93303282	0	0,284853	4522230	0,000001	0,124136	0,16264	0,380841	0,332383	0,286776	0,283568	1,792061
SRR1974648	neurons	2413906	2310995	2158400	2158400	1825316	84,56801334	0	0,082793	10406172	0,000003	0,028701	0,041083	0,528207	0,402006	0,069783	0,069168	1,549486
SRR1974649	neurons	4626194	4413830	4168922	4168922	3602844	86,42147778	0	0,112163	14835931	0,000002	0,195628	0,177754	0,486554	0,140062	0,373382	0,369364	0,72064
SRR1974650	neurons	3092532	2874136	2678260	2678260	2212102	82,59474435	0	0,110966	9215604	0,000003	0,061085	0,10463	0,650265	0,184017	0,165716	0,163497	1,859511
SRR1974652	neurons	3762490	3552877	3299270	3299270	2733410	82,84893325	0	0,120749	10387786	0,000006	0,112477	0,160761	0,605678	0,121078	0,273238	0,270505	1,29282
SRR1974654	neurons	4040890	3796796	3468016	3468016	3000275	86,51272082	0	0,132193	10324236	0	0,187506	0,258537	0,404753	0,149205	0,446042	0,441409	0,804405
SRR1974655	neurons	3296498	3106191	2888683	2888683	2471230	85,54867391	0	0,086251	13489578	0,000007	0,064056	0,079669	0,716001	0,140268	0,143725	0,142245	1,267198
SRR1974656	neurons	3102293	2941851	2761055	2761055	2369992	85,83646468	0	0,087257	12778498	0,000003	0,069625	0,087252	0,695315	0,147805	0,156877	0,155291	1,425503
SRR1974657	neurons	4245180	3979294	3639650	3639650	3116118	85,61586966	0	0,108322	13324923	0,000031	0,082673	0,123051	0,640637	0,153608	0,205724	0,203683	1,31809
SRR1974658	neurons	4086581	3850836	3608008	3608008	3090388	85,65357948	0	0,103684	13853871	0	0,161056	0,226645	0,502512	0,109786	0,387701	0,383765	0,747156
SRR1974659	neurons	3951135	3778308	3608620	3608620	3117040	86,37761804	0	0,100048	14520424	0,000003	0,204223	0,181819	0,499161	0,114793	0,386043	0,382259	0,759205
SRR1974660	neurons	3869448	3626415	3378490	3378490	2929645	86,71462695	0	0,091734	14975958	0	0,077156	0,098565	0,701907	0,122372	0,175721	0,174027	1,024689
SRR1974661	neurons	3909246	3698544	3442975	3442975	2995088	86,99127934	0	0,094588	14817411	0	0,03938	0,06523	0,714801	0,180588	0,10461	0,103622	1,60447
SRR1974662	neurons	3536012	3330535	3094522	3094522	2669007	86,24941106	0	0,086685	14491698	0	0,107397	0,164424	0,613383	0,114796	0,271822	0,268953	0,798131
SRR1974663	neurons	3150734	2978679	2779476	2779476	2268871	81,62945102	0	0,114643	9123645	0,000003	0,109465	0,160534	0,605523	0,124475	0,269999	0,267239	1,552217
SRR1974673	neurons	1949313	1649705	1530744	1530744	1003827	65,57771907	0	0,110664	4194278	0	0,043439	0,133514	0,686222	0,136825	0,176953	0,175754	2,779716
SRR1974674	neurons	3091012	2675820	2494267	2494267	2254258	90,37757385	0	0,087317	12145578	0	0,138908	0,213345	0,538315	0,109431	0,352253	0,350732	0,809147
SRR1974677	neurons	1771376	1570935	1455100	1455100	1319337	90,66985087	0	0,037013	17380296	0,000009	0,111682	0,15366	0,620947	0,113702	0,265342	0,264246	1,076202
SRR1974681	neurons	6317620	5921860	5688714	5688714	4097175	72,02286844	0	0,123193	15241147	0,000001	0,217309	0,290639	0,359923	0,132129	0,507948	0,502002	0,74261
SRR1974684	neurons	2417069	2258486	2166315	2166315	1642282	75,8099353	0	0,098074	7817377	0	0,135329	0,20034	0,548172	0,116158	0,335669	0,331634	0,761306
SRR1974686	neurons	9067868	8591035	8294661	8294661	6014513	72,51065475	0	0,12425	22162769	0,000035	0,198921	0,224782	0,4574	0,118863	0,423703	0,418762	0,684232
SRR1974697	neurons	6923302	6534059	6255604	6255604	4485168	71,69840035	0	0,094084	22326514	0,000005	0,054231	0,07514	0,716619	0,154004	0,129371	0,127823	1,209784
SRR1974704	neurons	7153830	6726417	6480198	6480198	4869307	75,14133056	0	0,104432	21665746	0,000002	0,154635	0,178072	0,541212	0,126079	0,332707	0,328833	0,668951
SRR1974705	neurons	7751646	7319943	7039639	7039639	4950765	70,32697273	0	0,133555	16851892	0	0,210341	0,232106	0,432741	0,124812	0,442447	0,437193	0,812861
SRR1974706	neurons	9039553	8614314	8321842	8321842	5984618	71,91458333	0	0,136642	19859205	0,000004	0,135427	0,14746	0,584458	0,132652	0,282886	0,279911	0,764986
SRR1974707	neurons	4936463	4603262	4334908	4334908	3004605	69,3118516	0	0,118641	11648025	0,000006	0,064637	0,108511	0,654058	0,172788	0,173148	0,171043	1,692129
SRR1974709	neurons	9006518	8538544	8238612	8238612	5812225	70,54859484	0	0,151392	17212184	0	0,213135	0,247938	0,419986	0,118941	0,461072	0,45593	0,771189
SRR1974710	neurons	5617948	5185446	4892762	4892762	3225960	65,93331129	0	0,163651	8752677	0,000001	0,049068	0,087471	0,629787	0,233673	0,136539	0,13474	2,042855
SRR1974711	neurons	5633435	5380896	5197953	5197953	2901765	55,82514886	0	0,174472	7320711	0,00009	0,125469	0,165249	0,51271	0,196482	0,290718	0,287529	1,940326
SRR1974712	neurons	5531513	5206747	5002308	5002308	3777250	75,51014452	0	0,099709	17670117	0	0,176759	0,191237	0,508621	0,123382	0,367997	0,36372	0,680526
SRR1974713	neurons	5299945	5017726	4855318	4855318	3574712	73,62467299	0	0,109082	15178664	0,000015	0,267691	0,285519	0,344528	0,102246	0,553211	0,546836	0,660253
SRR1974714	neurons	5993490	5608848	5382622	5382622	3808217	70,75022173	0	0,11015	16002475	0,000083	0,053934	0,088837	0,683154	0,173994	0,142772	0,14114	1,615973
SRR1974718	neurons	4504644	4060831	3863509	3863509	2840399	73,51863293	0	0,077787	17362688	0,00001	0,085068	0,099848	0,667752	0,147322	0,184917	0,18284	1,016947
SRR1974724	neurons	6723320	6346602	6128939	6128939	4496264	73,36121309	0	0,104116	20078539	0,000002	0,144644	0,178628	0,558396	0,118331	0,323272	0,319629	0,74534
SRR1974730	neurons	7451006	7047702	6780140	6780140	4795401	70,72716788	0	0,11067	20048304	0,000028	0,089652	0,095075	0,680319	0,134926	0,184728	0,182749	1,028838
SRR1974735	neurons	5628127	5322132	5138600	5138600	3763600	73,241739	0	0,087357	20278490	0,000001	0,159497	0,19403	0,528774	0,117698	0,353527	0,349143	0,715906
SRR1974752	neurons	7608158	7184606	6916940	6916940	5013447	72,48070679	0	0,108339	21449835	0,000013	0,151551	0,135979	0,554975	0,157482	0,28753	0,284363	0,765624
SRR1974756	neurons	1398119	1313357	1221339	1221339	1096417	89,77171776	0	0,056344	9360582	0	0,126192	0,217818	0,535928	0,120062	0,34401	0,341	0,829142
SRR1974759	neurons	1554200	1461379	1366222	1366222	1219730	89,27758446	0	0,044525	13287337	0	0,145384	0,199921	0,512978	0,141717	0,345305	0,342425	0,855801
SRR1974762	neurons	1579939	1504288	1427694	1427694	1257775	88,09836001	0	0,057843	10448959	0,000039	0,245091	0,370575	0,275668	0,108629	0,615665	0,610125	0,88744
SRR1974763	neurons	1771555	1662892	1556267	1556267	1399296	89,91362022	0	0,051504	13113668	0	0,166885	0,264382	0,447276	0,121457	0,431267	0,42742	0,79059
SRR1974767	neurons	1754929	1631318	1468353	1468353	1267098	86,29382717	0	0,148191	3841517	0,000001	0,152475	0,251279	0,328966	0,267279	0,403754	0,399886	1,519313
SRR1974773	neurons	1638333	1545756	1451410	1451410	1266246	87,24247456	0	0,065885	9182585	0	0,206999	0,295101	0,386403	0,111497	0,5021	0,497647	1,014143
SRR1974775	neurons	1758607	1656563	1553070	1553070	1377698	88,70804278	0	0,0565	11728315	0	0,183979	0,307911	0,410755	0,097355	0,49189	0,487411	1,037597
SRR1974776	neurons	1713239	1601924	1492226	1492226	1278008	85,64439971	0	0,08219	7342569	0	0,21992	0,331914	0,340728	0,107438	0,551834	0,546541	1,292399
SRR1974777	neurons	1904235	1792812	1686809	1686809	1501189	88,99579028	0	0,073239	9741861	0	0,242876	0,378299	0,280184	0,098641	0,621175	0,616035	0,883404
SRR1974778	neurons	2064601	1966325	1842452	1842452	1629367	88,43470549	0	0,075674	10215498	0,000001	0,198726	0,263578	0,412075	0,12562	0,462304	0,458473	0,98524
SRR1974779	neurons	1534279	1450278	1358075	1358075	1214234	89,40846419	0	0,063007	9226593	0	0,185685	0,302465	0,395947	0,115903	0,48815	0,484255	0,944794
SRR1974780	neurons	1589934	1456761	1340159	1340159	1141829	85,20100973	0	0,080534	6703165	0	0,214474	0,345677	0,297	0,142848	0,560151	0,554716	1,349448
SRR1974782	neurons	1826702	1722409	1614625	1614625	1443190	89,38236433	0	0,059087	11726426	0,000001	0,189162	0,297159	0,383938	0,12974	0,486321	0,482252	0,878456
SRR1974783	neurons	1917501	1811072	1700733	1700733	1524500	89,63782087	0	0,062278	11725826	0	0,229329	0,353878	0,315441	0,101351	0,583208	0,578461	0,891891
SRR1974785	neurons	2187628	2066684	1935028	1935028	1698491	87,77604252	0	0,079816	10065987	0	0,188274	0,356378	0,351579	0,10377	0,544652	0,54037	1,141621
SRR1974786	neurons	2137301	2017048	1877036	1877036	1649463	87,87593845	0	0,087331	8885539	0,000011	0,17153	0,22634	0,430514	0,171606	0,39787	0,394439	1,200393
SRR1974787	neurons	1758401	1657463	1540897	1540897	1322282	85,81248455	0	0,082871	7530808	0	0,187356	0,270852	0,427552	0,114239	0,458209	0,454343	1,333378
SRR1974793	neurons	999391	936275	865721	865721	735114	84,91349985	0	0,054239	6529272	0,000001	0,163947	0,228932	0,483197	0,123923	0,392879	0,389367	0,843713
SRR1974799	neurons	1901437	1813964	1707697	1707697	1426446	83,5303921	0	0,049405	13956863	0,000003	0,212627	0,290382	0,388091	0,108897	0,503009	0,498781	0,772311
SRR1974801	neurons	3379673	3271147	3064744	3064744	2629536	85,79953171	0	0,145418	8141716	0	0,107128	0,215788	0,554358	0,122726	0,322917	0,320632	1,027119
SRR1974803	neurons	2714860	2591705	2455044	2455044	2002426	81,56375201	0	0,051991	18584027	0,000002	0,123297	0,134003	0,603538	0,139161	0,2573	0,255003	1,073957
SRR1974806	neurons	2345846	2230864	2071905	2071905	1781965	86,00611515	0	0,050049	17203037	0,000005	0,124954	0,172737	0,575688	0,126617	0,297691	0,295402	0,770436
SRR1974807	neurons	2231192	2050015	1877471	1877471	1537787	81,90736368	0	0,060387	12214747	0,000002	0,102413	0,155256	0,532147	0,210183	0,257668	0,254582	1,23669
SRR1974808	neurons	3099812	2982790	2758841	2758841	1968661	71,3582624	0	0,16509	5286326	0,000001	0,107906	0,152226	0,505564	0,234303	0,260132	0,257995	2,098203
SRR1974809	neurons	2471261	2372204	2206632	2206632	1801844	81,65584474	0	0,116108	7146308	0,000006	0,205272	0,293753	0,331104	0,169865	0,499025	0,495055	0,949663
SRR1974810	neurons	1223693	1155947	1093607	1093607	845346	77,29888342	0	0,047388	8635401	0,000023	0,230189	0,173873	0,465151	0,130763	0,404062	0,399705	1,265279
SRR1974811	neurons	989915	941749	887619	887619	710268	80,01946781	0	0,038925	8885280	0	0,162301	0,145803	0,541373	0,150523	0,308104	0,305142	1,175565
SRR1974813	neurons	2452353	2351147	2233702	2233702	1848079	82,73614833	0	0,063015	14041167	0,000001	0,332889	0,272041	0,29979	0,095279	0,604929	0,599294	0,709776
SRR1974815	neurons	2631938	2534670	2347562	2347562	2042978	87,02551839	0	0,15703	5804588	0,000001	0,075073	0,195391	0,601243	0,128292	0,270464	0,268611	1,233458
SRR1974817	neurons	1764161	1674926	1566705	1566705	1350097	86,17429574	0	0,037091	17747084	0,000004	0,12046	0,170735	0,557417	0,151384	0,291195	0,288905	0,771769
SRR1974818	neurons	1536903	1467229	1357664	1357664	1160888	85,50628138	0	0,063445	8757518	0	0,091812	0,181262	0,598652	0,128275	0,273073	0,270911	0,975363
SRR1974819	neurons	2051541	1965774	1861841	1861841	1521010	81,69387182	0	0,047829	15389214	0,000002	0,233452	0,264196	0,367829	0,134522	0,497647	0,493356	0,696949
SRR1974820	neurons	2400746	2277584	2117461	2117461	1799659	84,99136466	0	0,057434	15061473	0	0,195603	0,254867	0,440165	0,109365	0,45047	0,446609	0,664674
SRR1974821	neurons	2489866	2361934	2192783	2192783	1812830	82,67256724	0	0,05447	16030843	0,000002	0,066835	0,134766	0,615581	0,182817	0,201601	0,199754	1,467858
SRR1974822	neurons	3093342	2970607	2766773	2766773	2372198	85,73880112	0	0,078926	14226319	0,000008	0,082419	0,164499	0,603271	0,149803	0,246918	0,245083	1,084097
SRR1974823	neurons	968161	917012	841107	841107	667524	79,36255435	0	0,069412	4583255	0	0,062427	0,198966	0,588647	0,14996	0,261393	0,258854	2,051064
SRR1974824	neurons	2331052	2227172	2086598	2086598	1746320	83,69221096	0	0,05051	16699656	0,000003	0,079184	0,135986	0,651384	0,133444	0,21517	0,213438	1,178336
SRR1974825	neurons	2510829	2386013	2236727	2236727	1827363	81,69807938	0	0,068565	12709437	0,000001	0,13498	0,200314	0,507597	0,157108	0,335294	0,332327	1,197458

SRR1974828	neurons	3035570	2887862	2702889	2702889	2302630	85,19143775	0	0,055604	19930648	0,000001	0,157058	0,216822	0,516003	0,110116	0,37388	0,370942	0,784393
SRR1974830	neurons	2467804	2380854	2223523	2223523	1897357	85,33111643	0	0,076935	11690009	0,000005	0,132294	0,217556	0,539949	0,110195	0,34985	0,347395	0,848639
SRR1974832	neurons	2603652	2473749	2314792	2314792	1952869	84,3647723	0	0,051015	18483423	0,000007	0,161335	0,208988	0,49689	0,13278	0,370323	0,367228	0,782802
SRR1974835	neurons	1599438	1522229	1425469	1425469	1202856	84,38317494	0	0,059614	9683644	0,000001	0,193845	0,298275	0,385869	0,12201	0,49212	0,48803	0,83658
SRR1974838	neurons	2663670	2552693	2377718	2377718	2003117	84,24535626	0	0,075521	12585555	0,000004	0,13033	0,238739	0,5017	0,129228	0,369069	0,366188	0,874519
SRR1974841	neurons	2982183	2856770	2656947	2656947	2286215	86,04669194	0	0,089588	11985602	0	0,099616	0,203977	0,566746	0,129661	0,303593	0,301319	0,991069
SRR1974842	neurons	2505232	2394252	2229474	2229474	1927980	86,47689993	0	0,071187	12891080	0,00001	0,144271	0,24128	0,493434	0,121004	0,385551	0,382604	0,788741
SRR1974843	neurons	3405228	3243333	2969044	2969044	2310165	77,80837872	0	0,1931	5184021	0,000001	0,112286	0,245774	0,428636	0,213303	0,35806	0,355081	1,560552
SRR1974844	neurons	2120572	2038363	1943055	1943055	1595491	82,1124981	0	0,054688	14050423	0	0,212902	0,20125	0,482008	0,103841	0,414152	0,410693	0,899185
SRR1974847	neurons	3568699	3412315	3165043	3165043	2592544	81,91180973	0	0,115273	10363319	0,000002	0,103396	0,164433	0,557608	0,174561	0,267829	0,265712	1,414421
SRR1974848	neurons	2163046	2048975	1895657	1895657	1619883	85,45232603	0	0,055151	14140841	0,000002	0,128215	0,213473	0,533308	0,125003	0,341687	0,338735	0,90563
SRR1974849	neurons	3287927	3182019	2977121	2977121	2504285	84,1176761	0	0,128517	8889033	0	0,105471	0,201082	0,55067	0,142778	0,306553	0,304469	1,151931
SRR1974853	neurons	1469748	1334468	1237798	1237798	1065419	86,07373739	0	0,032184	16195134	0	0,11015	0,160835	0,603024	0,125991	0,270985	0,268705	0,835649
SRR1974854	neurons	3985796	3845974	3594641	3594641	3077371	85,60996773	0	0,118657	11920021	0,000007	0,113048	0,206975	0,571235	0,108735	0,320023	0,31792	0,965514
SRR1974864	neurons	2317684	1933772	1749546	1749546	1413030	80,76552431	0	0,070414	9556983	0	0,156178	0,236096	0,476838	0,130889	0,392274	0,389755	1,407687
SRR1974866	neurons	2243813	1905796	1774850	1774850	1454551	81,95346086	0	0,053232	13173035	0,000002	0,051529	0,083462	0,703074	0,161933	0,134991	0,134221	2,137881
SRR1974867	neurons	1887600	1632462	1518948	1518948	1330566	87,59786378	0	0,03347	19430972	0	0,13007	0,14268	0,605121	0,12213	0,272749	0,271449	0,907979
SRR1974869	neurons	1183589	963133	880340	880340	599964	68,15139605	0	0,057507	5014496	0	0,056641	0,15867	0,639864	0,144825	0,215311	0,213282	2,23315
SRR1974870	neurons	1325476	1071756	970506	970506	565259	58,24374089	0	0,138423	1848656	0	0,095503	0,144891	0,417833	0,341773	0,240394	0,238183	2,591306
SRR1974872	neurons	1369293	1110722	1016501	1016501	665218	65,44194251	0	0,063167	5041372	0,000002	0,167829	0,262419	0,367952	0,201799	0,430248	0,426979	1,744753
SRR1974543	oligodendrocytes	2680235	2585008	2415025	2415025	2106633	87,23027712	0	0,280355	3015986	0,000001	0,141482	0,344028	0,424438	0,090051	0,485511	0,482196	1,532344
SRR1974545	oligodendrocytes	2627589	2511431	2342561	2342561	1987504	84,84321219	0	0,208711	4072854	0	0,116924	0,279943	0,517349	0,085784	0,396867	0,394077	1,821148
SRR1974548	oligodendrocytes	2698813	2597446	2388670	2388670	2025406	84,79220654	0	0,282347	2873895	0	0,065518	0,192234	0,59552	0,146728	0,257752	0,255694	2,316693
SRR1974549	oligodendrocytes	2133378	2031774	1893925	1893925	1673740	88,37414364	0	0,116933	6587290	0,000003	0,158173	0,320185	0,415886	0,105753	0,478358	0,474861	1,292889
SRR1974553	oligodendrocytes	1935800	1837429	1708706	1708706	1517886	88,83248493	0	0,113032	6198280	0,000032	0,14173	0,320372	0,445384	0,092482	0,462101	0,458578	1,474635
SRR1974561	oligodendrocytes	2847157	2753719	2579008	2579008	2290610	88,81748331	0	0,251092	3760644	0,000002	0,154992	0,354349	0,400546	0,090111	0,509341	0,50609	1,391732
SRR1974562	oligodendrocytes	2514707	2417752	2262881	2262881	2003855	88,55326462	0	0,165463	5367091	0	0,106775	0,26874	0,500489	0,123996	0,375516	0,373159	1,451307
SRR1974563	oligodendrocytes	2173354	2073937	1927109	1927109	1731682	89,85905831	0	0,119724	6642426	0	0,172535	0,358207	0,372615	0,096643	0,530742	0,526987	1,187625
SRR1974564	oligodendrocytes	3127747	3017406	2834616	2834616	2373313	83,72608494	0	0,262625	3686801	0,000002	0,154197	0,378805	0,345375	0,121621	0,533002	0,529607	1,529092
SRR1974566	oligodendrocytes	1532496	1456851	1345119	1345119	1210170	89,96750473	0	0,155865	3467270	0	0,080258	0,244693	0,567486	0,107563	0,324951	0,322739	1,496466
SRR1974570	oligodendrocytes	2017136	1919863	1751118	1751118	1454897	83,08389269	0	0,195681	3214833	0	0,247886	0,263267	0,243957	0,24489	0,511153	0,50726	1,390509
SRR1974571	oligodendrocytes	2921882	2820204	2631015	2631015	2352412	89,41081674	0	0,223414	4447254	0,000001	0,090021	0,256593	0,582251	0,071133	0,346614	0,344339	1,468159
SRR1974572	oligodendrocytes	2420738	2305722	2142284	2142284	1920765	89,65968098	0	0,107695	8265189	0	0,122361	0,228188	0,514593	0,134858	0,350549	0,34808	1,235379
SRR1974573	oligodendrocytes	3032024	2920979	2735452	2735452	2446209	89,42613506	0	0,21994	4711681	0,000006	0,106524	0,26057	0,542912	0,089988	0,367094	0,364532	1,379494
SRR1974574	oligodendrocytes	2538335	2417772	2253995	2253995	1981120	87,8937176	0	0,160912	5476122	0	0,170997	0,384615	0,358516	0,085873	0,555611	0,551713	1,250825
SRR1974616	oligodendrocytes	399914	347791	306791	306791	240195	78,29271393	0	0,034093	3442101	0	0,21911	0,212348	0,468193	0,100349	0,431459	0,425123	1,244749
SRR1974690	oligodendrocytes	8003880	7605381	7308229	7308229	4389817	60,06676857	0	0,195106	9736980	0	0,1456	0,167853	0,546458	0,140089	0,313453	0,310044	1,517923
SRR1974760	oligodendrocytes	2003724	1838202	1707559	1707559	1489078	87,20506876	0	0,088891	7871757	0	0,207704	0,314464	0,351818	0,126015	0,522167	0,517595	1,15681
SRR1974765	oligodendrocytes	1723473	1580145	1461243	1461243	1250773	85,59650927	0	0,081843	7218421	0	0,118693	0,238692	0,517235	0,125381	0,357384	0,353817	1,870071
SRR1974769	oligodendrocytes	2137381	2024424	1898889	1898889	1655957	87,20662451	0	0,066544	11884237	0	0,137672	0,216987	0,511736	0,133606	0,354658	0,351845	1,160104
SRR1974790	oligodendrocytes	1643902	1554191	1457594	1457594	1095200	75,13752115	0	0,073123	7119082	0	0,1603	0,194818	0,519469	0,125412	0,355118	0,352194	1,328722
SRR1974791	oligodendrocytes	1968871	1884993	1802260	1802260	1304870	72,4018732	0	0,123694	4830006	0	0,358977	0,424118	0,138672	0,078232	0,783096	0,777045	0,920096
SRR1974792	oligodendrocytes	2019163	1908821	1804158	1804158	1377194	76,33444521	0	0,062441	10564001	0	0,155439	0,187194	0,567002	0,090365	0,342633	0,339937	1,189499
SRR1974795	oligodendrocytes	929880	866970	814461	814461	603813	74,13651482	0	0,062405	4634414	0,000002	0,29965	0,370699	0,27826	0,05139	0,670349	0,664072	0,947655
SRR1974796	oligodendrocytes	1826021	1721409	1613501	1613501	1327557	82,27804011	0	0,062754	10130225	0,000002	0,197897	0,251946	0,449771	0,100385	0,449843	0,445989	0,899439
SRR1974797	oligodendrocytes	1650841	1545927	1460228	1460228	1064060	72,86944231	0	0,059421	8595325	0,00002	0,143265	0,18628	0,535868	0,134567	0,329545	0,326893	1,436768
SRR1974802	oligodendrocytes	2690742	2571547	2403833	2403833	1748101	72,72139953	0	0,172254	4473016	0	0,205655	0,495286	0,21671	0,082349	0,700941	0,694471	1,445455
SRR1974805	oligodendrocytes	3308063	3153106	2928129	2928129	2396103	81,83051361	0	0,125907	8698681	0,000011	0,139222	0,302714	0,472266	0,085787	0,441935	0,438211	1,255421
SRR1974812	oligodendrocytes	2880343	2744003	2540649	2540649	2160086	85,02103203	0	0,086511	11753553	0,000005	0,145206	0,262386	0,481426	0,110977	0,407592	0,404103	0,90447
SRR1974827	oligodendrocytes	2023199	1935620	1831289	1831289	1407318	76,84849306	0	0,088688	7457719	0,000009	0,278935	0,249838	0,377528	0,09369	0,528773	0,523638	1,152507
SRR1974829	oligodendrocytes	2201174	2094773	1951834	1951834	1623217	83,16368093	0	0,077942	9864552	0	0,223791	0,338026	0,331001	0,107182	0,561817	0,556585	0,768422
SRR1974831	oligodendrocytes	2818544	2709472	2552717	2552717	2006203	78,59088963	0	0,128316	7133322	0	0,272593	0,420716	0,251137	0,055553	0,693309	0,687777	1,044285
SRR1974834	oligodendrocytes	3177746	3045966	2859810	2859810	2267648	79,29365937	0	0,119051	8751962	0	0,183938	0,291931	0,442579	0,081551	0,475869	0,472032	1,108523
SRR1974836	oligodendrocytes	2818166	2678547	2502191	2502191	1958614	78,27595895	0	0,092859	9882717	0,000008	0,160165	0,259745	0,503103	0,076979	0,41991	0,416376	1,344218
SRR1974839	oligodendrocytes	3505305	3361790	3148847	3148847	2599488	82,55364583	0	0,132364	8932363	0,000019	0,121128	0,21545	0,584444	0,078959	0,336578	0,334095	1,289425
SRR1974845	oligodendrocytes	3377352	3240740	3038044	3038044	2409652	79,31590194	0	0,140846	7730592	0	0,14003	0,255482	0,525587	0,0789	0,395512	0,392534	1,479729
SRR1974851	oligodendrocytes	4219365	4055602	3769300	3769300	3192475	84,69676067	0	0,131929	11009881	0	0,111995	0,225861	0,569178	0,092965	0,337857	0,3355	1,075723
SRR1974852	oligodendrocytes	3762526	3620650	3389356	3389356	2790878	82,34242729	0	0,145981	8604144	0,000011	0,141028	0,222187	0,526307	0,110467	0,363215	0,360536	1,243209
SRR1974565	OPC	2298260	2199664	2044682	2044682	1867043	91,31214536	0	0,090271	9709182	0	0,08683	0,167096	0,647825	0,098249	0,253926	0,252169	1,196607
SRR1974575	OPC	2421223	2318813	2175857	2175857	1793296	82,41791625	0	0,134404	6059082	0	0,185824	0,225204	0,457141	0,131831	0,411028	0,408068	1,485844
SRR1974576	OPC	2354380	2241790	2071064	2071064	1828633	88,29437429	0	0,09309	9202378	0	0,059733	0,131586	0,675626	0,133055	0,19132	0,189861	1,763363
SRR1974578	OPC	3175092	3037597	2841218	2841218	2571959	90,52311368	0	0,088858	13601714	0,000002	0,093132	0,145875	0,6639	0,097091	0,239007	0,237369	1,262135
SRR1974579	OPC	2579389	2463075	2290915	2290915	2040262	89,05882584	0	0,08061	11965542	0	0,087517	0,150746	0,617462	0,144275	0,238263	0,23652	1,323542
SRR1974580	OPC	2636260	2527745	2347943	2347943	2077029	88,46164494	0	0,111774	8585120	0,000003	0,09578	0,180101	0,550927	0,173189	0,275881	0,273915	1,595004
SRR1974581	OPC	2211784	2112296	1980929	1980929	1731823	87,42478908	0	0,116143	6866368	0	0,114523	0,200012	0,614454	0,071011	0,314536	0,312346	1,655392
SRR1974583	OPC	2289818	2158542	1973609	1973609	1698581	86,06471697	0	0,136279	5651913	0,000002	0,077793	0,149542	0,619261	0,153401	0,227335	0,225373	2,241116
SRR1974585	OPC	2421195	2315843	2156043	2156043	1949263	90,40928219	0	0,091122	10035808	0	0,110111	0,189044	0,612305	0,088539	0,299156	0,296921	1,112833
SRR1974586	OPC	2257203	2128463	1959978	1959978	1703535	86,91602661	0	0,105174	7520232	0	0,079847	0,183217	0,601498	0,135437	0,263064	0,260927	1,923796
SRR1974587	OPC	2370593	2260769	2100393	2100393	1862428	88,67045358	0	0,093348	9344797	0,000001	0,062978	0,102536	0,713718	0,120768	0,165514	0,164307	1,950399
SRR1974588	OPC	2945305	2813803	2590647	2590647	2357578	91,00344431	0	0,143311	7419145	0	0,061977	0,12264	0,688687	0,126697	0,184616	0,183334	1,361994
SRR1974589	OPC	3130686	3005119	2766943	2766943	2465248	89,09645049	0	0,230557	4488359	0	0,085299	0,206925	0,579362	0,128414	0,292224	0,290186	1,578548
SRR1974592	OPC	2518298	2408981	2231358	2231358	1925329	86,28507841	0	0,166018	5137236	0,000001	0,072355	0,111726	0,632733	0,183186	0,18408	0,182774	1,938084
SRR1974593	OPC	3538222	3423164	3182641	3182641	2810849	88,3181295	0	0,255765	4511488	0	0,071306	0,152546	0,687212	0,088936	0,223852	0,22244	2,068503
SRR1974594	OPC	2722536	2601408	2416764	2416764	2175796	90,02931192	0	0,08805	11619006	0	0,091106	0,146613	0,610282	0,151999	0,237719	0,236055	1,353873
SRR1974596	OPC	2859962	2755860	2576054	2576054	2256275	87,58647917	0	0,158577	6340285	0,000008	0,070076	0,150996	0,655395	0,123524	0,221072	0,219727	1,639305
SRR1974600	OPC	2623283	2508718	2330965	2330965	2062475	88,48159453	0	0,143583	6476815	0	0,086839	0,190995	0,61772	0,104446	0,277833	0,275679	1,732061
**Mean**		2978980,97	2771779,22	2615128,35	2615128,35	2060085,402	80,86286625	0	0,102887351	10076457,71	4,31398E-06	0,141089471	0,209666333	0,522258596	0,126981342	0,350755776	0,348079634	1,295443505
