# Supplementary material for: Single-cell transcriptomics reveals specific RNA editing signatures in the human brain
Source: RNA. 2017 Jun;23(6):860–5. doi: 10.1261/rna.058271.116 (PMC5435858; doi:10.1261/rna.058271.116)
Supplement: Supplemental Material [file supp_058271.116_Supplemental_Table_2.pdf]

SRR	EditingDup	EditingNoDup	EditingCommon	DiffNumSiti	FractionOfCommon	PearsonR	Pvalue	Reads	Unique	Mapped
SRR1974543	125	117	117	8	0,936	0,999348394	9,47E-168	2415025	2106633	0,872302771
SRR1974544	314	291	287	23	0,914012739	0,999713305	0	2447300	2191073	0,89530217
SRR1974545	249	243	241	6	0,967871486	0,999913894	0	2342561	1987504	0,848432122
SRR1974546	483	455	452	28	0,935817805	0,999848833	0	2213973	1984558	0,896378592
SRR1974547	229	208	208	21	0,908296943	0,999875184	0	1802163	1568604	0,870400735
SRR1974548	210	194	190	16	0,904761905	0,999898262	0	2388670	2025406	0,847922065
SRR1974549	213	207	207	6	0,971830986	0,99990856	0	1893925	1673740	0,883741436
SRR1974550	640	591	589	49	0,9203125	0,998950656	0	2272309	2035235	0,895668239
SRR1974551	143	121	121	22	0,846153846	0,995084416	2,30E-121	1710100	1510766	0,883437226
SRR1974552	167	164	159	3	0,952095808	0,999879292	7,05E-286	2715974	2331876	0,858578175
SRR1974553	226	215	215	11	0,951327434	0,999876096	0	1708706	1517886	0,888324849
SRR1974554	516	489	489	27	0,947674419	0,999818754	0	2499116	2246933	0,899091119
SRR1974555	591	534	534	57	0,903553299	0,998820008	0	3064054	2807378	0,916229936
SRR1974556	297	264	263	33	0,885521886	0,99939444	0	1363613	1219972	0,894661462
SRR1974557	464	453	449	11	0,967672414	0,999854903	0	2232039	1923413	0,861729119
SRR1974558	716	665	663	51	0,925977654	0,999414598	0	3730747	3404060	0,912433891
SRR1974559	580	513	510	67	0,879310345	0,998730832	0	3597477	3320865	0,923109446
SRR1974560	428	409	409	19	0,955607477	0,999593493	0	2446670	2202628	0,900255449
SRR1974561	233	223	221	10	0,948497854	0,999784846	0	2579008	2290610	0,888174833
SRR1974562	244	236	232	8	0,950819672	0,999862314	0	2262881	2003855	0,885532646
SRR1974563	250	248	245	2	0,98	0,999027906	0	1927109	1731682	0,898590583
SRR1974564	253	240	239	13	0,944664032	0,999873031	0	2834616	2373313	0,837260849
SRR1974565	367	347	347	20	0,945504087	0,999580405	0	2044682	1867043	0,913121454
SRR1974566	154	145	145	9	0,941558442	0,999522739	7,43E-218	1345119	1210170	0,899675047
SRR1974567	453	428	427	25	0,942604857	0,99969778	0	3078216	2784851	0,904696422
SRR1974568	532	503	502	29	0,943609023	0,999530127	0	2486451	2245999	0,903295098
SRR1974569	304	292	290	12	0,953947368	0,999795729	0	2680450	2422665	0,903827715
SRR1974570	163	161	160	2	0,981595092	0,999960765	0	1751118	1454897	0,830838927
SRR1974571	332	324	316	8	0,951807229	0,999643227	0	2631015	2352412	0,894108167
SRR1974572	226	221	221	5	0,977876106	0,999772384	0	2142284	1920765	0,89659681
SRR1974573	313	304	301	9	0,961661342	0,999809549	0	2735452	2446209	0,894261351
SRR1974574	166	159	158	7	0,951807229	0,999697772	5,57E-253	2253995	1981120	0,878937176
SRR1974575	324	309	306	15	0,944444444	0,999896932	0	2175857	1793296	0,824179163
SRR1974576	361	342	341	19	0,944598338	0,999866329	0	2071064	1828633	0,882943743
SRR1974577	218	213	210	5	0,963302752	0,999953374	0	1636662	1272139	0,777276554
SRR1974578	577	532	531	45	0,920277296	0,999772977	0	2841218	2571959	0,905231137
SRR1974579	420	395	395	25	0,94047619	0,999768429	0	2290915	2040262	0,890588258
SRR1974580	322	310	309	12	0,959627329	0,999883521	0	2347943	2077029	0,884616449
SRR1974581	291	280	277	11	0,951890034	0,999782969	0	1980929	1731823	0,874247891
SRR1974582	309	296	296	13	0,957928803	0,99972202	0	2001564	1755866	0,877246993
SRR1974583	378	365	361	13	0,955026455	0,999793158	0	1973609	1698581	0,86064717
SRR1974584	371	343	343	28	0,924528302	0,99933684	0	2084261	1881159	0,902554431
SRR1974585	295	276	276	19	0,93559322	0,999571163	0	2156043	1949263	0,904092822
SRR1974586	408	388	388	20	0,950980392	0,999820089	0	1959978	1703535	0,869160266
SRR1974587	309	295	294	14	0,951456311	0,999693711	0	2100393	1862428	0,886704536
SRR1974588	460	416	416	44	0,904347826	0,999374351	0	2590647	2357578	0,910034443
SRR1974589	261	242	240	19	0,91954023	0,999802064	0	2766943	2465248	0,890964505
SRR1974590	214	218	212	4	0,990654206	0,999927319	0	2928717	2386697	0,814929199
SRR1974591	220	211	211	9	0,959090909	0,99978276	0	1717992	1439961	0,838165137
SRR1974592	274	261	260	13	0,948905109	0,999847182	0	2231358	1925329	0,862850784
SRR1974593	343	330	327	13	0,95335277	0,999775823	0	3182641	2810849	0,883181295
SRR1974594	537	509	508	28	0,945996276	0,999738699	0	2416764	2175796	0,900293119
SRR1974595	233	229	228	4	0,978540773	0,99980544	0	2118208	1878896	0,887021482
SRR1974596	375	367	365	8	0,973333333	0,999836954	0	2576054	2256275	0,875864792
SRR1974597	276	264	263	12	0,952898551	0,99970931	0	2398469	2126469	0,886594323
SRR1974598	186	176	173	10	0,930107527	0,999895704	1,21068691675e-316	1801814	1539580	0,854461115
SRR1974599	237	229	227	8	0,957805907	0,999939153	0	1661224	1378692	0,829925404
SRR1974600	352	338	337	14	0,957386364	0,99985492	0	2330965	2062475	0,884815945
SRR1974601	1225	1169	1169	56	0,954285714	0,999787317	0	2122929	1746115	0,822502778
SRR1974602	1050	951	948	99	0,902857143	0,998956536	0	2616933	2289470	0,874867641
SRR1974603	735	698	698	37	0,949659864	0,999900542	0	1985709	1557857	0,78453439
SRR1974604	957	911	908	46	0,948798328	0,99974548	0	2313431	1940442	0,838772369
SRR1974605	1915	1772	1771	143	0,924804178	0,999491659	0	2975040	2578482	0,866704985
SRR1974606	881	849	842	32	0,955732123	0,999867118	0	2841064	2194602	0,772457783
SRR1974607	1314	1254	1249	60	0,950532725	0,999749763	0	3511947	2992006	0,851950784
SRR1974608	1562	1435	1433	127	0,917413572	0,999462405	0	2819228	2476932	0,878585201
SRR1974609	1281	1139	1139	142	0,889149102	0,998907015	0	2926630	2591011	0,885322367
SRR1974610	1552	1481	1479	71	0,952963918	0,999597608	0	2498536	2143535	0,857916396
SRR1974611	1305	1244	1242	61	0,951724138	0,999727097	0	2090267	1636444	0,782887545
SRR1974612	874	793	793	81	0,907322654	0,99944141	0	1478928	1215409	0,82181756
SRR1974613	758	654	653	104	0,861477573	0,99847551	0	1657701	1429113	0,86210541
SRR1974614	834	796	795	38	0,95323741	0,999802161	0	1658699	1329587	0,801584254
SRR1974615	933	817	817	116	0,875669882	0,999169502	0	2292502	1984280	0,865552135
SRR1974617	505	435	435	70	0,861386139	0,999415529	0	1630401	1395922	0,856183233
SRR1974618	716	643	643	73	0,898044693	0,999028132	0	2614415	2285162	0,874062458
SRR1974619	1378	1304	1303	74	0,945573295	0,999691889	0	2304582	1930689	0,837761034
SRR1974620	1299	1199	1199	100	0,923017706	0,99964548	0	2280004	1950456	0,855461657
SRR1974622	1186	1078	1077	108	0,908094435	0,999346637	0	2250484	1946960	0,865129457
SRR1974623	1653	1551	1549	102	0,93708409	0,999556125	0	2420736	2085178	0,861381828
SRR1974624	2007	1886	1884	121	0,938714499	0,999158369	0	3221150	2830582	0,878748894
SRR1974626	1193	1106	1106	87	0,927074602	0,999575084	0	2224052	1895975	0,852486812
SRR1974627	1867	1707	1706	160	0,913765399	0,999300474	0	2900449	2539905	0,875693729
SRR1974628	1382	1274	1271	108	0,919681621	0,999076082	0	2862464	2508563	0,876364908
SRR1974629	911	803	802	108	0,880351262	0,998784883	0	2540068	2233497	0,879305987
SRR1974630	1278	1150	1148	128	0,89827856	0,998856494	0	2968300	2625844	0,884628912
SRR1974631	1619	1486	1486	133	0,917850525	0,999399655	0	2554220	2229754	0,872968656
SRR1974632	1145	1071	1066	74	0,931004367	0,999462061	0	3037554	2647495	0,871587797
SRR1974633	1690	1519	1518	171	0,898224852	0,999302793	0	2699073	2328539	0,862718052
SRR1974634	602	537	537	65	0,892026578	0,99899523	0	2267385	1980716	0,87356845
SRR1974635	1042	966	963	76	0,924184261	0,999666482	0	2646713	2254742	0,851902719
SRR1974636	2061	1970	1968	91	0,954876274	0,999700892	0	3899856	3364679	0,862770061
SRR1974637	1142	1113	1102	29	0,96497373	0,999902244	0	3791034	3147101	0,830143175
SRR1974638	1375	1311	1307	64	0,950545455	0,999843873	0	3236825	2754356	0,850943749

SRR1974639	1464	1368	1363	96	0,931010929	0,999702932	0	3537444	3072773	0,868642161
SRR1974640	3541	3330	3325	211	0,939000282	0,999621184	0	4683531	4077209	0,870541692
SRR1974641	815	795	774	20	0,949693252	0,999914969	0	4394381	3362601	0,765204701
SRR1974642	1964	1834	1833	130	0,933299389	0,999645061	0	3488840	2983150	0,855054975
SRR1974643	3338	3043	3037	295	0,909826243	0,999277708	0	5321095	4609925	0,866348938
SRR1974644	637	616	600	21	0,941915228	0,999732554	0	3477176	2785414	0,801056374
SRR1974645	2400	2202	2199	198	0,91625	0,99924434	0	3769378	3236324	0,858583034
SRR1974646	1079	1043	1033	36	0,957367933	0,999939022	0	4189246	3222914	0,769330328
SRR1974647	1460	1413	1408	47	0,964383562	0,999920859	0	3764739	3135038	0,832737143
SRR1974648	260	207	207	53	0,796153846	0,998887135	6,87E-274	2158400	1825316	0,845680133
SRR1974649	2184	2008	2005	176	0,918040293	0,999483055	0	4168922	3602844	0,864214778
SRR1974650	1797	1686	1684	111	0,937117418	0,999732668	0	2678260	2212102	0,825947443
SRR1974651	792	763	758	29	0,957070707	0,999755054	0	3274232	2691176	0,821925875
SRR1974652	1692	1625	1621	67	0,958037825	0,999844545	0	3299270	2733410	0,828489332
SRR1974653	1560	1478	1473	82	0,944230769	0,999662091	0	3240812	2783231	0,858806682
SRR1974654	1214	1146	1143	68	0,941515651	0,999575973	0	3468016	3000275	0,865127208
SRR1974655	1907	1756	1754	151	0,919769271	0,999505582	0	2888683	2471230	0,855486739
SRR1974656	2120	1934	1934	186	0,912264151	0,99938713	0	2761055	2369992	0,858364647
SRR1974657	1825	1748	1744	77	0,955616438	0,999685465	0	3639650	3116118	0,856158697
SRR1974658	1708	1639	1635	69	0,957259953	0,999658867	0	3608008	3090388	0,856535795
SRR1974659	1952	1826	1822	126	0,933401639	0,999420596	0	3608620	3117040	0,86377618
SRR1974660	1976	1826	1824	150	0,923076923	0,999307161	0	3378490	2929645	0,867146269
SRR1974661	2228	2069	2069	159	0,928635548	0,999557845	0	3442975	2995088	0,869912793
SRR1974662	1992	1836	1836	156	0,921686747	0,999247035	0	3094522	2669007	0,862494111
SRR1974663	1089	1037	1035	52	0,950413223	0,99990184	0	2779476	2268871	0,81629451
SRR1974665	630	620	618	10	0,980952381	0,999965036	0	1918412	1465715	0,764025142
SRR1974666	655	641	637	14	0,972519084	0,999928943	0	1744457	1349355	0,773510038
SRR1974667	1043	1022	1022	21	0,979865772	0,999906907	0	1571751	1276656	0,812250795
SRR1974671	492	486	483	6	0,981707317	0,999956187	0	1788595	1258260	0,703490729
SRR1974674	1562	1529	1526	33	0,976952625	0,999868213	0	2494267	2254258	0,903775739
SRR1974675	1278	1255	1254	23	0,981220657	0,999916482	0	1779749	1448026	0,813612481
SRR1974676	793	780	776	13	0,978562421	0,999934529	0	1813078	1461602	0,806144027
SRR1974677	1104	1079	1077	25	0,975543478	0,999752634	0	1455100	1319337	0,906698509
SRR1974678	4857	4750	4717	107	0,971175623	0,999760499	0	5506624	4004410	0,727198734
SRR1974679	5261	5129	5113	132	0,971868466	0,999786181	0	4998711	3576358	0,715456045
SRR1974680	2412	2348	2335	64	0,968076285	0,999755184	0	3557025	2522396	0,709130805
SRR1974681	3311	3227	3210	84	0,969495621	0,999801488	0	5688714	4097175	0,720228684
SRR1974682	5341	5159	5144	182	0,963115521	0,999736123	0	5034550	3685746	0,732090455
SRR1974683	6523	6313	6295	210	0,965046758	0,999740493	0	5882396	4335068	0,736956165
SRR1974684	2436	2249	2245	187	0,921592775	0,999434544	0	2166315	1642282	0,758099353
SRR1974685	6559	6340	6324	219	0,964171368	0,9997354	0	6935227	5064634	0,730276601
SRR1974686	6373	6125	6091	248	0,955750824	0,99960191	0	8294661	6014513	0,725106547
SRR1974687	5170	4955	4943	215	0,956092843	0,999575495	0	5217381	3964071	0,759781776
SRR1974688	3145	3089	3066	56	0,974880763	0,99985797	0	4819598	3479052	0,721855225
SRR1974689	2485	2399	2387	86	0,96056338	0,9997766	0	3238324	2335731	0,721277735
SRR1974691	6025	5834	5804	191	0,963319502	0,999742925	0	7398401	5391788	0,728777475
SRR1974692	5202	5063	5027	139	0,966359093	0,999829449	0	7152032	5135631	0,718065999
SRR1974693	2824	2758	2737	66	0,969192635	0,999822957	0	4517788	3185009	0,704993019
SRR1974694	2886	2755	2753	131	0,953915454	0,999630467	0	3050337	2229870	0,731024146
SRR1974697	9326	8946	8938	380	0,958395882	0,999542796	0	6255604	4485168	0,716984003
SRR1974700	4547	4391	4379	156	0,963052562	0,999616212	0	5508806	4044747	0,734232972
SRR1974701	4791	4669	4650	122	0,970569818	0,999789266	0	6032216	4347178	0,720660202
SRR1974704	6925	6498	6489	427	0,937039711	0,999347946	0	6480198	4869307	0,751413306
SRR1974705	4972	4815	4789	157	0,963193886	0,999867593	0	7039639	4950765	0,703269727
SRR1974706	6855	6559	6541	296	0,954194019	0,999478878	0	8321842	5984618	0,719145833
SRR1974709	3291	3232	3177	59	0,965360073	0,999819982	0	8238612	5812225	0,705485948
SRR1974712	3711	3546	3542	165	0,954459714	0,999765515	0	5002308	3777250	0,755101445
SRR1974713	3256	3169	3153	87	0,968366093	0,999683885	0	4855318	3574712	0,73624673
SRR1974714	5793	5589	5581	204	0,963404108	0,999812895	0	5382622	3808217	0,707502217
SRR1974718	4487	4342	4338	145	0,966792957	0,999686132	0	3863509	2840399	0,735186329
SRR1974720	3529	3427	3416	102	0,967979598	0,999797716	0	3903615	2776386	0,711234586
SRR1974723	5417	5287	5266	130	0,972124792	0,999808371	0	5996266	4303111	0,717631773
SRR1974724	5133	5007	4988	126	0,971751412	0,999720056	0	6128939	4496264	0,733612131
SRR1974726	3672	3556	3540	116	0,964052288	0,999798953	0	4093120	2981628	0,728448714
SRR1974729	6197	5945	5929	252	0,956753268	0,99963019	0	6122532	4587611	0,749299636
SRR1974730	6282	6166	6137	116	0,976918179	0,999836871	0	6780140	4795401	0,707271679
SRR1974732	7722	7499	7479	223	0,968531469	0,999733041	0	7147946	5278402	0,738450179
SRR1974733	3415	3338	3316	77	0,971010249	0,999847732	0	5196183	3685440	0,709259085
SRR1974735	5361	5108	5097	253	0,950755456	0,999479085	0	5138600	3763600	0,73241739
SRR1974736	2608	2534	2525	74	0,968174847	0,999725137	0	3417205	2470730	0,723026567
SRR1974739	5531	5360	5331	171	0,963840174	0,999725395	0	5898608	4390064	0,744254238
SRR1974745	5365	5238	5195	127	0,968313141	0,999771653	0	7189219	5220341	0,726134647
SRR1974746	7164	6899	6881	265	0,960496929	0,999637234	0	7775642	5593306	0,719336873
SRR1974747	5691	5500	5486	191	0,963978211	0,999650412	0	6774821	4974565	0,734272536
SRR1974748	4160	4025	4010	135	0,963942308	0,999804398	0	5784772	4106217	0,709832125
SRR1974749	6209	6041	6009	168	0,967788694	0,999772921	0	6298086	4429997	0,703387823
SRR1974750	2292	2228	2226	64	0,971204188	0,999864279	0	3365167	2499184	0,742662697
SRR1974751	6665	6458	6429	207	0,964591148	0,999714709	0	6632792	4906260	0,739697551
SRR1974752	4021	3942	3922	79	0,975379259	0,999849086	0	6916940	5013447	0,724807068
SRR1974755	457	439	439	18	0,960612691	0,999946464	0	1534957	1312289	0,85493535
SRR1974756	212	199	199	13	0,938679245	0,999641915	9,24243613335e-312	1221339	1096417	0,897717178
SRR1974757	321	311	309	10	0,962616822	0,999965473	0	1232702	1035339	0,839893989
SRR1974759	367	338	338	29	0,920980926	0,999676765	0	1366222	1219730	0,892775845
SRR1974760	400	389	388	11	0,97	0,999767675	0	1707559	1489078	0,872050688
SRR1974761	345	337	337	8	0,976811594	0,999930561	0	1316835	1114684	0,846487221
SRR1974762	532	513	513	19	0,964285714	0,999853539	0	1427694	1257775	0,8809836
SRR1974763	270	251	250	19	0,925925926	0,99982552	0	1556267	1399296	0,899136202
SRR1974764	591	579	577	12	0,976311337	0,999891922	0	1822578	1567186	0,859873213
SRR1974765	485	471	470	14	0,969072165	0,999940046	0	1461243	1250773	0,855965093
SRR1974766	589	577	575	12	0,9762309	0,999907134	0	1724174	1505086	0,872931618
SRR1974767	395	373	372	22	0,941772152	0,999967819	0	1468353	1267098	0,862938272
SRR1974768	311	301	299	10	0,961414791	0,999884376	0	1334148	1110064	0,832039624
SRR1974769	572	552	552	20	0,965034965	0,999910844	0	1898889	1655957	0,872066245
SRR1974770	216	212	209	4	0,967592593	0,999926983	0	1264550	1000594	0,791264877

SRR1974772	243	233	232	10	0,95473251	0,999330103	0	1496437	1340479	0,895780444
SRR1974773	522	497	497	25	0,95210728	0,999835988	0	1451410	1266246	0,872424746
SRR1974774	321	317	313	4	0,975077882	0,999936985	0	1504381	1259306	0,837092465
SRR1974775	610	585	585	25	0,959016393	0,999902672	0	1553070	1377698	0,887080428
SRR1974776	429	422	419	7	0,976689977	0,99990949	0	1492226	1278008	0,856443997
SRR1974777	426	416	413	10	0,969483568	0,99994241	0	1686809	1501189	0,889957903
SRR1974778	722	704	704	18	0,975069252	0,99991476	0	1842452	1629367	0,884347055
SRR1974779	370	352	352	18	0,951351351	0,999727222	0	1358075	1214234	0,894084642
SRR1974780	460	448	447	12	0,97173913	0,999976484	0	1340159	1141829	0,852010097
SRR1974782	706	680	680	26	0,963172805	0,99990627	0	1614625	1443190	0,893823643
SRR1974783	632	609	609	23	0,963607595	0,999926356	0	1700733	1524500	0,896378209
SRR1974784	401	385	385	16	0,960099751	0,999781832	0	1221002	1079544	0,884145972
SRR1974785	668	649	646	19	0,967065868	0,999925847	0	1935028	1698491	0,877760425
SRR1974786	688	668	668	20	0,970930233	0,999943744	0	1877036	1649463	0,878759384
SRR1974787	546	529	527	17	0,965201465	0,999945091	0	1540897	1322282	0,858124845
SRR1974790	435	424	423	11	0,972413793	0,999856059	0	1457594	1095200	0,751375211
SRR1974791	271	262	262	9	0,966789668	0,999777387	0	1802260	1304870	0,724018732
SRR1974792	526	509	509	17	0,967680608	0,999851399	0	1804158	1377194	0,763344452
SRR1974796	414	399	399	15	0,963768116	0,999665589	0	1613501	1327557	0,822780401
SRR1974797	365	356	356	9	0,975342466	0,999791595	0	1460228	1064060	0,728694423
SRR1974799	708	675	675	33	0,953389831	0,99973254	0	1707697	1426446	0,835303921
SRR1974801	1093	1026	1026	67	0,938700823	0,999374206	0	3064744	2629536	0,857995317
SRR1974802	469	462	459	7	0,978678038	0,999895559	0	2403833	1748101	0,727213995
SRR1974803	1955	1875	1874	80	0,958567775	0,999537434	0	2455044	2002426	0,81563752
SRR1974804	1267	1218	1218	49	0,961325967	0,999736456	0	3437125	2966224	0,862995672
SRR1974805	852	821	821	31	0,963615023	0,999863547	0	2928129	2396103	0,818305136
SRR1974806	963	917	917	46	0,952232606	0,999213864	0	2071905	1781965	0,860061151
SRR1974807	971	902	901	69	0,927909372	0,999664607	0	1877471	1537787	0,819073637
SRR1974808	833	803	802	30	0,962785114	0,999877509	0	2758841	1968661	0,713582624
SRR1974809	747	726	725	21	0,970548862	0,999858504	0	2206632	1801844	0,816558447
SRR1974812	741	703	703	38	0,948717949	0,999642277	0	2540649	2160086	0,85021032
SRR1974813	988	942	940	46	0,951417004	0,999638272	0	2233702	1848079	0,827361483
SRR1974815	409	375	374	34	0,914425428	0,999263556	0	2347562	2042978	0,870255184
SRR1974817	504	478	477	26	0,946428571	0,999055529	0	1566705	1350097	0,861742957
SRR1974818	212	195	195	17	0,919811321	0,999373001	5,39E-282	1357664	1160888	0,855062814
SRR1974819	620	589	587	31	0,946774194	0,999605698	0	1861841	1521010	0,816938718
SRR1974820	787	748	747	39	0,949174079	0,99978444	0	2117461	1799659	0,849913647
SRR1974821	1327	1259	1259	68	0,948756594	0,999727315	0	2192783	1812830	0,826725672
SRR1974822	1153	1085	1085	68	0,941023417	0,999675264	0	2766773	2372198	0,857388011
SRR1974824	1101	1047	1046	54	0,950045413	0,999702357	0	2086598	1746320	0,83692211
SRR1974825	1313	1270	1270	43	0,967250571	0,999792451	0	2236727	1827363	0,816980794
SRR1974826	662	650	649	12	0,980362538	0,999886452	0	2294121	1795222	0,782531523
SRR1974827	703	672	668	31	0,950213371	0,999930599	0	1831289	1407318	0,768484931
SRR1974828	1354	1307	1307	47	0,965288035	0,999703763	0	2702889	2302630	0,851914378
SRR1974829	531	504	504	27	0,949152542	0,999820163	0	1951834	1623217	0,831636809
SRR1974830	575	544	544	31	0,946086957	0,999344895	0	2223523	1897357	0,853311164
SRR1974831	454	444	442	10	0,973568282	0,999881772	0	2552717	2006203	0,785908896
SRR1974832	1139	1083	1083	56	0,950834065	0,999723971	0	2314792	1952869	0,843647723
SRR1974833	781	764	762	17	0,975672215	0,99977532	0	2243431	1879132	0,837615242
SRR1974834	690	680	680	10	0,985507246	0,999919947	0	2859810	2267648	0,792936594
SRR1974835	369	340	340	29	0,921409214	0,999566941	0	1425469	1202856	0,843831749
SRR1974836	576	561	559	15	0,970486111	0,999937518	0	2502191	1958614	0,782759589
SRR1974837	751	696	696	55	0,926764314	0,999419236	0	3171418	2736857	0,862975805
SRR1974838	757	735	734	22	0,969616909	0,999686575	0	2377718	2003117	0,842453563
SRR1974839	872	852	852	20	0,97706422	0,999884879	0	3148847	2599488	0,825536458
SRR1974840	1049	1018	1016	31	0,968541468	0,999876331	0	3064859	2547971	0,831350153
SRR1974841	873	833	833	40	0,954180985	0,99955002	0	2656947	2286215	0,860466919
SRR1974842	351	330	329	21	0,937321937	0,998424678	0	2229474	1927980	0,864768999
SRR1974843	788	763	763	25	0,968274112	0,99982316	0	2969044	2310165	0,778083787
SRR1974844	1152	1107	1107	45	0,9609375	0,999753913	0	1943055	1595491	0,821124981
SRR1974845	592	579	578	13	0,976351351	0,999906518	0	3038044	2409652	0,793159019
SRR1974846	1272	1230	1229	42	0,966194969	0,999795326	0	3131352	2660296	0,849567854
SRR1974847	1269	1224	1223	45	0,963750985	0,999825418	0	3165043	2592544	0,819118097
SRR1974848	676	644	643	32	0,951183432	0,99966159	0	1895657	1619883	0,85452326
SRR1974849	926	883	881	43	0,951403888	0,999776117	0	2977121	2504285	0,841176761
SRR1974850	1129	1108	1107	21	0,980513729	0,999833152	0	3006750	2544535	0,846274216
SRR1974851	816	781	781	35	0,957107843	0,999616786	0	3769300	3192475	0,846967607
SRR1974852	885	867	862	18	0,974011299	0,999873531	0	3389356	2790878	0,823424273
SRR1974853	179	173	172	6	0,960893855	0,999317777	1,76E-245	1237798	1065419	0,860737374
SRR1974854	1039	992	990	47	0,952839269	0,999679394	0	3594641	3077371	0,856099677
SRR1974855	1176	1142	1142	34	0,971088435	0,999861697	0	2627146	2150126	0,818426536
SRR1974856	1613	1583	1581	30	0,98016119	0,999820296	0	1665871	1286675	0,772373731
SRR1974862	1025	1010	1009	15	0,984390244	0,999948459	0	1416434	1063715	0,750980985
SRR1974864	1445	1411	1409	34	0,975086505	0,999834337	0	1749546	1413030	0,807655243
SRR1974865	1211	1191	1190	20	0,98265896	0,999928864	0	1541482	1134484	0,735969671
SRR1974866	2236	2189	2189	47	0,978980322	0,999833013	0	1774850	1454551	0,819534609
SRR1974867	1579	1535	1535	44	0,972134262	0,99983659	0	1518948	1330566	0,875978638
SRR1974868	1016	999	996	17	0,980314961	0,99990937	0	2367335	1912815	0,808003515
SRR1974871	1904	1866	1864	38	0,978991597	0,999912231	0	1729089	1472965	0,851873443
SRR1974873	1275	1250	1248	25	0,978823529	0,999916929	0	1684437	1406365	0,834916949
SRR1974874	1243	1214	1214	29	0,976669348	0,999888511	0	1389707	1107395	0,796855021
SRR1974875	965	940	940	25	0,974093264	0,999868069	0	2165526	1953500	0,9020903
SRR1974876	511	504	502	7	0,982387476	0,999944548	0	1614984	1330135	0,823621163
SRR1974877	467	466	460	1	0,985010707	0,999968213	0	2128466	1656240	0,77813787
SRR1974878	773	766	765	7	0,989650712	0,999893127	0	1665678	1457820	0,875211175
SRR1974879	652	641	636	11	0,975460123	0,999928081	0	2572313	2169027	0,843220479
SRR1974880	831	825	822	6	0,989169675	0,999933837	0	2428467	2068355	0,851712212
SRR1974881	555	545	545	10	0,981981982	0,999952625	0	1749428	1483066	0,847743377
SRR1974882	825	818	818	7	0,991515152	0,999907985	0	1507021	1340544	0,889532395
SRR1974883	932	915	914	17	0,980686695	0,99991476	0	1936106	1708377	0,882377824
SRR1974884	401	398	393	3	0,980049875	0,999979776	0	2784213	2006709	0,720745503
SRR1974885	980	966	964	14	0,983673469	0,999857583	0	2126996	1895530	0,89117704
SRR1974886	710	699	699	11	0,984507042	0,999937828	0	1986472	1708858	0,860247716
SRR1974887	809	789	787	20	0,972805933	0,999840697	0	2940657	2613148	0,888627269

SRR1974888	690	679	675	11	0,97826087	0,999924303	0	2257851	1867995	0,827333159
SRR1974890	821	804	803	17	0,978075518	0,999861846	0	2118938	1866873	0,881041824
SRR1974891	720	702	702	18	0,975	0,999862237	0	2159456	1901405	0,880501849
SRR1974892	642	632	631	10	0,982866044	0,999915338	0	1356970	1201153	0,885172848
SRR1974893	917	903	902	14	0,983642312	0,999956401	0	2336964	2040726	0,873238099
SRR1974894	522	513	512	9	0,980842912	0,99993635	0	1495271	1314056	0,878807922
SRR1974896	355	348	348	7	0,98028169	0,999916533	0	1977682	1614127	0,816171154
SRR1974897	827	814	809	13	0,978234583	0,999975439	0	2377042	1962617	0,825655163
SRR1974898	610	595	594	15	0,973770492	0,999960803	0	2069174	1676012	0,809990847
SRR1974900	665	650	647	15	0,972932331	0,99989149	0	2167126	1841750	0,849858292
SRR1974901	538	530	530	8	0,985130112	0,99992102	0	1649674	1359817	0,824294376
SRR1974902	576	566	566	10	0,982638889	0,999877984	0	1401512	1181177	0,842787646
SRR1974903	552	541	540	11	0,97826087	0,999865302	0	2004850	1644051	0,82003691
SRR1974905	642	632	632	10	0,984423676	0,999869608	0	1415078	1259560	0,890099344
SRR1974906	670	647	646	23	0,964179104	0,999954245	0	1888523	1523018	0,806459863
SRR1974907	492	488	488	4	0,991869919	0,999902354	0	1756839	1416888	0,80649849
SRR1974908	607	600	599	7	0,986820428	0,999948502	0	1708835	1252666	0,733052635
SRR1974909	709	700	700	9	0,987306065	0,999886055	0	1754605	1476876	0,841714232
SRR1974910	644	636	636	8	0,98757764	0,999928423	0	1401042	1216272	0,868119585
SRR1974911	962	946	945	16	0,982328482	0,999827613	0	1628877	1460691	0,896747268
SRR1974912	504	490	490	14	0,972222222	0,999946908	0	1462926	1255287	0,858065958
SRR1974913	986	972	971	14	0,984787018	0,999856336	0	1987772	1736696	0,873689739
SRR1974916	713	700	700	13	0,981767181	0,999856994	0	1723230	1548918	0,898845772
SRR1974917	649	639	639	10	0,98459168	0,999936351	0	1410788	1041712	0,738390176
SRR1974918	929	913	913	16	0,98277718	0,999945633	0	1780745	1534646	0,861799977
SRR1974919	668	653	653	15	0,97754491	0,999843198	0	1535813	1314129	0,855656906
SRR1974920	451	431	431	20	0,955654102	0,999826955	0	1864102	1546124	0,829420279
SRR1974921	1206	1176	1176	30	0,975124378	0,999715472	0	2229784	1968830	0,882968933
SRR1974922	572	555	555	17	0,97027972	0,999822889	0	1381671	1195459	0,865226961
SRR1974923	803	787	787	16	0,98007472	0,999813591	0	1654915	1468196	0,887173057
SRR1974924	689	671	670	18	0,972423803	0,999740381	0	1908329	1719361	0,900977242
SRR1974925	476	463	463	13	0,972689076	0,999836952	0	1567416	1384725	0,883444472
SRR1974926	867	834	834	33	0,961937716	0,999712551	0	1975359	1810659	0,916622751
SRR1974927	1035	1019	1016	16	0,981642512	0,999891817	0	2645130	2306552	0,871999486
SRR1974929	852	832	830	20	0,974178404	0,999733849	0	2246599	2012012	0,895581276
SRR1974930	1098	1078	1076	20	0,97996357	0,999857576	0	1925615	1727922	0,897335137
SRR1974931	1119	1098	1098	21	0,981233244	0,999866421	0	2137334	1905794	0,89166878
SRR1974932	415	401	401	14	0,96626506	0,999867707	0	1564207	1254393	0,801935422
SRR1974933	467	462	462	5	0,989293362	0,99990096	0	1676263	1359710	0,811155529
SRR1974935	874	853	853	21	0,97597254	0,999841985	0	1878660	1674595	0,891377365
SRR1974936	719	704	704	15	0,979137691	0,999827519	0	1626760	1409505	0,866449261
SRR1974937	1004	975	975	29	0,971115538	0,999867202	0	1977870	1748388	0,883975185
SRR1974938	379	371	371	8	0,978891821	0,999856964	0	1544875	1211032	0,783902905
SRR1974939	778	764	764	14	0,982005141	0,999880986	0	1510618	1319115	0,873228705
SRR1974940	854	836	836	18	0,978922717	0,999795246	0	1704404	1517360	0,890258413
SRR1974941	388	379	379	9	0,976804124	0,999605248	0	1814878	1580948	0,871104284
SRR1974942	1373	1336	1335	37	0,972323379	0,99990191	0	2213244	1991097	0,899628328
SRR1974944	607	590	590	17	0,97199341	0,999884613	0	1896787	1729577	0,911845663
SRR1974945	1004	991	991	13	0,987051793	0,999876565	0	2199880	1942377	0,882946797
SRR1974947	321	315	314	6	0,978193146	0,999953906	0	1698966	1269050	0,746954324
SRR1974948	413	401	399	12	0,966101695	0,99993886	0	2023955	1440107	0,711531136
SRR1974949	352	347	342	5	0,971590909	0,999935919	0	1977914	1516381	0,76665669
SRR1974950	257	254	250	3	0,972762646	0,999930724	0	1709059	1248854	0,73072609
SRR1974951	562	551	551	11	0,980427046	0,999938804	0	1887902	1508715	0,799149002
SRR1974952	550	543	540	7	0,981818182	0,999895893	0	2280617	1794241	0,786734906
SRR1974953	566	553	553	13	0,977031802	0,999965294	0	1375175	1055644	0,767643391
SRR1974954	612	604	604	8	0,986928105	0,9998105	0	1641145	1396060	0,85066219
SRR1974955	311	300	300	11	0,964630225	0,99925954	0	1801275	1594689	0,885311238
SRR1974956	752	743	739	9	0,982712766	0,999620168	0	2172127	1840198	0,847187112
SRR1974957	440	425	425	15	0,965909091	0,999922358	0	1804529	1337154	0,740998898
SRR1974959	314	309	305	5	0,97133758	0,999970175	0	1771156	1403053	0,79216794
SRR1974961	581	574	571	7	0,982788296	0,999848978	0	1937394	1500528	0,774508438
SRR1974962	600	595	589	5	0,981666667	0,999924079	0	2102197	1749309	0,832133715
SRR1974963	492	485	484	7	0,983739837	0,999887715	0	1511343	1315624	0,870499946
SRR1974964	462	451	450	11	0,974025974	0,999897604	0	1526879	1191502	0,780351292
SRR1974965	444	434	434	10	0,977477477	0,999845972	0	1632247	1401876	0,85886266
SRR1974966	673	668	666	5	0,989598811	0,999875183	0	2047739	1751435	0,855301872
SRR1974968	511	503	503	8	0,984344423	0,999885509	0	1829281	1360366	0,743661581
SRR1974969	595	587	586	8	0,98487395	0,9999614	0	1756446	1343080	0,764657724
SRR1974970	475	469	469	6	0,987368421	0,999884607	0	1762079	1420302	0,806037641
SRR1974971	442	431	431	11	0,975113122	0,999946609	0	1482748	1160761	0,782844421
SRR1974972	560	550	550	10	0,982142857	0,999904925	0	1612862	1330946	0,825207612
SRR1974973	541	534	534	7	0,987060998	0,999914789	0	1557217	1243465	0,798517483
SRR1974974	354	352	346	2	0,97740113	0,999859674	0	2017287	1486076	0,736670588
SRR1974977	529	516	516	13	0,975425331	0,999779728	0	1553242	1383020	0,890408578
SRR1974978	439	427	425	12	0,968109339	0,999817464	0	1468971	1253156	0,853084234
SRR1974981	642	615	615	27	0,957943925	0,999603265	0	2127565	1892124	0,889337811
SRR1974982	393	374	374	19	0,951653944	0,999693552	0	1405423	1238681	0,881358139
SRR1974983	307	291	290	16	0,944625407	0,999961584	0	1405614	1185083	0,843106998
SRR1974984	309	306	306	3	0,990291262	0,999887064	0	1490651	1226257	0,822631857
SRR1974985	378	363	362	15	0,957671958	0,999870183	0	1279273	1139943	0,891086578
SRR1974987	268	255	255	13	0,951492537	0,999953366	0	1374902	1085006	0,789151518
SRR1974988	299	290	290	9	0,969899666	0,999882212	0	1164744	1011414	0,868357339
SRR1974989	333	325	323	8	0,96996997	0,999734699	0	1751819	1437594	0,8206293
SRR1974990	383	360	360	23	0,939947781	0,999713564	0	1386354	1249098	0,900994984
SRR1974991	539	526	524	13	0,972170686	0,999930037	0	1610258	1408536	0,874726907
SRR1974992	526	510	509	16	0,967680608	0,999884659	0	1465963	1281699	0,87430515
SRR1974993	286	276	275	10	0,961538462	0,999871487	0	1569328	1325333	0,844522624
SRR1974994	296	280	280	16	0,945945946	0,999519883	0	1563386	1391260	0,88990179
SRR1974995	446	425	425	21	0,952914798	0,999848192	0	1552331	1353262	0,871761242
SRR1974996	627	586	586	41	0,93460925	0,999720199	0	1696976	1489421	0,877691258
SRR1974997	354	350	350	4	0,988700565	0,999771751	0	1399231	1235949	0,883305902
SRR1974998	663	638	637	25	0,960784314	0,999865511	0	1682065	1491510	0,886713653
SRR1974999	701	682	682	19	0,972895863	0,999891615	0	1836313	1634674	0,890193556

SRR1975000	555	531	531	24	0,956756757	0,999933255	0	1515606	1348581	0,889796557
SRR1975002	422	412	412	10	0,976303318	0,999869558	0	1575212	1363204	0,865409862
SRR1975003	313	306	304	7	0,971246006	0,999950209	0	1196251	1023376	0,855486014
SRR1975004	345	336	336	9	0,973913043	0,999912593	0	1207744	1055675	0,874088383
SRR1975005	420	408	407	12	0,969047619	0,999894555	0	1503837	1279895	0,851086255
SRR1975006	216	212	209	4	0,967592593	0,999964666	0	1464404	1133592	0,774097858
SRR1975007	605	579	579	26	0,957024793	0,999693276	0	1991849	1773147	0,890201516
SRR1975008	417	402	402	15	0,964028777	0,999879087	0	1486232	1275157	0,857979777
**Mean**	1256,172324			48,61357702	0,957885214			2585965,653	2111373,82	0,833500257
