# Supplementary material for: Single-cell transcriptomics reveals specific RNA editing signatures in the human brain
Source: RNA. 2017 Jun;23(6):860–5. doi: 10.1261/rna.058271.116 (PMC5435858; doi:10.1261/rna.058271.116)
Supplement: Supplemental Material [file supp_058271.116_Supplemental_Table_3.pdf]

Chr	Coordinate (hg19)	Location	GeneName	ChangeType	Aachange	TypeGenomicRegion
chr1	2436080	exonic	PLCH2	nonsyn	RG	NONREP
chr1	12091858	exonic	MIIP	nonsyn	SG	NONREP
chr1	21219204	exonic	EIF4G3	nonsyn	RG	NONREP
chr1	22970557	exonic	C1QC	nonsyn	KR	NONREP
chr1	35485995	exonic	ZMYM6	nonsyn	EG	NONREP
chr1	55075206	exonic	FAM151A	nonsyn	LP	NONREP
chr1	110256304	exonic	GSTM5	nonsyn	KR	NONREP
chr1	113242909	exonic	MOV10	nonsyn	QR	NONREP
chr1	155279696	exonic	FDPS	nonsyn	YC	NONREP
chr1	156703904	exonic	RRNAD1	nonsyn	QR	NONREP
chr1	160302244	exonic	COPA	nonsyn	IV	NONREP
chr1	160327023	exonic	NCSTN	nonsyn	IV	NONREP
chr1	240370872	exonic	FMN2	nonsyn	IM	NONREP
chr10	22022441	exonic	MLLT10	nonsyn	TA	NONREP
chr10	79397298	exonic	KCNMA1	nonsyn	SG	NONREP
chr10	79595574	exonic	DLG5	nonsyn	EG	NONREP
chr10	99424675	exonic	PI4K2A	nonsyn	IV	NONREP
chr10	101089862	exonic	CNNM1	nonsyn	SG	NONREP
chr10	102684025	exonic	FAM178A	nonsyn	SG	NONREP
chr11	6567089	exonic	DNHD1	nonsyn	IM	NONREP
chr11	8158982	exonic	RIC3	nonsyn	KR	NONREP
chr11	46388498	exonic	DGKZ	nonsyn	KR	NONREP
chr11	60775067	exonic	CD6	nonsyn	SG	NONREP
chr11	61724916	exonic	BEST1	nonsyn	IV	NONREP
chr11	62397341	exonic	GANAB	nonsyn	QR	NONREP
chr11	62416683	exonic	INTS5	nonsyn	KR	NONREP
chr11	62751512	exonic	SLC22A6	nonsyn	LP	NONREP
chr11	62752074	exonic	SLC22A6	nonsyn	LP	NONREP
chr11	71712486	exonic	IL18BP	nonsyn	KR	NONREP
chr11	105804694	exonic	GRIA4	nonsyn	RG	NONREP
chr12	5021742	exonic	KCNA1	nonsyn	IV	NONREP
chr12	7028883	exonic	ENO2	nonsyn	DG	NONREP
chr12	27181356	exonic	MED21	nonsyn	RG	NONREP
chr12	49176985	exonic	ADCY6	nonsyn	KR	NONREP
chr12	49315152	exonic	CCDC65	nonsyn	YH	NONREP
chr12	50475424	exonic	ASIC1	nonsyn	TA	NONREP
chr12	57864624	exonic	GLI1	nonsyn	RG	NONREP
chr12	69651595	exonic	CPSF6	nonsyn	RG	NONREP
chr12	109665188	exonic	ACACB	nonsyn	RG	NONREP
chr12	133696901	exonic	ZNF891	nonsyn	KR	NONREP
chr12	133696925	exonic	ZNF891	nonsyn	QR	NONREP
chr12	133696983	exonic	ZNF891	nonsyn	RG	NONREP
chr12	133697025	exonic	ZNF891	nonsyn	RG	NONREP
chr12	133697033	exonic	ZNF891	nonsyn	KR	NONREP
chr12	133697118	exonic	ZNF891	nonsyn	SG	NONREP
chr12	133697486	exonic	ZNF891	nonsyn	KR	NONREP
chr13	46090371	exonic	COG3	nonsyn	IV	NONREP
chr14	20920211	exonic	OSGEP	nonsyn	IM	NONREP
chr14	26917515	exonic	NOVA1	nonsyn	TA	NONREP
chr14	26917530	exonic	NOVA1	nonsyn	SG	NONREP

chr14	93142915	exonic	RIN3	nonsyn	MV	NONREP
chr14	95912401	exonic	SYNE3	nonsyn	KE	NONREP
chr15	65426067	exonic	PDCD7	nonsyn	QR	NONREP
chr15	75646086	exonic	NEIL1	nonsyn	KR	NONREP
chr15	80465406	exonic	FAH	nonsyn	KE	NONREP
chr15	82336645	exonic	MEX3B	nonsyn	QR	NONREP
chr16	1827756	exonic	SPSB3	nonsyn	KR	NONREP
chr16	20331702	exonic	GP2	nonsyn	EG	NONREP
chr16	23079791	exonic	USP31	nonsyn	KR	NONREP
chr16	30594639	exonic	ZNF785	nonsyn	RG	NONREP
chr16	57717870	exonic	GPR97	nonsyn	EG	NONREP
chr16	67237571	exonic	ELMO3	nonsyn	YH	NONREP
chr17	4693397	exonic	GLTPD2	nonsyn	WR	NONREP
chr17	16347053	exonic	FAM211A	nonsyn	LP	NONREP
chr17	19609985	exonic	SLC47A2	nonsyn	YH	NONREP
chr17	37826529	exonic	PNMT	nonsyn	SG	NONREP
chr17	38512342	exonic	RARA	nonsyn	EG	NONREP
chr17	42475813	exonic	GPATCH8	nonsyn	KR	NONREP
chr17	43045220	exonic	C1QL1	nonsyn	QR	NONREP
chr17	57697524	exonic	CLTC	nonsyn	EG	NONREP
chr17	64222153	exonic	APOH	nonsyn	ND	NONREP
chr17	73498270	exonic	CASKIN2	nonsyn	QR	NONREP
chr17	80441061	exonic	NARF	nonsyn	XW	ALU
chr18	32825609	exonic	ZNF397	nonsyn	KE	NONREP
chr18	32825654	exonic	ZNF397	nonsyn	IV	NONREP
chr18	32825741	exonic	ZNF397	nonsyn	RG	NONREP
chr19	7585273	exonic	ZNF358	nonsyn	KR	NONREP
chr19	9639621	exonic	ZNF426	nonsyn	KR	NONREP
chr19	14593605	exonic	GIPC1	nonsyn	TA	NONREP
chr19	20807628	exonic	ZNF626	nonsyn	YC	NONREP
chr19	21607515	exonic	ZNF493	nonsyn	YC	NONREP
chr19	44351711	exonic	ZNF283	nonsyn	KE	NONREP
chr19	44777091	exonic	ZNF233	nonsyn	VA	NONREP
chr19	44777106	exonic	ZNF233	nonsyn	LP	NONREP
chr19	44777178	exonic	ZNF233	nonsyn	LP	NONREP
chr19	44792527	exonic	ZNF235	nonsyn	KR	NONREP
chr19	44792765	exonic	ZNF235	nonsyn	SG	NONREP
chr19	44792894	exonic	ZNF235	nonsyn	RG	NONREP
chr19	44793030	exonic	ZNF235	nonsyn	IM	NONREP
chr19	44793215	exonic	ZNF235	nonsyn	ND	NONREP
chr19	44793216	exonic	ZNF235	nonsyn	IM	NONREP
chr19	46271684	exonic	SIX5	nonsyn	YC	NONREP
chr19	46526033	exonic	PGLYRP1	nonsyn	YH	NONREP
chr19	47152737	exonic	DACT3	nonsyn	SG	NONREP
chr19	53993629	exonic	ZNF813	nonsyn	DG	NONREP
chr19	54485579	exonic	CACNG8	nonsyn	SG	NONREP
chr19	56895363	exonic	ZNF582	nonsyn	RG	NONREP
chr19	56896203	exonic	ZNF582	nonsyn	ND	NONREP
chr19	58196667	exonic	ZNF551	nonsyn	QR	NONREP
chr19	58290138	exonic	ZNF586	nonsyn	KR	NONREP
chr2	84867621	exonic	DNAH6	nonsyn	KR	NONREP

chr2	208434976	exonic	CREB1	nonsyn	TA	NONREP
chr2	208434997	exonic	CREB1	nonsyn	TA	NONREP
chr2	208435003	exonic	CREB1	nonsyn	TA	NONREP
chr2	208435025	exonic	CREB1	nonsyn	QR	NONREP
chr2	208440032	exonic	CREB1	nonsyn	NS	NONREP
chr2	208440076	exonic	CREB1	nonsyn	TA	NONREP
chr2	210835613	exonic	UNC80	nonsyn	SG	NONREP
chr20	36147533	exonic	BLCAP	nonsyn	KR	NONREP
chr20	36147534	exonic	BLCAP	nonsyn	KE	NONREP
chr20	36147563	exonic	BLCAP	nonsyn	QR	NONREP
chr20	36147572	exonic	BLCAP	nonsyn	YC	NONREP
chr20	36393666	exonic	CTNNBL1	nonsyn	EG	NONREP
chr20	44439543	exonic	DNTTIP1	nonsyn	EG	NONREP
chr20	60737813	exonic	SS18L1	nonsyn	SG	NONREP
chr21	30953750	exonic	GRIK1	nonsyn	QR	NONREP
chr21	34922801	exonic	SON	nonsyn	TA	NONREP
chr21	34923275	exonic	SON	nonsyn	RG	NONREP
chr22	37962564	exonic	CDC42EP1	nonsyn	SG	NONREP
chr22	42992254	exonic	POLDIP3	nonsyn	TA	NONREP
chr3	9876560	exonic	TTLL3	nonsyn	KR	NONREP
chr3	14180692	exonic	TMEM43	nonsyn	RG	NONREP
chr3	48789722	exonic	PRKAR2A	nonsyn	QR	NONREP
chr3	53820892	exonic	CACNA1D	nonsyn	IM	NONREP
chr3	58141791	exonic	FLNB	nonsyn	MV	NONREP
chr3	58141801	exonic	FLNB	nonsyn	QR	NONREP
chr3	62423807	exonic	CADPS	nonsyn	EG	NONREP
chr3	148782634	exonic	HLTF	nonsyn	DG	NONREP
chr3	179093014	exonic	MFN1	nonsyn	IV	NONREP
chr3	179093066	exonic	MFN1	nonsyn	KR	NONREP
chr4	2835556	exonic	SH3BP2	nonsyn	RG	NONREP
chr4	17805279	exonic	DCAF16	nonsyn	IM	NONREP
chr4	57976234	exonic	IGFBP7	nonsyn	KR	NONREP
chr4	57976286	exonic	IGFBP7	nonsyn	RG	NONREP
chr4	76703983	exonic	USO1	nonsyn	KR	NONREP
chr4	77979680	exonic	CCNI	nonsyn	RG	NONREP
chr4	152200963	exonic	PRSS48	nonsyn	VA	NONREP
chr4	152200978	exonic	PRSS48	nonsyn	VA	NONREP
chr4	155507590	exonic	FGA	nonsyn	TA	NONREP
chr4	158257875	exonic	GRIA2	nonsyn	QR	NONREP
chr4	158281294	exonic	GRIA2	nonsyn	RG	NONREP
chr5	38949495	exonic	RICTOR	nonsyn	RG	NONREP
chr5	147862821	exonic	HTR4	nonsyn	QR	NONREP
chr5	156736808	exonic	CYFIP2	nonsyn	KE	NONREP
chr5	178413628	exonic	GRM6	nonsyn	CR	NONREP
chr6	27419515	exonic	ZNF184	nonsyn	HR	NONREP
chr6	33756242	exonic	LEMD2	nonsyn	SG	NONREP
chr6	34100903	exonic	GRM4	nonsyn	QR	NONREP
chr6	44120349	exonic	TMEM63B	nonsyn	QR	NONREP
chr6	73843194	exonic	KCNQ5	nonsyn	QR	NONREP
chr6	102337689	exonic	GRIK2	nonsyn	IV	NONREP
chr6	102337702	exonic	GRIK2	nonsyn	YC	NONREP

chr6	102372589	exonic	GRIK2	nonsyn	QR	NONREP
chr6	133072399	exonic	VNN2	nonsyn	KE	NONREP
chr7	39990299	exonic	CDK13	nonsyn	KR	NONREP
chr7	39990302	exonic	CDK13	nonsyn	KR	NONREP
chr7	39990344	exonic	CDK13	nonsyn	QR	NONREP
chr7	39990527	exonic	CDK13	nonsyn	KR	NONREP
chr7	39990548	exonic	CDK13	nonsyn	QR	NONREP
chr7	39990590	exonic	CDK13	nonsyn	KR	NONREP
chr7	39991045	exonic	CDK13	nonsyn	SG	NONREP
chr7	41729544	exonic	INHBA	nonsyn	SG	NONREP
chr7	100887329	exonic	FIS1	nonsyn	KR	NONREP
chr7	157333469	exonic	PTPRN2	nonsyn	EG	NONREP
chr8	10755755	exonic	XKR6	nonsyn	RG	NONREP
chr8	39027497	exonic	ADAM32	nonsyn	YC	NONREP
chr8	103841636	exonic	AZIN1	nonsyn	SG	NONREP
chr8	145579192	exonic	FBXL6	nonsyn	XW	NONREP
chr9	95027370	exonic	IARS	nonsyn	KR	NONREP
chr9	130438182	exonic	STXBP1	nonsyn	KE	NONREP
chr9	132571671	exonic	TOR1B	nonsyn	IV	NONREP
chrX	2825467	exonic	ARSD	nonsyn	RG	NONREP
chrX	49019255	exonic	MAGIX	nonsyn	ND	NONREP
chrX	49019262	exonic	MAGIX	nonsyn	KR	NONREP
chrX	100297260	exonic	TRMT2B	nonsyn	RG	NONREP
chrX	114082682	exonic	HTR2C	nonsyn	IV	NONREP
chrX	114082684	exonic	HTR2C	nonsyn	IM	NONREP
chrX	114082688	exonic	HTR2C	nonsyn	ND	NONREP
chrX	114082689	exonic	HTR2C	nonsyn	NS	NONREP
chrX	114082694	exonic	HTR2C	nonsyn	IV	NONREP
chrX	122598962	exonic	GRIA3	nonsyn	RG	NONREP
chrX	151358319	exonic	GABRA3	nonsyn	IM	NONREP
chrX	153579950	exonic	FLNA	nonsyn	QR	NONREP

ID	Symbol	Entrez Gene Name	Location	Type(s)
ACACB	ACACB	acetyl-CoA carboxylase beta	Cytoplasm	enzyme
ADAM32	ADAM32	ADAM metallopeptidase domain 32	Other	peptidase
ADCY6	ADCY6	adenylate cyclase 6	Plasma Membrane	enzyme
GPR97	ADGRG3	adhesion G protein-coupled receptor G3	Plasma Membrane	G-protein coupled receptor
APOH	APOH	apolipoprotein H	Extracellular Space	transporter
ARSD	ARSD	arylsulfatase D	Cytoplasm	enzyme
ASIC1	ASIC1	acid sensing ion channel subunit 1	Plasma Membrane	ion channel
AZIN1	AZIN1	antizyme inhibitor 1	Cytoplasm	enzyme
BEST1	BEST1	bestrophin 1	Plasma Membrane	ion channel
BLCAP	BLCAP	bladder cancer associated protein	Other	other
C1QC	C1QC	complement component 1, q subcomponent, C chain	Extracellular Space	other
C1QL1	C1QL1	complement component 1, q subcomponent-like 1	Extracellular Space	other
CACNA1D	CACNA1D	calcium channel, voltage-dependent, L type, alpha 1D subunit	Plasma Membrane	ion channel
CACNG8	CACNG8	calcium channel, voltage-dependent, gamma subunit 8	Plasma Membrane	ion channel
CADPS	CADPS	Ca++-dependent secretion activator	Plasma Membrane	other
CASKIN2	CASKIN2	CASK interacting protein 2	Cytoplasm	other
CCDC65	CCDC65	coiled-coil domain containing 65	Other	other
CCNI	CCNI	cyclin I	Other	other
CD6	CD6	CD6 molecule	Plasma Membrane	transmembrane receptor
CDC42EP1	CDC42EP1	CDC42 effector protein 1	Extracellular Space	other
CDK13	CDK13	cyclin-dependent kinase 13	Nucleus	kinase
CLTC	CLTC	clathrin, heavy chain (Hc)	Plasma Membrane	other
CNNM1	CNNM1	cyclin and CBS domain divalent metal cation transport mediator 1	Plasma Membrane	other
COG3	COG3	component of oligomeric golgi complex 3	Cytoplasm	transporter
COPA	COPA	coatomer protein complex subunit alpha	Cytoplasm	transporter
CPSF6	CPSF6	cleavage and polyadenylation specific factor 6	Nucleus	other
CREB1	CREB1	cAMP responsive element binding protein 1	Nucleus	transcription regulator
CTNNBL1	CTNNBL1	catenin beta like 1	Nucleus	other
CYFIP2	CYFIP2	cytoplasmic FMR1 interacting protein 2	Cytoplasm	other
DACT3	DACT3	dishevelled-binding antagonist of beta-catenin 3	Cytoplasm	other
DCAF16	DCAF16	DDB1 and CUL4 associated factor 16	Other	other
DGKZ	DGKZ	diacylglycerol kinase zeta	Cytoplasm	kinase
DLG5	DLG5	discs, large homolog 5 (Drosophila)	Plasma Membrane	other
DNAH6	DNAH6	dynein, axonemal, heavy chain 6	Extracellular Space	other
DNHD1	DNHD1	dynein heavy chain domain 1	Cytoplasm	other
DNTTIP1	DNTTIP1	deoxynucleotidyltransferase, terminal, interacting protein 1	Nucleus	ligand-dependent nuclear receptor
EIF4G3	EIF4G3	eukaryotic translation initiation factor 4 gamma, 3	Cytoplasm	translation regulator
ELMO3	ELMO3	engulfment and cell motility 3	Cytoplasm	other
ENO2	ENO2	enolase 2 (gamma, neuronal)	Cytoplasm	enzyme
FAH	FAH	fumarylacetoacetate hydrolase (fumarylacetoacetase)	Cytoplasm	enzyme
FAM151A	FAM151A	family with sequence similarity 151 member A	Extracellular Space	other
FBXL6	FBXL6	F-box and leucine-rich repeat protein 6	Other	enzyme
FDPS	FDPS	farnesyl diphosphate synthase	Cytoplasm	enzyme
FGA	FGA	fibrinogen alpha chain	Extracellular Space	other
FIS1	FIS1	fission, mitochondrial 1	Cytoplasm	other
FLNA	FLNA	filamin A	Cytoplasm	other
FLNB	FLNB	filamin B	Cytoplasm	other
FMN2	FMN2	formin 2	Cytoplasm	other
GABRA3	GABRA3	gamma-aminobutyric acid (GABA) A receptor, alpha 3	Plasma Membrane	ion channel
GANAB	GANAB	glucosidase, alpha; neutral AB	Cytoplasm	enzyme
GIPC1	GIPC1	GIPC PDZ domain containing family member 1	Cytoplasm	other
GLI1	GLI1	GLI family zinc finger 1	Nucleus	transcription regulator
GLTPD2	GLTPD2	glycolipid transfer protein domain containing 2	Other	other
GP2	GP2	glycoprotein 2	Cytoplasm	other
GPATCH8	GPATCH8	G-patch domain containing 8	Other	other
GRIA2	GRIA2	glutamate receptor, ionotropic, AMPA 2	Plasma Membrane	ion channel
GRIA3	GRIA3	glutamate receptor, ionotropic, AMPA 3	Plasma Membrane	ion channel
GRIA4	GRIA4	glutamate receptor, ionotropic, AMPA 4	Plasma Membrane	ion channel
GRIK1	GRIK1	glutamate receptor, ionotropic, kainate 1	Plasma Membrane	ion channel
GRIK2	GRIK2	glutamate receptor, ionotropic, kainate 2	Plasma Membrane	ion channel
GRM4	GRM4	glutamate receptor, metabotropic 4	Plasma Membrane	G-protein coupled receptor
GRM6	GRM6	glutamate receptor, metabotropic 6	Plasma Membrane	G-protein coupled receptor
GSTM5	GSTM5	glutathione S-transferase mu 5	Cytoplasm	enzyme
HLTF	HLTF	helicase-like transcription factor	Nucleus	transcription regulator
HTR2C	HTR2C	5-hydroxytryptamine (serotonin) receptor 2C, G protein-coupled	Plasma Membrane	G-protein coupled receptor
HTR4	HTR4	5-hydroxytryptamine (serotonin) receptor 4, G protein-coupled	Plasma Membrane	G-protein coupled receptor
IARS	IARS	isoleucyl-tRNA synthetase	Cytoplasm	enzyme
IGFBP7	IGFBP7	insulin like growth factor binding protein 7	Extracellular Space	transporter
IL18BP	IL18BP	interleukin 18 binding protein	Extracellular Space	other
INHBA	INHBA	inhibin beta A	Extracellular Space	growth factor

INTS5	INTS5	integrator complex subunit 5	Nucleus	other
KCNA1	KCNA1	potassium channel, voltage gated shaker related subfamily A	Plasma Membrane	ion channel
KCNMA1	KCNMA1	potassium channel, calcium activated large conductance	Plasma Membrane	ion channel
KCNQ5	KCNQ5	potassium channel, voltage gated KQT-like subfamily Q	Plasma Membrane	ion channel
LEMD2	LEMD2	LEM domain containing 2	Nucleus	other
FAM211A	LRRC75A	leucine rich repeat containing 75A	Other	other
MAGIX	MAGIX	MAGI family member, X-linked	Other	other
MED21	MED21	mediator complex subunit 21	Nucleus	transcription regulator
MEX3B	MEX3B	mex-3 RNA binding family member B	Other	kinase
MFN1	MFN1	mitofusin 1	Cytoplasm	enzyme
MIIP	MIIP	migration and invasion inhibitory protein	Other	other
MLLT10	MLLT10	myeloid/lymphoid or mixed-lineage leukemia; translocated to, 10	Nucleus	transcription regulator
MOV10	MOV10	Mov10 RISC complex RNA helicase	Nucleus	enzyme
NARF	NARF	nuclear prelamin A recognition factor	Nucleus	enzyme
NCSTN	NCSTN	nicastrin	Plasma Membrane	peptidase
NEIL1	NEIL1	nei-like DNA glycosylase 1	Nucleus	enzyme
NOVA1	NOVA1	neuro-oncological ventral antigen 1	Nucleus	other
OSGEP	OSGEP	O-sialoglycoprotein endopeptidase	Other	peptidase
PDCD7	PDCD7	programmed cell death 7	Nucleus	other
PGLYRP1	PGLYRP1	peptidoglycan recognition protein 1	Plasma Membrane	transmembrane receptor
PI4K2A	PI4K2A	phosphatidylinositol 4-kinase type 2 alpha	Cytoplasm	kinase
PLCH2	PLCH2	phospholipase C eta 2	Cytoplasm	enzyme
PNMT	PNMT	phenylethanolamine N-methyltransferase	Cytoplasm	enzyme
POLDIP3	POLDIP3	polymerase (DNA-directed), delta interacting protein 3	Nucleus	other
PRKAR2A	PRKAR2A	protein kinase, cAMP-dependent, regulatory subunit type II alpha	Cytoplasm	kinase
PRSS48	PRSS48	protease, serine 48	Other	other
PTPRN2	PTPRN2	protein tyrosine phosphatase, receptor type N2	Plasma Membrane	phosphatase
RARA	RARA	retinoic acid receptor, alpha	Nucleus	ligand-dependent nuclear receptor
RIC3	RIC3	RIC3 acetylcholine receptor chaperone	Cytoplasm	other
RICTOR	RICTOR	RPTOR independent companion of MTOR, complex 2	Cytoplasm	other
RIN3	RIN3	Ras and Rab interactor 3	Cytoplasm	other
RRNAD1	RRNAD1	ribosomal RNA adenine dimethylase domain containing 1	Other	other
SH3BP2	SH3BP2	SH3-domain binding protein 2	Cytoplasm	other
SIX5	SIX5	SIX homeobox 5	Nucleus	transcription regulator
SLC22A6	SLC22A6	solute carrier family 22 (organic anion transporter), member 6	Plasma Membrane	transporter
SLC47A2	SLC47A2	solute carrier family 47 (multidrug and toxin extrusion), member 2	Plasma Membrane	transporter
FAM178A	SLF2	SMC5-SMC6 complex localization factor 2	Extracellular Space	other
SON	SON	SON DNA binding protein	Nucleus	other
SPSB3	SPSB3	splA/ryanodine receptor domain and SOCS box containing 3	Other	other
SS18L1	SS18L1	synovial sarcoma translocation gene on chromosome 18-like 1	Nucleus	transcription regulator
STXBP1	STXBP1	syntaxin binding protein 1	Cytoplasm	transporter
SYNE3	SYNE3	spectrin repeat containing, nuclear envelope family member 3	Nucleus	other
TMEM43	TMEM43	transmembrane protein 43	Nucleus	other
TMEM63B	TMEM63B	transmembrane protein 63B	Plasma Membrane	other
TOR1B	TOR1B	torsin family 1, member B (torsin B)	Cytoplasm	enzyme
TRMT2B	TRMT2B	tRNA methyltransferase 2 homolog B	Cytoplasm	enzyme
TTLL3	TTLL3	tubulin tyrosine ligase like 3	Extracellular Space	enzyme
UNC80	UNC80	unc-80 homolog, NALCN activator	Plasma Membrane	enzyme
USO1	USO1	USO1 vesicle transport factor	Cytoplasm	transporter
USP31	USP31	ubiquitin specific peptidase 31	Nucleus	peptidase
VNN2	VNN2	vanin 2	Other	enzyme
XKR6	XKR6	X-linked Kx blood group related 6	Other	other
ZMYM6	ZMYM6	zinc finger, MYM-type 6	Nucleus	other
ZNF184	ZNF184	zinc finger protein 184	Nucleus	other
ZNF233	ZNF233	zinc finger protein 233	Nucleus	other
ZNF235	ZNF235	zinc finger protein 235	Nucleus	other
ZNF283	ZNF283	zinc finger protein 283	Nucleus	other
ZNF358	ZNF358	zinc finger protein 358	Nucleus	other
ZNF397	ZNF397	zinc finger protein 397	Nucleus	transcription regulator
ZNF426	ZNF426	zinc finger protein 426	Nucleus	other
ZNF493	ZNF493	zinc finger protein 493	Nucleus	other
ZNF551	ZNF551	zinc finger protein 551	Nucleus	other
ZNF582	ZNF582	zinc finger protein 582	Other	other
ZNF586	ZNF586	zinc finger protein 586	Other	other
ZNF626	ZNF626	zinc finger protein 626	Other	other
ZNF785	ZNF785	zinc finger protein 785	Other	other
ZNF813	ZNF813	zinc finger protein 813	Other	other
ZNF891	ZNF891	zinc finger protein 891	Other	other
